# Bisphosphonate-mineralized nano-IFNγ suppresses residual tumor growth caused by incomplete radiofrequency ablation through metabolically remodeling tumor-associated macrophages

**DOI:** 10.7150/thno.100998

**Published:** 2025-01-01

**Authors:** Zhicheng Yan, Bing Wang, Yuhan Shen, Junji Ren, Meifang Chen, Yunhui Jiang, Hao Wu, Wenbing Dai, Hua Zhang, Xueqing Wang, Qiang Zhang, Wei Yang, Bing He

**Affiliations:** 1 Beijing Key Laboratory of Molecular Pharmaceutics and Drug Delivery Systems, School of Pharmaceutical Sciences, Peking University, Beijing 100191, China.; 2 State Key Laboratory of Natural and Biomimetic Drugs, School of Pharmaceutical Sciences, Peking University, Beijing 100191, China.; 3 Key Laboratory of Carcinogenesis and Translational Research (Ministry of Education/Beijing), Department of Ultrasound, Peking University Cancer Hospital & Institute, Peking University, Beijing 100142, China.

**Keywords:** radiofrequency ablation, tumor immune microenvironment, tumor-associated macrophages, mevalonate metabolic pathway, interferon-γ

## Abstract

**Rationale:** Radiofrequency ablation (RFA), as a minimally invasive surgery strategy based on local thermal-killing effect, is widely used in the clinical treatment of multiple solid tumors. Nevertheless, RFA cannot achieve the complete elimination of tumor lesions with larger burden or proximity to blood vessels. Incomplete RFA (iRFA) has even been validated to promote residual tumor growth due to the suppressive tumor immune microenvironment (TIME). Therefore, exploring strategies to remodel TIME is a key issue for the development of RFA therapy.

**Methods:** The negative effect of iRFA on colorectal cancer therapy was firstly investigated. Then a zoledronate-mineralized nanoparticle loaded with IFNγ (Nano-IFNγ/Zole) was designed and its tumor suppressive efficacy was evaluated. Finally, the metabolic reprogramming mechanism of Nano-IFNγ/Zole on tumor-associated macrophages (TAMs) was studied in detail.

**Results:** We found iRFA dynamically altered TIME and promoted TAM differentiation from M1 to M2. Nano-IFNγ/Zole was fabricated to metabolically remodel TAMs. IFNγ in Nano-IFNγ/Zole concentrated in the ablation site to play a long-term remodeling role. Acting on mevalonate pathway, Nano-IFNγ/Zole was discovered to reduce lysosomal acidification and activate transcription factor TFEB by inhibiting isoprene modification of the Rab protein family. These mechanisms, in conjunction with IFNγ-activated JAK/STAT1 signaling, accelerated the reprogramming of TAMs from M2 to M1, and suppressed tumor recurrence after iRFA.

**Conclusions:** This study elaborates the synergistic mechanism of zoledronate and IFNγ in Nano-IFNγ/Zole to reshape suppressive TIME caused by iRFA by remodeling TAMs, and highlights the important value of metabolically induced cellular reprogramming. Since both zoledronate and IFNγ have already been approved in clinics, this integrative nano-drug delivery system establishes an effective strategy with great translational promise to overcome the poor prognosis after clinically incomplete RFA.

## Introduction

Radiofrequency ablation (RFA) is a clinical treatment that introduces radiofrequency electrodes into the tumor tissue under ultrasound or magnetic resonance guidance and generates intense friction within tumor lesions to produce high temperature (60-80 ℃) that results in coagulative necrosis of the tumor 1. The initial success of radiofrequency ablation on treating liver malignancies, such as hepatocellular carcinoma (HCC) and colorectal cancer liver metastasis (CRCLM), has extended its clinical and experimental applications to other types of neoplasms including tumors of the kidneys, breast, and lungs, as an alternative to surgery 1 ,2. Despite continuous developments in RFA techniques and instrumentation, the efficacy of RFA still faces the challenges owing to the diversity and heterogeneity of TIME 3 ,4. In the cases of tumors with larger burden or proximity to blood vessel, the thermal-killing effect induced by RFA may not be exerted on all tumor cells because of impeded heat transfer or heat pool effect, resulting in incomplete RFA (iRFA). Some tumor cells may survive the iRFA treatment, leading to tumor recurrence and even metastasis 3 ,4. Recent reports have shown that following iRFA, the microenvironment of the residual lesion may evolve into a more suppressive TIME, which can compromise the efficacy of subsequent adjuvant therapies using immune checkpoint inhibitors (ICIs) 5 ,6. Namely, iRFA may become an accomplice to tumor progression instead of a facilitator to immunotherapy. Therefore, exploring strategies of remodeling the suppressive TIME to overcome iRFA problems has become a key issue in the development of RFA therapy.

Tumor-associated macrophages (TAMs), one of the main drivers of suppressive TIME, are widely distributed in various types of solid malignancies 7 ,8. With the tumor progression, TAMs differentiate into an M2-dominated phenotype 8 ,9. These M2 macrophages induce the expression of immunosuppressive receptors, facilitate the proliferation of regulatory T cells but impair the activity of cytotoxic T cells, ultimately promoting tumor progression and accelerating tumor metastasis 9. In contrast, M1-type macrophages, which activate both innate and adaptive anti-tumor immunity, are inhibited in the suppressive TIME. Reprogramming M2-type TAMs in the TIME to M1 phenotype has become an effective strategy to enhance tumor-intrinsic anti-tumor immunity 10. However, little data are available to support its use for modifying the suppressive TIME after iRFA treatment.

**Figure 1:**
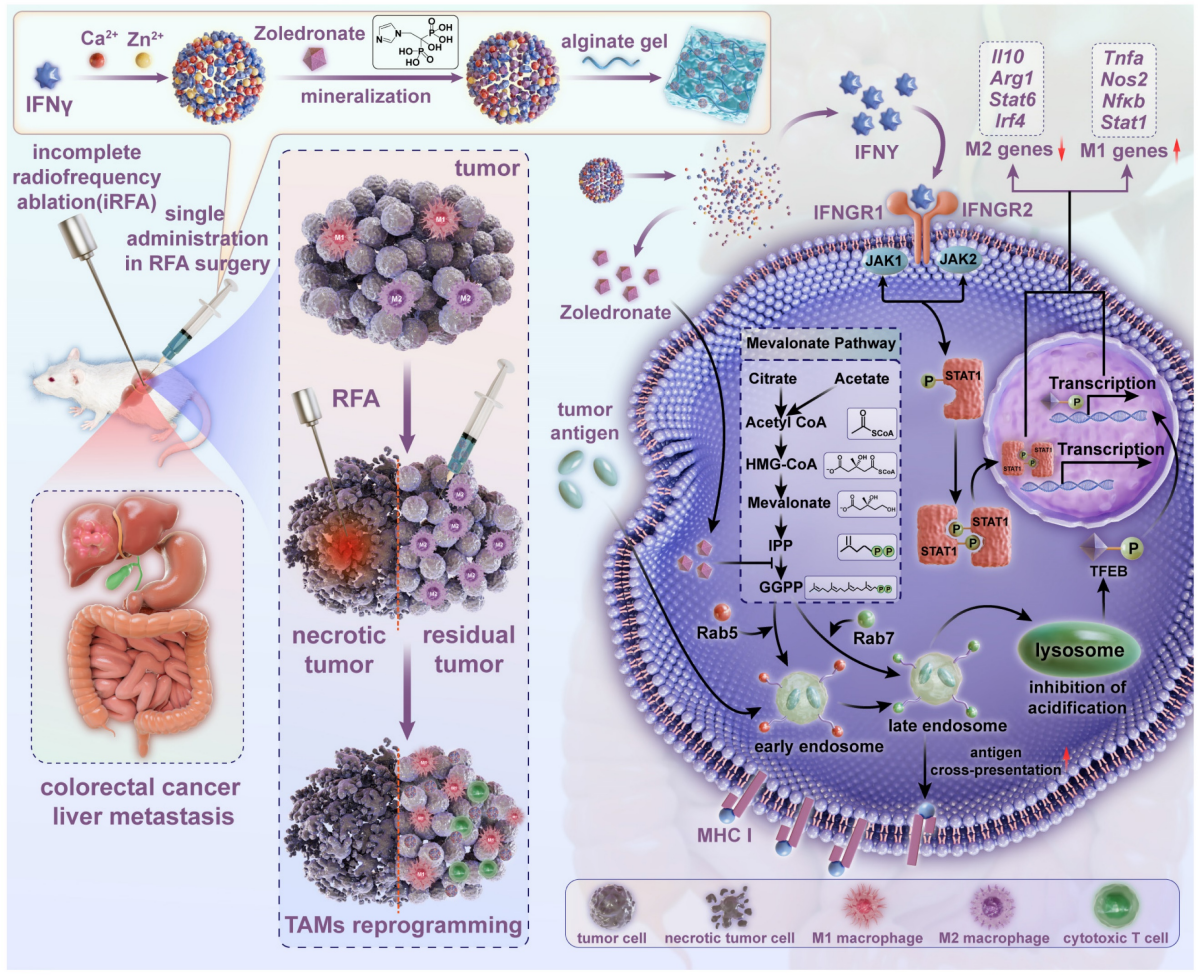
** Scheme of establishment and application of Nano-IFNγ/Zole for remodeling the suppressive TIME induced by iRFA.** Bisphosphonate-mineralized nano-IFNγ (Nano-IFNγ/Zole) was dispersed in alginate gel and injected into the peripheral tumor during the final stage of RFA surgery. A single administration of Nano-IFNγ/Zole can exert a long-term immune regulatory effect by retaining in residual tumors after iRFA. Zoledronate acts as an immune regulator by blocking the mevalonate metabolic pathway in TAMs, delaying the acidification process in intracellular lysosomes through blockade of isoprene modification of small GTPase in the mevalonate metabolic pathway. Inhibited lysosomal acidification activates TFEB signaling and promotes its nuclear translocation, which collaborates with IFNγ-mediated JAK/STAT1 pathway on reprogramming the immunosuppressive M2 TAMs to M1 type, thereby reshaping the suppressive TIME of CRCLM after iRFA and delaying the recurrence and metastasis of residual tumors.

In this study, nanoparticulated interferon-γ (nano-IFNγ) was designed and prepared using a strategy of bisphosphonate mineralization. This mineralized nano-IFNγ was dispersed in alginate gel and injected into the peripheral tumor during the final stage of RFA surgery (Figure 1). IFNγ promoted the trans-differentiation of TAMs from M2 to M1 through the IFNγ receptor-mediated JAK/STAT1 signaling pathway. Owing to the sustained from nanoparticle and gel, IFNγ could remain in the residual tumor site for a long time and exert its immune-regulatory effect, while its systemic toxicity and adverse reactions in the normal peripheral tissues were avoided. We further investigated the effect of the bisphosphonate-induced inhibition of mevalonate metabolic pathway on TIME. Mechanistically, we found that the internalization of bisphosphonates by the TAMs resulted in the blockade of isoprene modification of small GTPases in the mevalonate metabolic pathway, which in turn resulted in a delay in the acidification process in intracellular lysosomes. The inhibited lysosomal acidification activated transcription factor TFEB, in conjunction with JAK/STAT1 signaling activated by IFNγ accelerated the differentiation of TAMs from the M2 to M1 type. After a series of *in vivo* pharmacodynamic investigations, this reprogramming process was demonstrated to remodel the suppressive TIME after iRFA and increase the therapeutic sensitivity of ICIs, constituting a potential strategy for overcoming the poor prognosis of iRFA in clinical practice.

## Materials and methods

### Cell lines and animals

Murine cell lines--CT26 cell line, DC2.4 cell line, RAW264.7 cell line and B16-OVA cell line were originally obtained from American Type Culture Collection (ATCC). CT26 cells and DC2.4 cells were cultured in complete RPMI 1640 medium (M&C GENE) containing 10% FBS (Gemini), 1% penicillin (M&C GENE) and 1% streptomycin (M&C GENE) at 37 °C in humidified air with 5% CO_2_. RAW264.7 cells and B16-OVA cells were cultured in complete DMEM medium (M&C GENE) containing 10% FBS (Gemini), 1% penicillin (M&C GENE) and 1% streptomycin (M&C GENE) at 37 °C in 5% CO_2_ humidified air. Female BALB/c mice and male C57BL/6 mice (6-8 weeks) were ordered from Laboratory Animal Science Department of Peking University Health Science Center. Mice were housed in an SPF-grade pathogen-free facility with a 12 h light/dark cycle at 20 ± 3 °C and a relative humidity of 40% to 70% before sacrifice.

### Preparation and characterization of Nano-IFNγ/Zole

Nano-IFNγ/Zole was fabricated by nanoprecipitation method as previously reported with minor modifications 11. Briefly, IFNγ (50 μg/mL), CaCl_2_/ZnCl_2_ (250 mM and 500 mM, respectively), and zoledronate solution (50 mM) were separately dispersed in mixed solvent (IGEPAL®CA-630: cyclohexane, 6.5: 3.5, v/v) under vigorous stirring. Then, IFNγ system was added dropwise into the CaCl_2_/ZnCl_2_ system under gentle stirring at room temperature. Next, the mixed system was added to the bisphosphonate system under gentle stirring at room temperature. Next, equivalent volume of ethanol was quickly added to the mixture under gentle stirring to form stable precipitation. The precipitation was centrifuged, washed, and resuspended with deionized water and probe ultrasound was performed to obtain the nanoparticles. The particle size and zeta potential of Nano-IFNγ/Zole were characterized using Malvern Zetasizer (Nano ZS, Malvern, UK). The morphologies and element mapping of the Nano-IFNγ/Zole were observed using TEM (Tecnai G2 F30 S-TWIN, FEI, USA) and SEM (JSM-7900F, JEOL, Japan). Circular dichroism spectrum was determined using circular binary dispersion spectrometer (J-1500, Jasco, Japan). Thermogravimetric analysis was performed on thermal gravimetric analyzer (STA 449 F3, NETZSCH, Germany). The interaction between components of Nano-IFNγ/Zole was determined by Isothermal Titration Calorimetry (MicroCal PEAQ-ITC, Malvern, UK).

### Mouse model construction and iRFA treatment

A density of 5×10^5^ CT26 colorectal cancer cells were subcutaneously injected into female BALB/c mice (6-8 weeks) on the right flank. Twelve days after tumor inoculation, the tumor-bearing mice were randomly divided into different groups for iRFA treatment. Radiofrequency ablation (65 ℃, 20 W, 45 s) was performed on majority of the tumor tissue with sterile instruments, leaving minor residual tumor tissue alive. The local temperature of tumor tissue being ablated was between 55-75 ℃ (Figure S1). Tumor size monitoring of mice was conducted every other day after iRFA treatment. The tumor volume (mm^3^) was calculated according to (length × width^2^) × 0.5. Mice of all groups were euthanized on day 16-20, and tumor tissues were isolated for following experiments.

### Statistical analysis

Data were analyzed by two-tailed unpaired t tests or one-way ANOVA with Tukey's multiple comparisons within GraphPad Prism 8 software, and quantitative statistics were represented as mean ± standard deviation (SD). Statistical significance was set as *P <0.05, **P < 0.01, ***P < 0.001, ns, no significance.

The Supplementary Materials include further methodological details.

## Results

### Transformation of TAMs from M1 to M2 induced by incomplete RFA leads to a suppressive TIME and impairs the therapeutic efficacy of ICIs

**Figure 2:**
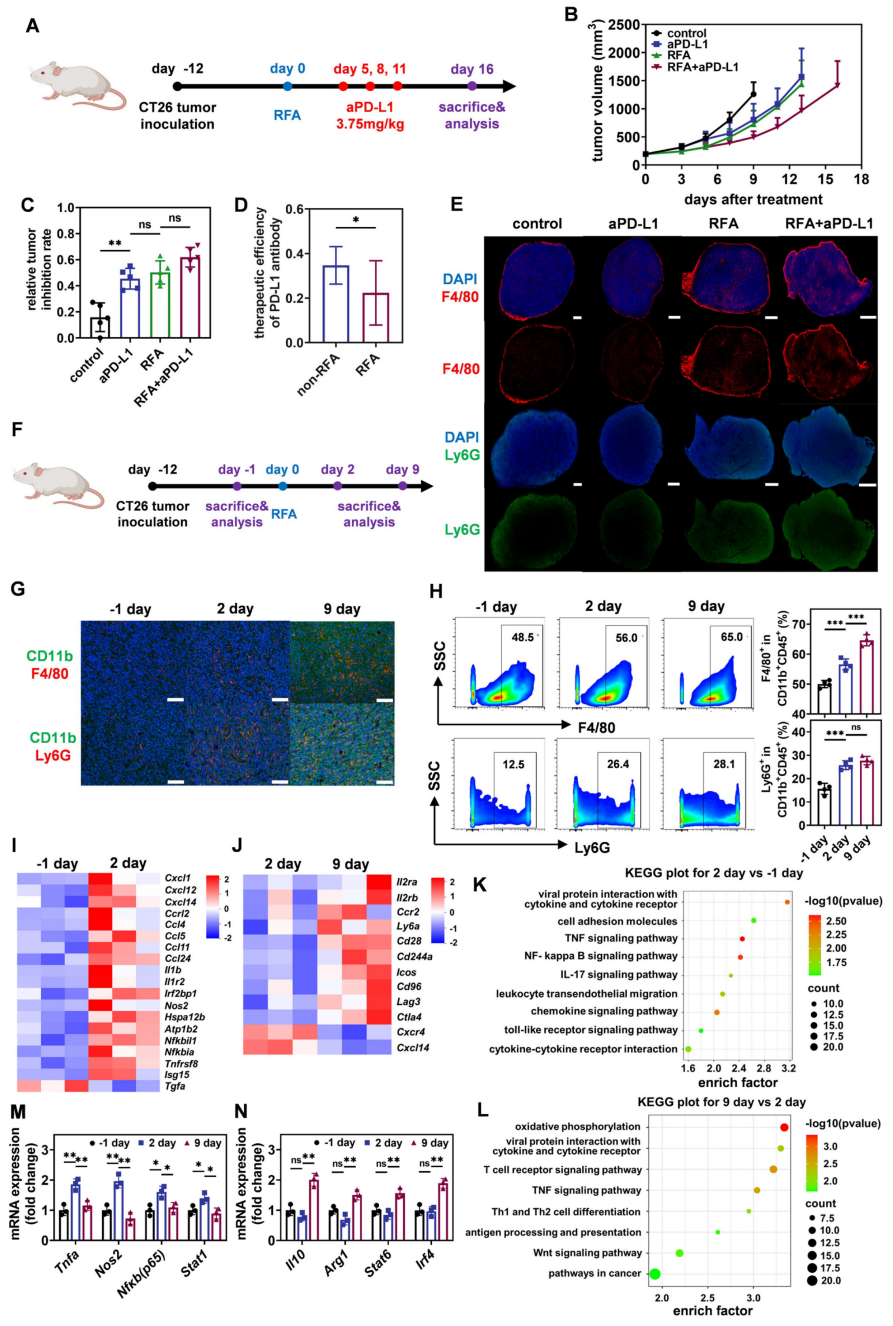
** Transformation of TAMs induced by iRFA leads to a suppressive TIME and impairs the therapeutic efficacy of ICIs.** (A) Schematic illustration of the assessment of the influence of iRFA on the therapeutic efficacy of PD-L1 antibody. (B) Growth curves of tumor volume after different treatment (n = 5). (C) Therapeutic efficiency of PD-L1 antibody with or without iRFA. (D) Relative tumor inhibition rate based on tumor weight after different treatment (n = 5). (E) Immunofluorescence images of tumor-infiltrating macrophages and neutrophils, scale bar: 1 mm. (F) Schematic illustration of the assessment of tumor immune microenvironment before and after iRFA treatment. (G) Representative immunofluorescence images of tumor-infiltrating macrophages and neutrophils before and after iRFA, scale bar: 100 μm. (H) Representative FCM plots and corresponding quantification of tumor-infiltrating macrophages and neutrophils before and after iRFA (n = 4). (I) Heatmap of differentially expressed genes in tumor tissue 1 day before and 2 days after iRFA (n = 3). (J) Heatmap of differentially expressed genes in tumor tissue 2 days and 9 days after iRFA (n = 3). (K) KEGG enrichment analysis of differentially expressed genes in tumor tissue 1 day before and 2 days after iRFA (n = 3). (L) KEGG enrichment analysis of differentially expressed genes in tumor tissue 2 days and 9 days after iRFA (n = 3). (M and N) qRT-PCR analysis of M1-type genes (M) or M2-type genes(N) expressed in tumor tissue before and after iRFA treatment (n = 4). All statistical data are presented as mean ± SD; data were analyzed with two-tailed unpaired t tests; ns, no significance; *, p < 0.05; **, p < 0.01; ***, p < 0.001.

To verify the resistance of residual tumors after iRFA to the subsequent adjuvant therapy, we firstly constructed a mouse model of CT26-loaded colorectal cancer and treated it with iRFA (Figure 2A). Literature has reported that CT26 tumor cells possess relatively low abundance of PD-L1 molecules, which would to some extent compromise the efficacy of ICIs 12 ,13. Animal experiments using CT-26 bearing mouse model demonstrated that iRFA-treated tumors were more sensitive to anti-PD-L1 antibody (aPD-L1) than to anti-PD-1 antibody (aPD-1) (Figure S2). Furthermore, immunofluorescence and quantitative reverse transcription-polymerase chain reaction (qRT-PCR) results revealed that iRFA-treated CT26 tumors exhibited a higher *Pdl1* mRNA expressing profile compared to the no-treated counterparts, while the *Pd1* mRNA expression on CT26 tumors shows no significant changes between iRFA-treated group and no-treated group (Figure S3). Thus, a PD-L1 monoclonal antibody was subsequently administered to evaluate the effect of the TIME on immunotherapy before and after iRFA. As shown in Figure 2B, both iRFA and PD-L1 monoclonal antibody treatments reduced tumor growth compared to no treatment group. Notably, although the combination of iRFA and aPD-L1 effectively inhibited tumor proliferation, there was a significant difference in the therapeutic efficacy of aPD-L1 before and after iRFA treatment. By calculating the relative tumor inhibition rate based on tumor weight, we found that the therapeutic efficiency of aPD-L1 in residual tumors after iRFA treatment was lower than that on untreated tumors (Figure 2C-D). These results suggest that iRFA treatment may alter the TIME and promote its phenotypic transformation to immunosuppression.

To validate this hypothesis, we investigated the distribution of F4/80^+^ TAMs and Ly6G^+^ neutrophils, which are typical representatives of immune cells with inhibitory phenotypes, in tumors before and after iRFA treatment. As shown in Figure 2E, iRFA treatment moderately enhanced the infiltration of TAMs and neutrophils into the tumor tissue. Treatment with aPD-L1 resulted in a slight reduction of the distribution of both cell types in untreated tumors, whereas this effect was reversed in iRFA-treated tumors. More TAMs and neutrophils infiltrated into the residual tumor tissues after the administration of aPD-L1 following iRFA. In addition, the dynamic changing of TIME after iRFA treatment was analyzed according to the flow chart in Figure 2F. Immunofluorescence imaging at different time points after iRFA showed that the infiltration of monocytes, TAMs and neutrophils continued to increase over time (Figure 2G). This observation was further confirmed using flow cytometry (Figure 2H, Figure S4). Notably, we examined the immune-activating subpopulation (CD80^+^) of F4/80^+^ TAMs over time and found that the percentage of these cells increased significantly on day 2 after iRFA treatment but decreased on day 9 (Figure S4). Transcriptomic analysis of tumor tissues before and after iRFA treatment consistently showed that a variety of pro-inflammatory genes including *Il1b*, *Il1r2*, *Tnfrsf8*, and *Ccl5*, and immune-activation related genes including *Isg15* and *Irf2bp1* as well as several immune-activation related pathways were significantly up-regulated after 2 days of iRFA treatment (Figure 2I and 2K, Figure S5-S6, Table S1). In contrary, the expression of anti-inflammatory genes including *Ly6a* and immunosuppressive genes including *Ctla4* and *Lag3* as well as several immunosuppressive pathways significantly increased after 9 days of iRFA treatment (Figure 2J and 2L, Figure S5-S6, Table S2). These results demonstrate that TIME experiences a transition from immune activation to suppression after iRFA treatment.

According to the detection of mRNA expression related to macrophage differentiation using qRT-PCR, we further found that genes related to M1 differentiation, including *Tnfa*, *Nos2*, *Nfkb* and *Stat1*, were significantly up-regulated 2 days after iRFA treatment (Figure 2M, Table S3). However, 9 days after iRFA treatment, the M1 phenotype was replaced by the M2 type, showing high expression of genes related to M2 differentiation, including *Il10*, *Arg1*, *Stat6*, and *Irf4* (Figure 2N, Table S3). These findings indicate that iRFA induces the transformation of TAMs from M1 to M2 during the TIME transition from immune activation to immunosuppression, which ultimately impairs the therapeutic efficacy of ICIs.

### Bisphosphonate-mineralized Nano-IFNγ hydrogel system can retain IFNγ in tumor tissues to exert long-term site-specific immunoregulation through a sustained-release effect

**Figure 3:**
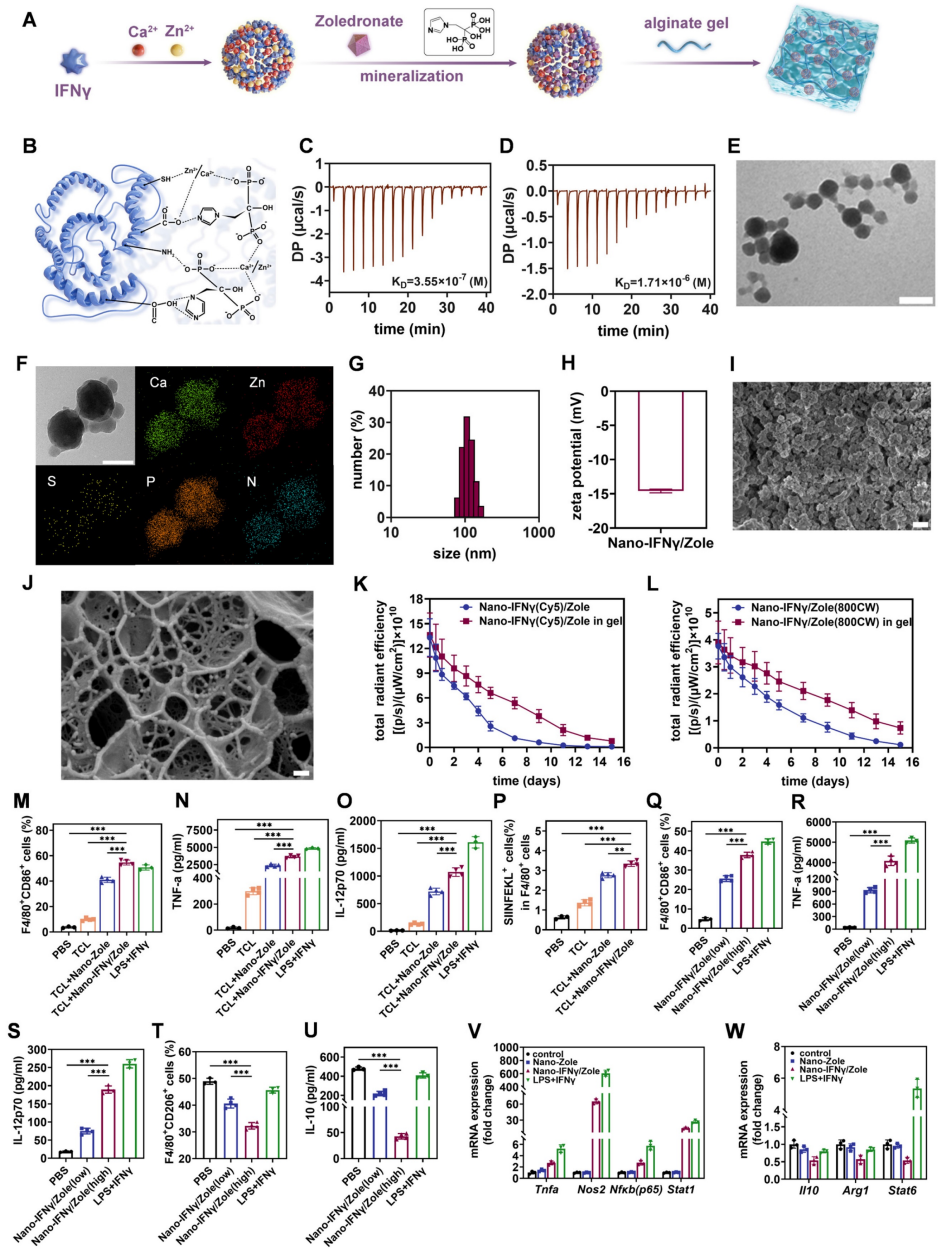
** Preparation, characterization, and *in vitro* immune-activation capacity of bisphosphonate-mineralized nano-IFNγ (Nano-IFNγ/Zole) hydrogel system.** (A) Schematic illustration of the preparation of Nano-IFNγ/Zole hydrogel system (B) Schematic illustration of the interaction between different components in Nano-IFNγ/Zole. (C and D) ITC profiles indicating the titration of zoledronate with IFNγ (C) and the titration of Ca^2+^/Zn^2+^ with IFNγ (D). (E) Representative TEM image of Nano-IFNγ/Zole, scale bar: 100 nm. (F) Element distribution of Nano-IFNγ/Zole measured by TEM, scale bar: 100 nm. (G) Particle size distribution of Nano-IFNγ/Zole measured by DLS. (H) Zeta potential of Nano-IFNγ/Zole measured by DLS. (I) Representative SEM image of Nano-IFNγ/Zole, scale bar: 100 nm. (J) Representative Cyro-SEM image of Nano-IFNγ/Zole in gel, scale bar: 100 nm. (K) Quantitative analysis of total radiant efficiency of IVIS images of necrotic tumor after Nano-IFNγ(Cy5)/Zole or Nano-IFNγ(Cy5)/Zole in gel administration (n = 3). (L) Quantitative analysis of total radiant efficiency of IVIS images of necrotic tumor after Nano-IFNγ/Zole(800CW) or Nano-IFNγ/Zole(800CW) in gel administration (n = 3). (M-O) Quantitative analysis of *in vitro* activation of BMDMs after different treatment, by detecting CD86 expression on F4/80^+^ BMDMs (M), and pro-inflammatory cytokines release of TNF-α (N) and IL-12p70 (O) (n = 3~4), TCL represents Tumor Cell Lysates. (P) Quantitative analysis of antigen cross-presentation ability of BMDMs after different treatment (n = 3~4), TCL represents Tumor Cell Lysates. (Q-U) Quantitative analysis of *in vitro* trans-differentiation of M2-BMDMs after different treatment, by detecting CD86 (Q) and CD206 (T) expression on F4/80^+^ BMDMs, and cytokines release of TNF-α (R), IL-12p70 (S) and IL-10 (U) (n = 3~4). (V and W) qRT-PCR analysis of M1-type genes (V) or M2-type genes (W) in M2-BMDMs after different treatment (n = 3). All statistical data are presented as mean ± SD; data were analyzed with two-tailed unpaired t tests; **, p < 0.01; ***, p < 0.001.

To overcome the negative impact of the suppressive TIME on immunotherapy in residual tumors after iRFA, we prepared a bisphosphonate-mineralized nano-IFNγ (Nano-IFNγ/Zole) hydrogel delivery system for local injection (Figure 3A). The primary goal of this design was to achieve long-term release of IFNγ in tumor tissues through protein granulation and gel encapsulation, thereby fulfilling the continuous reversal of the suppressive TIME. First, zinc/calcium ions induced the intermolecular aggregation of proteins through salting-out effects and direct coupling with sulfydryl groups in cysteine residues (Figure 3B). Furthermore, the coordination effect between phosphate groups on bisphosphonates and zinc/calcium ions, as well as the hydrogen bonding between N-containing groups (amino or imidazole groups) on bisphosphonates and amino acid residues in proteins, which had been validated by isothermal titration calorimetry (ITC) (Figure 3C-D), contributed to the binding of bisphosphonates to the surface of protein aggregates, and the size of aggregates remained stable at the nanometer scale. The interactions among IFNγ, bisphosphonate, and zinc/calcium ions led to the formation of a Nano-IFNγ/Zole triune nanostructure (Figure 3E-G). Transmission electron microscopy (TEM) images showed that the average particle size of a single mineralized IFNγ nanoparticle was around 40 nm (Figure 3E). Corresponding energy spectrum analysis showed that the nanoparticles were rich in zinc, calcium, phosphorus (rich in bisphosphonates) and sulphur (rich in IFNγ), confirming the characteristics of the trinity structure (Figure 3F). Nano-IFNγ/Zole possessed a hydrodynamic average particle size of 105.7 nm (Figure 3G) and exhibited electronegative surface characteristics, indicating that a small number of nanoparticles accumulated in the medium (Figure 3H). Scanning electron microscope (SEM) images showed that high concentrations of mineralized nano-IFNγ formed loose porous aggregation structures (Figure 3I). Enzyme-linked immunosorbent assay (ELISA) and high-performance liquid chromatography (HPLC) were performed to determine the loading efficiency of IFNγ and zoledronate, respectively. As shown in Figure S7, the loading efficiency of IFNγ was above 90% at the adding amount of 1 μg. 5 μg or 10 μg, owing to its interaction with both metal ions and zoledronate. And the loading efficiency of zoledronate in Nano-IFNγ/Zole was 93.94 ± 0.98%, nearly 10% higher than that in Nano-Zole (84.47% ± 0.54%) (Figure S8). Circular dichroism spectrum analysis and thermogravimetric analysis further indicated the successful synthesis of Nano-IFNγ/Zole (Figure S9).

When Nano-IFNγ/Zole was dispersed in alginate hydrogel, nanoparticles were stably embedded in the gel structure owing to the interaction between zinc/calcium ions on the particle surface and the carboxyl group in alginate (Figure 3J, Figure S10-S11). The addition of mineralized nano-IFNγ did not affect the rheological properties of the hydrogel (Figure S12). The release curve shown in Figure S13 showed that the nano-scaled assembly of IFNγ and the coating of the gel significantly delayed its release in the neutral medium. The percentage of IFNγ released from the gel system was less than 10% within 20 days. It is worth noting that the acidic environment significantly accelerates the release of IFNγ owing the destruction of coordination and electrostatic interactions. Results of cell experiments revealed that once the Nano-IFNγ/Zole is internalized by TAMs, IFNγ can be released under the acidic environment in endosomes, be secreted to the extracellular fluid and be able to exert its effect (Figure S14-S15).

In addition, we investigated the sustained release effect of the Nano-IFNγ/Zole-gel system *in vivo*. Nano-IFNγ(Cy5)/Zole was prepared and dispersed in alginate gel and injected into necrotic tumors. When IVIS imaging of tumors was performed after 2 weeks, a favorable amount of Cy5-labeled IFNγ was detected in the tumor tissue (Figure 3K, Figure S16-S17). These findings demonstrate that the bisphosphonate mineralized Nano-IFNγ/Zole-gel system can retain IFNγ in the tumor tissue to exert long-lasting and site-specific effects. Next, we conducted a metabolic experiment of Nano-IFNγ/Zole-gel system *in vivo* using HPLC, with intravenously-injected free zoledronate as positive control. Results revealed that more than 50% dose of the intravenously-injected free zoledronate was absorbed into femurs and tibias of mice within 10 minutes, while zoledronate in the plasma was almost undetectable (Figure S18B, Table S4). Meanwhile, merely ~10% dose of the zoledronate in the intratumorally-injected Nano-IFNγ/Zole in gel was absorbed into femurs and tibias in the first 0.5 h due to the burst release of Nano-IFNγ/Zole, and the percentage basically remained unchanged within the period of 168 hours, whereas zoledronate in the plasma of the Nano-IFNγ/Zole in gel-treated mice was also untraceable (Figure S18C, Table S5). Additionally, the long-term retention profile of zoledronate (labeled with 800CW) was also investigated *iv vivo* using IVIS imaging. Results of IVIS imaging indicated that compared to Nano-IFNγ/Zole in solution, preferable amount of zoledronate in the Nano-IFNγ/Zole in gel could retain in tumor lesions for more than 15 days, although a little amount of released zoledronate distributed to bones due to its bone-targeting property (Figure 3L, Figure S19).

### IFNγ-unloaded bisphosphonate-mineralized nanoparticles can independently inhibit tumor proliferation by reshaping TIME

Nitrogen-containing bisphosphonates, known as inhibitors of the mevalonate metabolic pathway, have been reported to be capable of activating the innate immune responses 14-16. Our previous works also demonstrated that bisphosphonates could enhance antigen presentation by forming nanoparticles with calcium ions and acting as adjuvants to activate dendritic cells 11 ,17 ,18. To compare the immune activation effects of different nitrogen-containing bisphosphonates, we prepared IFNγ-unloaded nanoparticles mineralized with alendronate, pamidronate, and zoledronate (Nano-Alen, Nano-Pami, and Nano-Zole, respectively). All the three types of nanoparticles exhibited similar particle sizes, morphology, and pH sensitivity (Figure S20-S22), but had different surface potentials owing to the differences in the chemical structures of bisphosphonate (Figure S23). All three nanoparticles were co-incubated with bone marrow-derived dendritic cells (BMDCs) to evaluate their immune activation capacity by measuring the up-regulation of CD80, CD86, and MHC II. As shown in Figure S24-S27, three nanoparticles activated BMDCs differently, with the Nano-Zole exhibiting the highest activation capacity. In addition, *in vivo* efficacy of the three bisphosphonate-mineralized nanoparticles was compared. According to the flow chart shown in Figure S28, 12 days after tumor inoculation, the three types of nanoparticles were dispersed in the alginate hydrogel and injected into the tumor tissue during the final stage of RFA surgery. Tumor growth curve, tumor inhibition rate and survival rate analysis showed that the addition of the three bisphosphonates significantly inhibited tumor recurrence after iRFA (Figure S28). Consistent with the results of the *in vitro* experiments, the Nano-Zole group showed the most potent anti-tumor effects. Subsequently, the TIME of the tumor tissues was evaluated using flow cytometry (Figure S29-S30). As shown in Figure S30A, Nano-Zole significantly reduced the proportion of M2-type macrophages upregulated by iRFA. Notably, although the infiltration of M1-type macrophages induced by the addition of Nano-Zole was lower than that induced by iRFA alone, the ratio of M1-type to M2-type macrophages in the TIME was significantly increased by the combination of Nano-Zole and iRFA. Nano-Zole treatment also caused an increase in the percentage of matured DCs (Figure S30B), an increase of cytotoxic CD8^+^T cells and a decrease in immunosuppressive T_reg_ cells in the tumor tissues (Figure S30C). These results suggest that IFNγ-unloaded bisphosphonate-mineralized nanoparticles can independently inhibit tumor proliferation by reshaping the TIME. Considering that Nano-Zole exhibited the strongest immune-activation and tumor-inhibition effects in different bisphosphonate-mineralized nanoparticles, subsequent studies were performed using a zoledronate-mineralized nano-IFNγ gel system to reshape TAMs after iRFA treatment.

### Zoledronate mineralized nano-IFNγ enhanced antigen presentation and immune activation of TAMs by inducing its trans-differentiation from M2 to M1 type

After confirming the independent immune activation effect of bisphosphonates, we investigated their synergistic effect with IFNγ by preparing zoledronate-mineralized nano-IFNγ (Nano-IFNγ/Zole). To simulate the *in vivo* combination of RFA and bisphosphonate-mineralized nanoparticles *in vitro*, we added tumor cell lysate to the culture medium of bone marrow-derived macrophages (BMDMs), because RFA treatment causes tumor cell necrosis and induces release of cell contents. As shown in Figure 3M and Figure S31, Nano-IFNγ/Zole significantly stimulated the expression of CD86 in BMDM cells. This treatment also induced the production of the pro-inflammatory cytokines TNF-α and IL-12p70 (Figure 3N-3O). Moreover, BMDMs exhibited the strongest antigen cross-presentation ability during co-incubation with tumor cell lysates and Nano-IFNγ/Zole, which was reflected in the up-regulation of MHC-I molecules loaded with tumor-specific antigen epitope peptides(SIINFEKL) (Figure 3P, Figure S31). By co-incubating different concentrations of Nano-IFNγ/Zole with M2-BMDMs, it can be seen from Figure 3Q-S and Figure S32 that this immune activation effect was significantly increased concentration-dependently. Interestingly, with an increase in the concentration of Nano-IFNγ/Zole, the proportion of M2 macrophages (CD206^+^) (Figure 3T, Figure S32) and the level of IL-10 secreted by M2 macrophages also decreased (Figure 3U). More importantly, Nano-IFNγ/Zole treatment significantly up-regulated the M1-type macrophage-related genes, while downregulating the expression of genes associated with the M2 macrophages (Figure 3V-W, Table S3). These phenomena were further validated in a RAW264.7 cell model that was inert to induced differentiation (Figure S33). Next, we compared the synergistic effects of IFNγ and Zole without mineralization *in vitro*. Flow cytometry and ELISA results indicated that neither IFNγ nor Zole possessed strong capability of inducing the repolarization of BMDM cells from M2 to M1, while the combination of IFNγ and Zole showed this repolarizing effect, even though it was significantly lower than that of Nano-IFNγ/Zole (Figure S34). Taken together, these results suggest that zoledronate-mineralized Nano-IFNγ most significantly enhances the differentiation of macrophages to the M1-phenotype and weakens their propensity to remain in the M2 phenotype.

**Figure 4:**
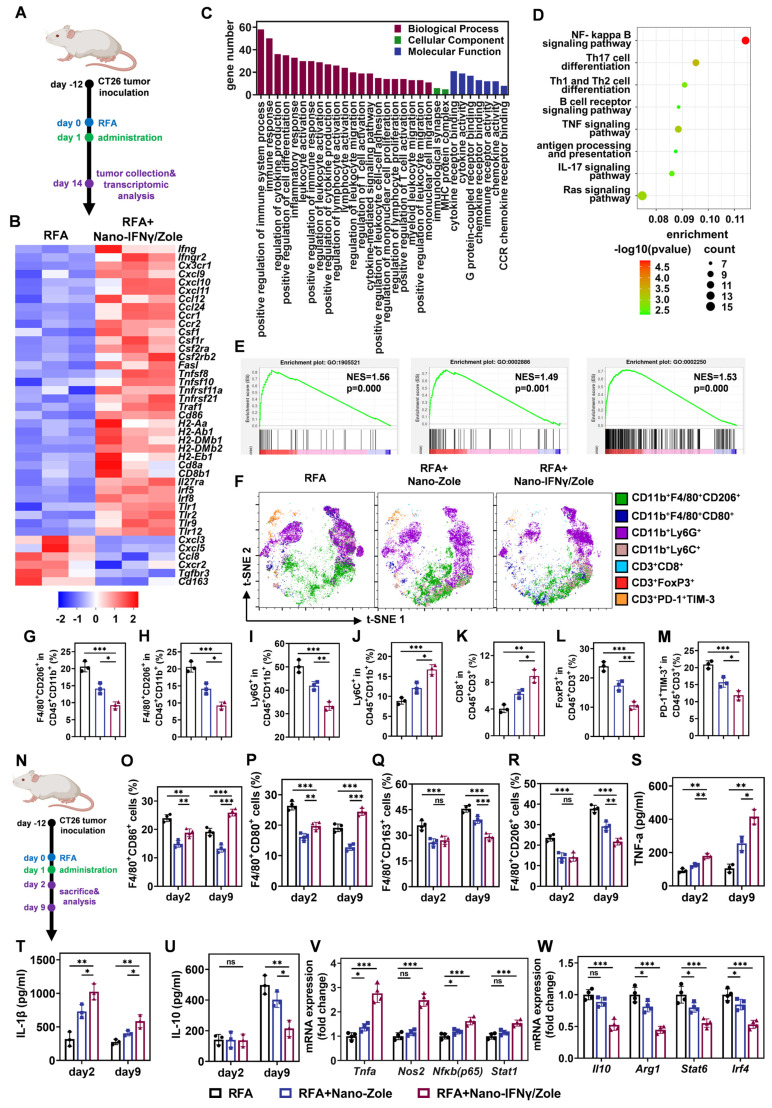
** Nano-IFNγ/Zole induced trans-differentiation of TAMs from M2 to M1 type after iRFA.** (A) Schematic illustration of the transcriptomic analysis of tumors after iRFA and Nano-IFNγ/Zole treatment. (B) Heatmap of DEGs associated with tumor progression and immune regulation after indicated treatment (n = 3). (C-E) GO analysis (C), KEGG analysis (D), and GSEA plot (E) of DEGs after indicated treatment (n = 3). (F) Representative t-SNE plots of tumor-infiltrating immune cell subpopulation 12 days after indicated treatment, using full spectrum FCM. (G-M) Percentage of tumor-infiltrating M2-TAMs(G), M1-TAMs(H), Ly6G^+^ MDSCs (I), Ly6C^+^ MDSCs (J), CD8^+^ T cells (K), T_reg_ cells (L) and PD-1^+^TIM-3^+^ exhausted T cells (M) after indicated treatment (n = 3). (N) Schematic illustration of the dynamic analysis of TAMs differentiation after iRFA and Nano-IFNγ/Zole treatment. (O-R) Percentage of tumor-infiltrating CD86^+^/CD80^+^ M1-TAMs (O and P) and CD163^+^/CD206^+^ M2-TAMs (Q and R) in different groups 2 days or 9 days after indicated treatment (n = 4). (R-T) Tumor-infiltrating cytokines IL-1β (T), TNF-α (U), and IL-10 (V) 2 days or 9 days after indicated treatment (n = 3). (V and W) qRT-PCR analysis of M1-type genes (V) or M2-type genes (W) expressed in sorted TAMs from tumor tissue after indicated treatment (n = 4). All statistical data are presented as mean ± SD; data were analyzed with two-tailed unpaired t tests; ns, no significance; *, p < 0.05; **, p < 0.01; ***, p < 0.001.

The remodeling ability of Nano-IFNγ/Zole on TAMs was further investigated *in vivo*. Tumor-bearing mice were treated with iRFA and Nano-IFNγ/Zole gel system (Figure 4A). Transcriptomic analysis of the residual tumor tissues showed that Nano-IFNγ/Zole significantly induced changes in the overall gene expression in tumor tissue after iRFA treatment (Figure S35). Multiple genes associated with innate immune activation, including *Tlr9*, *Tlr12*, *CD86*, and *Cxcl10* and genes involved in antigen presentation, including *H2-Aa*, *H2-Ab1*, and *H2-Eb1*, were significantly up-regulated after Nano-IFNγ/Zole treatment compared with those iRFA alone-treated samples (Figure 4B, Table S6). Genes that regulate cytokine TNF, including *Tnfsf8*, *Tnfsf10* and *Traf1*, were also significantly upregulated by Nano-IFNγ/Zole treatment (Figure 4B, Table S6). At the same time, the expression of related genes involved in immune tolerance, regulatory T cell activation and anti-inflammatory factor secretion, such as *Cxcl3*, *Cxcr2*,* Tgfbr3*, *Cd163*, was significantly decreased by Nano-IFNγ/Zole treatment (Figure 4B, Table S6). These findings were further validated by Gene Ontology (GO) and Kyoto Enyclopedia of Genes and Genomes (KEGG) analysis (Figure 4C-D). Notably, gene set enrichment analysis (GSEA) results showed that pathways regulating myeloid leukocyte-mediated immune response, adaptive immune response, and macrophage migration, were significantly upregulated after Nano-IFNγ/Zole treatment (Figure 4E). Above evidence demonstrates that the combination of zoledronate and IFNγ in the nanoparticulated combination activates the immune response as well as induces the differentiation and remodeling of myeloid cells. To further validate this finding, a subpopulation analysis of tumor infiltrated immune cells based on full-spectrum flow cytometry was performed. As shown in Figure 4F-M, iRFA treatment and its combination with Nano-IFNγ/Zole significantly changed the distribution and proportion of various immune cell subgroups in tumor tissues. Compared to the iRFA-treated group, the addition of Nano-IFNγ/Zole altered the tumor immune microenvironment. The proportion of M2-type macrophages decreased (Figure 4G) while more M1 macrophages were distributed in tumor tissues treated with Nano-IFNγ/Zole (Figure 4H). Notably, the number of Ly6G^+^ myeloid cells with immunosuppressive function also decreased after the addition of Nano-IFNγ/Zole (Figure 4I), while that of Ly6C^+^ myeloid cells with stronger antigen-presenting function increased (Figure 4J). Moreover, the proportion of CD8^+^ T lymphocytes increased while regulatory T lymphocytes and exhausted T lymphocytes decreased after Nano-IFNγ/Zole treatment (Figure 4K-M). These results suggested that Nano-IFNγ/Zole not only induced myeloid cell differentiation, but also redirected the function of T lymphocytes in the residual TIME after iRFA treatment.

Next, we investigated the dynamic changes of TAMs from the M2 to M1 type after iRFA treatment (Figure 4N, Figure S36-S37). The percentage of CD86^+^ and CD80^+^ M1 macrophages gradually decreased over time after iRFA (Figure 4O-P). In contrast, more CD163^+^ and CD206^+^ M2 macrophages were recruited and distributed continuously in the residual tumor tissue and the addition of Nano-IFNγ/Zole significantly reversed this tendency (Figure 4Q-R). The proportion of CD86^+^ and CD80^+^M1-type macrophages increased significantly after 9 days compared with the results 2 days after iRFA treatment (Figure 4O-P). Infiltration of M2-type macrophages was also significantly delayed after treatment with Nano-IFNγ/Zole (Figure 4Q-R). Intratumoral cytokine detection showed that TNF-α and IL-1β were continuously produced after the addition of Nano-IFNγ/Zole (Figure 4S-T), while IL-10 secretion was significantly inhibited (Figure 4U). The expression levels of differentiation-associated genes in the TAMs isolated from the tumor tissues were further detected using qRT-PCR after 12 days of iRFA treatment (Figure S38, Table S3). As shown in Figure 4V-W, M1-related genes, including *Tnfa*, *Nos2*, *Nfkb* and *Stat1*, were significantly up-regulated, whereas the expression of M2-related genes, such as *Il10*, *Arg1*, *Stat6* and *Irf4*, was significantly decreased. These results convincingly indicate that Nano-IFNγ/Zole can reshape the of residual tumors after iRFA treatment by inducing the trans-differentiation of TAMs from the M2 to M1 type and enhance the immune response of immune cells.

### Mechanism of TAM trans-differentiation induced by Nano-IFNγ/Zole relies on the synergistic effect of IFNγ and zoledronate on mevalonate metabolism inhibition

**Figure 5:**
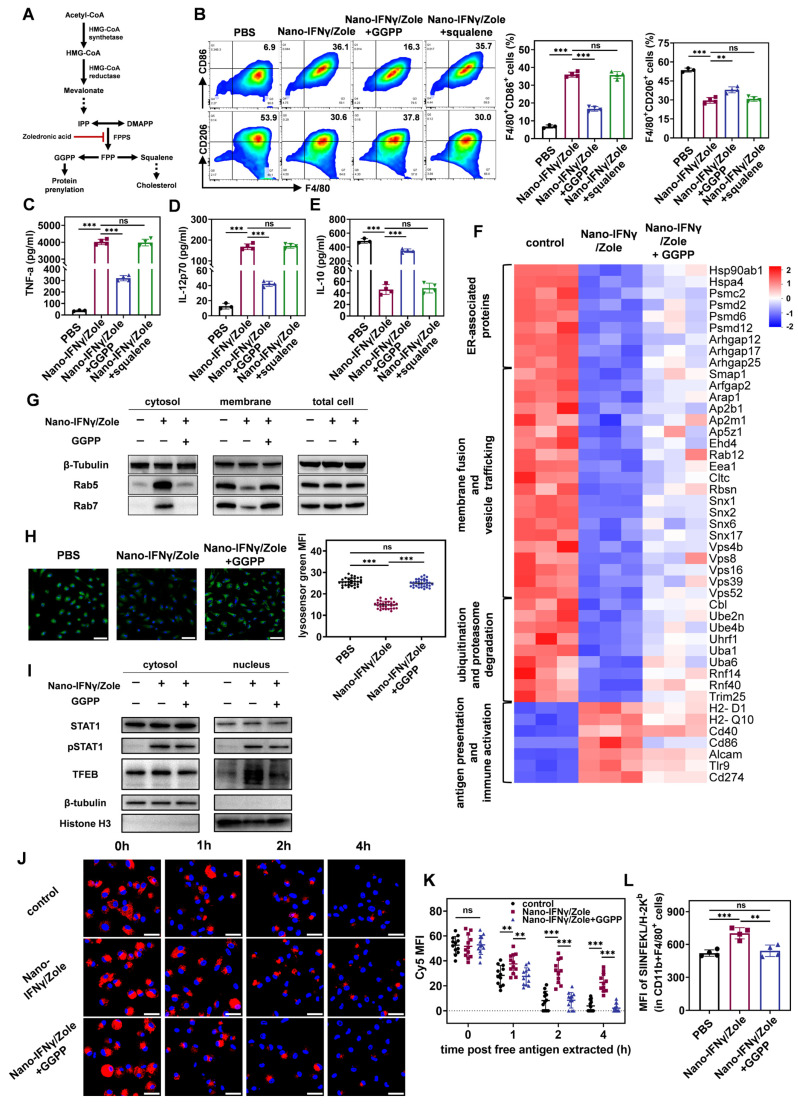
** TAM trans-differentiation induced by Nano-IFNγ/Zole relies on the synergistic effect of IFNγ and zoledronate on mevalonate metabolism inhibition.** (A) Schematic illustration of the mevalonate pathway and the mechanism of bisphosphonates in this pathway. (B-E) Mechanism study of BMDM trans-differentiation, by detecting the expression of CD86 and CD206 on F4/80^+^ BMDMs (B), and the release of cytokines TNF-α (C), IL-12p70 (D) and IL-10 (E) by BMDMs (n = 3~4). (F) Heat map for protein expressing profile of M2-BMDMs after indicated treatment (n = 3). (G) Expression of Rab5 and Rab7 in M2-BMDMs after indicated treatment. (H) Representative fluorescence images and corresponding quantification of lysosome acidification of M2-BMDMs after indicated treatment (n = 30~31 cells), scale bar: 50 μm. (I) Expression of STAT1, pSTAT1 and TFEB in M2-BMDMs after indicated treatment. (J and K) Representative fluorescence images (J) and corresponding quantification (K) of Cy5-labeled OVA retained in M2-BMDMs after indicated treatment (n = 12 cells), scale bar: 50 μm. (L) FCM analysis of antigen cross-presentation ability of M2-BMDMs after indicated treatment (n = 4). All statistical data are presented as mean ± SD; data were analyzed with two-tailed unpaired t tests; ns, no significance; *, p < 0.05; **, p < 0.01; ***, p < 0.001.

To investigate the mechanism of trans-differentiation of TAMs induced by Nano-IFNγ/Zole, we investigated the effect of zoledronate on BMDMs and its synergistic effect with IFNγ. As shown in Figure 5A, zoledronate inhibits farnesyl pyrophosphate synthetase (FPPS) in the mevalonate metabolic pathway. Blocking this synthesis reaction results in a decrease in the level of the downstream metabolite farnesyl pyrophosphate (FPP), leading to a deficiency of squalene, a key intermediate in cholesterol biosynthesis, and ultimately a decrease in cholesterol synthesis 15. In addition, a deficiency in FPP results in a decrease in the level of geranylgeranyl pyrophosphate (GGPP), a key precursor molecule for the isoprene modification of proteins and ubiquinone 15 ,19. To identify which specific pathway of mevalonate metabolism is involved in the trans-differentiation of TAMs, we added metabolic intermediates in different pathways to restore metabolism during the co-incubation of Nano-IFNγ/Zole and BMDMs. As shown in Figure 5B, the addition of GGPP significantly inhibited the up-regulation of CD86, a co-stimulatory molecule in BMDMs, induced by Nano-IFNγ/Zole. At the same time, the Nano-IFNγ/Zole-induced reduction of the percentage of CD206^+^ M2 macrophages was also slightly restored by GGPP supplementation (Figure 5B). However, squalene did not show any effect on the efficacy of Nano-IFNγ/Zole in both types of macrophages (Figure 5B). In addition, GGPP inhibited the Nano-IFNγ/Zole-induced secretion of pro-inflammatory cytokines TNF-α and IL-12p70, while restoring the production of the anti-inflammatory cytokine IL-10 (Figure 5C-5E). These findings suggest that the protein isoprene modification pathway in mevalonate metabolism, rather than the cholesterol synthesis pathway, regulates TAM trans-differentiation.

Next, to understand how isoprene modification regulates the function of macrophages, the protein expression changes of macrophages after Nano-IFNγ/Zole treatment and its combination with GGPP treatment were studied using a liquid chromatography tandem mass spectrometry (LC-MS/MS)-based proteomics method. Heat maps based on cluster analysis showed that GGPP supplementation restored the changes in the protein expression profile induced by Nano-IFNγ/Zole to levels similar to those in the control group (Figure 5F). Notably, we found that Nano-IFNγ/Zole significantly reduced the expression of several small GTPases and aptamers in macrophages, including proteins of the Rab, Arf, Vps, and Snx families, which are involved in membrane fusion and vesicle transport 20-23 (Figure 5F). A variety of proteins related to ubiquitination and proteasome degradation, including Cblb, Ube2n, and Uba1 24 ,25, was also down-regulated by Nano-IFNγ/Zole. The addition of GGPP suppressed these effects. It suggests that Nano-IFNγ/Zole may induce trans-differentiation of macrophages by delaying the intracellular vesicle transport.

Previous studies have shown significant differences in vesicle transport between M1 and M2 macrophages 26 ,27. The M2 macrophages have stronger endocytosis and a lower pH lysosomal environment to facilitate the complete digestion of contents and nutrient absorption 27 ,28. In contrast, mild lysosomal acidification of M1-type macrophages results in only partial degradation of internalized xenobiotics, which facilitates the presentation of antigenic peptides and activation of downstream signals from pattern recognition receptors 27 ,28. Thus, the regulation of lysosomal acidification levels may induce phenotypic differentiation in macrophages 27 ,29. To uncover whether the efficacy of Nano-IFNγ/Zole is based on a similar mechanism, we examined the isoprene modification levels of Rab5 and Rab7, two key proteins of the Rab family involved in vesicular transport. Only isoprene-modified Rab proteins can anchor to the endosomal membrane and regulate inter-vesicular fusion and transport 19 ,20. Rab5 regulates the transport of initially internalized vesicles to early endosomes, whereas Rab7 participates in the transport of early endosomes to late endosomes and lysosomes 20 ,21. By separating the cytoplasmic and membrane structures of BMDM cells and conducting immunoblot analysis, we showed in Figure 5G that Nano-IFNγ/Zole treatment resulted in the separation of a substantial amount of Rab5 and Rab7 proteins from the membrane structures and their distribution in the cytoplasm, while the addition of GGPP relocated Rab5 and Rab7 proteins to the membrane structures. It shows that Nano-IFNγ/Zole blocks the isoprene modification of Rab proteins by inhibiting GGPP production in the mevalonate metabolic pathway, subsequently affecting the vesicle transport and lysosome acidification levels. As shown in the confocal microscopy images in Figure 5H, the fluorescence of lysosomes stained by the pH-responsive lysosensor probe was significantly attenuated after treatment with Nano-IFNγ/Zole and restored by GGPP supplementation. This phenomenon was confirmed by the quantification of corresponding fluorescence intensity, indicating that the lysosome acidification level was significantly weakened by Nano-IFNγ/Zole.

Decreased lysosomal acidification is generally followed by the inhibition of mTORC1 function and activation of transcription factor EB (TFEB) 30 ,31. Using immunoblot analysis, we found that the Nano-IFNγ/Zole treatment led to the translocation of the TFEB from the cytoplasm to the nucleus (Figure 5I). It showed that Nano-IFNγ/Zole activated TFEB signaling by blocking the vesicular transport and lysosomal acidification in macrophages. Several genes related to innate immune response, including *Il1b* and *Tnfa*, are regulated by TFEB 32. Besides, Nano-IFNγ/Zole facilitated phosphorylation and nuclear translocation of STAT1 in BMDMs in a mevalonate pathway-independent manner (Figure 5I), and IFNγ/STAT1 signaling pathway has been reported to mediate the activation of downstream immune-related pathways, such as PI3K-Akt pathway and NF-κB pathway 33. Therefore, the synergy between the TFEB signal promoted by zoledronate and the JAK/STAT1 pathway activated by IFNγ/IFNγ receptor interaction may play a major role in Nano-IFNγ/Zole-mediated TAM trans-differentiation to the M1 type.

Furthermore, we investigated other effects of mevalonate metabolism inhibition on macrophages induced by Nano-IFNγ/Zole treatment. As vesicle transport is inhibited, the degradation of internalized exogenous substances is also affected 28. By using fluorescently labelled ovalbumin and detecting its dynamic intracellular degradation process, we found that, unlike in the control group, some ovalbumin was still retained after 4 h of Nano-IFNγ/Zole treatment (Figure 5J), indicating that the intracellular degradation of ovalbumin was delayed. Quantitative analysis based on confocal imaging and flow cytometry further validated this observation (Figure 5K, Figure S39). Additionally, GGPP supplementation restored the degradation of exogenous substances to a level consistent with that of the control group. It suggests that the inhibitory effect of Nano-IFNγ/Zole on antigen degradation is due to the loss of protein isoprene modification caused by the blocking of mevalonate metabolism. Delayed lysosomal degradation often facilitates the presentation of antigenic peptides 29. In line with this, Nano-IFNγ/Zole treatment resulted in increased presentation of tumor-specific antigenic peptides to the membrane surface when the OVA was co-incubated with BMDMs (Figure 5L). In contrast, GGPP weakened this antigen-presenting synergistic effect by restoring mevalonate metabolism. These results suggest that Nano-IFNγ/Zole also contributes to enhancing the immune response of macrophages through the delayed degradation of antigens induced by the inhibition of mevalonate metabolism.

To conclude, these findings elucidate that the mechanism of Nano-IFNγ/Zole-induced trans-differentiation of TAMs is due to the synergistic effect of IFNγ and mevalonate metabolism. As shown in Figure 1, blocking of mevalonate metabolic pathway resulted in the loss of the downstream intermediate metabolite GGPP, which prevented a variety of small GTP proteins, including Rab5 and Rab7, from being anchored to the endosomal membrane through isoprene modification. Dysregulated Rab proteins inhibited inter-vesicular fusion and transport and reduced lysosomal acidification. The subsequent activated transcription factor TFEB synergized with the IFNγ-activated transcription factor STAT1 to promote the trans-differentiation of TAMs. The inhibition of lysosomal acidification in macrophages also delayed the degradation of antigens, enhanced the presentation of antigenic peptides, and promoted the activation and immune response of M1 macrophages.

### Nano-IFNγ/Zole inhibits the recurrence and metastasis of CRCLM after iRFA by remodeling the suppressive TIME

**Figure 6:**
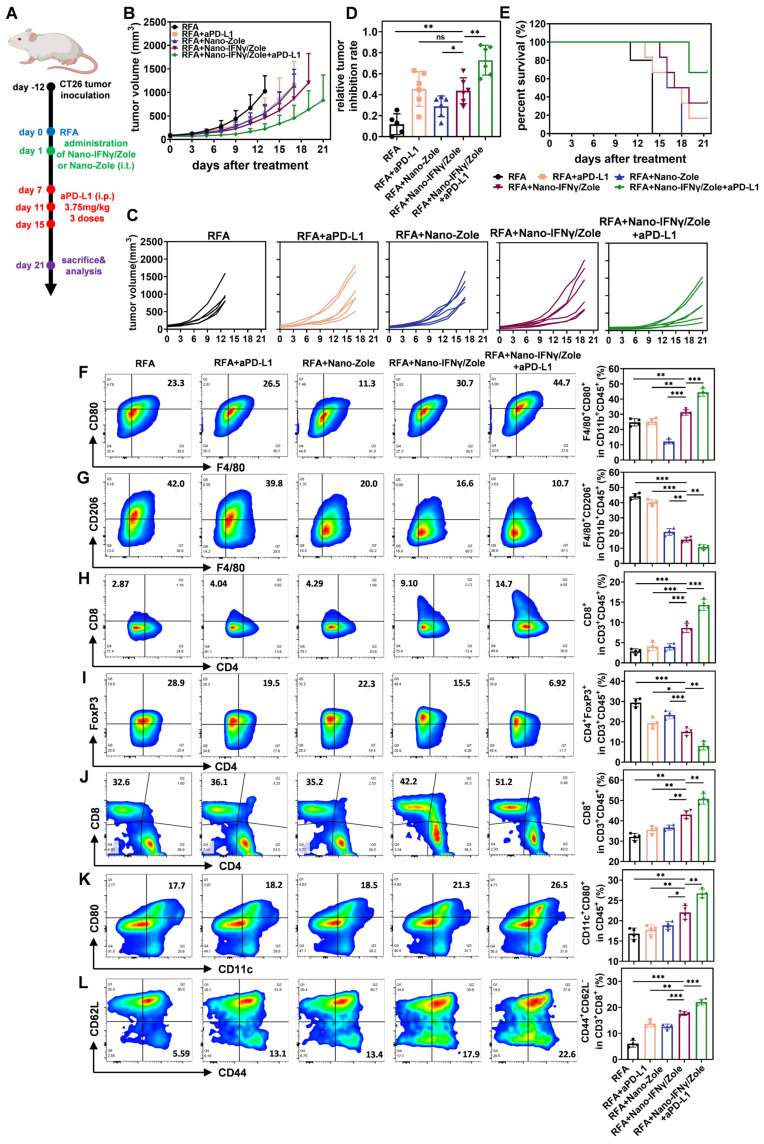
** Nano-IFNγ/Zole inhibits the recurrence of subcutaneous CRC after iRFA by reshaping the suppressive TIME.** (A) Schematic illustration of the assessment of the *in vivo* anti-tumor efficacy of Nano-IFNγ/Zole and its synergistic effect with PD-L1 antibody; i.t., intratumoral injection; i.p. intraperitoneal injection. (B and C) Growth curves of tumor volume after different treatment (n = 5~6). (D) Relative tumor inhibition rate based on tumor weight (n = 5~6). (E) Survival curves of all experiment groups (n=5~6). (F-I) Representative FCM plots and corresponding quantification of tumor-infiltrating M1-TAMs (F), M2-TAMs (G), CD8^+^ T cells (H) and T_reg_ cells (I) after different treatment (n = 4). (J-L) Representative FCM plots and corresponding quantification of spleen-infiltrating CD8^+^ T cells (J), matured DC cells (K), and effector memory T cells (L) after different treatment (n = 4). All statistical data are presented as mean ± SD; data were analyzed with two-tailed unpaired t tests; ns, no significance; *, p < 0.05; **, p < 0.01; ***, p < 0.001.

Colorectal cancer is one of the most common malignancies worldwide, ranking third and fourth in incidence and mortality, respectively 34. Liver metastasis of colorectal cancer is one of the main factors leading to poor prognosis and even death after surgical resection 35. RFA has been widely used for the local treatment of colorectal cancer liver metastasis (CRCLM) 3 ,36. However, owing to the diversity and complexity of tumor metastasis, some CRCLM may undergo iRFA, resulting in a suppressive TIME that may be resistant to subsequent ICI treatment 5. Therefore, after the elucidation of the mechanism by which Nano-IFNγ/Zole induces TAM trans-differentiation, we investigated the *in vivo* therapeutic efficacy of this delivery system on residual tumors after iRFA. CT26 cell line was used to generate a tumor-bearing mouse model. As shown in Figure 6A, tumor tissues with a certain burden were first treated with iRFA and then injected with hydrogel containing Nano-IFNγ/Zole. To evaluate the effect of the addition of Nano-IFNγ/Zole on the efficacy of ICIs, aPD-L1 was administered 3 times from day 7 after iRFA. Tumor growth curves (Figure 6B-C) clearly showed that the administration of Nano-IFNγ/Zole after iRFA delayed residual tumor proliferation compared to iRFA treatment alone. Notably, the combination of Nano-IFNγ /Zole with the aPD-L1 most significantly inhibited tumor growth and prolonged overall survival in tumor-bearing mice (Figure 6B-E). To verify whether this synergistic effect was due to the remodeling of the TIME caused by Nano-IFNγ/Zole, different subpopulations of infiltrated immune cells in tumors were evaluated using flow cytometry. As shown in Figure 6F-G, Nano-IFNγ/Zole treatment significantly promoted the infiltration of CD80^+^ M1 macrophages, while decreasing the proportion of CD206^+^ M2 macrophages. Owing to this trans-differentiation effect of TAMs, adaptive immune response in tumors also underwent a corresponding shift from suppression to activation. More CD8^+^ cytotoxic T cells were recruited to the tumor (Figure 6H). In contrast, fewer immunosuppressive T_reg_ cells were distributed in the tumor after treatment with Nano-IFNγ/Zole (Figure 6I). These findings were also confirmed using tumor tissue section and staining analysis (Figure S40-S42), which demonstrated that Nano-IFNγ/Zole could inhibit local recurrence of colorectal cancer after iRFA treatment by remodeling the suppressive TIME. The addition of aPD-L1 further enhanced the Nano-IFNγ/Zole-induced immune response (Figure 6F-I). This effect may be resulted from IFNγ-induced up-regulation of PD-L1 expression on the surface of CT26 tumor cells (Figure S43-S44), which is also consistent with the previous reports 33 ,37. An increase in PD-L1 levels improves the targeting of monoclonal antibodies and enhances the recognition and killing effects of activated cytotoxic T cells on tumor cells 38 ,39. It expounds that Nano-IFNγ/Zole can achieve a synergistic effect with aPD-L1 by regulating PD-L1 expression. During the whole treatment period, the mouse body weight and blood test indicators were not affected by Nano-IFNγ/Zole and its combination with aPD-L1 (Figure S45-S49). Analysis of tissue sections based on hematoxylin and eosin (H&E) staining showed that major organs of mice were not damaged by the long-acting effect of Nano-IFNγ/Zole at the tumor site (Figure S50). Besides, we conducted a biosafety assay in order to evaluate the differences of the side effects of Nano-IFNγ/Zole in gel, free IFNγ in gel (i.t.) or free IFNγ (i.v.) *in vivo*. Mouse body weight monitoring was performed every other day, and serum and tumor tissue were collected for the detection of several proinflammatory cytokines 1 day, 2 days and 7 days after administration. Results in Figure S51 revealed that intravenous injection of free IFNγ caused obvious weight loss of mice in both short-term and long-term of observation, with the IFNγ intratumoral injection group following, while Nano-IFNγ/Zole in gel caused acceptable effects on mice body weight.

Furthermore, ELISA results in Figure S52 indicated that intravenous injection of free IFNγ led to a serious cytokine storm in mice, especially 1 day and 2 days after administration. On the contrary, owing to slow-release profile of Nano-IFNγ/Zole in gel, its intratumoral administration resulted in negligible effects on serum level of proinflammatory cytokines, whereas significantly higher amount of IFNγ, CXCL10, CCL2, IL-6, TNF-α and IL-12p70 were detected inside the tumor tissue, which was requisite for tumor recession. Moreover, H&E staining results of mouse liver and kidney at different time points showed that severe pathological changes, including congestion and edema of interstitial blood vessel, infiltration of lymphocytes and granulocytes as well as emergence of necrotic foci, were observed in the livers and kidneys of i.v.-injected mice, and this was also predominant 1 day and 2 days after administration (Figure S53). Nano-IFNγ/Zole in gel group, however, exhibited far less pathological changes, which was similar as iRFA-treated control group. Altogether, these results revealed that a therapeutic dose of Nano-IFNγ/Zole in gel intratumoral injection would not cause obvious side effects of mice, demonstrating the biosafety of Nano-IFNγ/Zole in combination with RFA.

Besides, non-mineralized IFNγ and Zole were then adopted for intratumoral injection to supplementarily investigate the anti-tumor efficacy of two free drugs and their physical mixture. Results in Figure S54 revealed that either IFNγ or Zole (dispersed in gel, respectively) could mildly restrain CT26 tumor growth after iRFA, and their combination (IFNγ and Zole dispersed in gel simultaneously) showed better anti-tumor effect. Not surprisingly, the anti-tumor effect of IFNγ+Zole group was significantly weaker than the Nano-IFNγ/Zole in gel-treated group. We assume the mild anti-tumor effect of non-mineralized IFNγ or Zole may be attributed to their immune regulation effect. However, rapid release of non-mineralized cargo led to the attenuation of the anti-tumor effect and thus failed to control tumor growth after iRFA in a long-term after administration.

**Figure 7:**
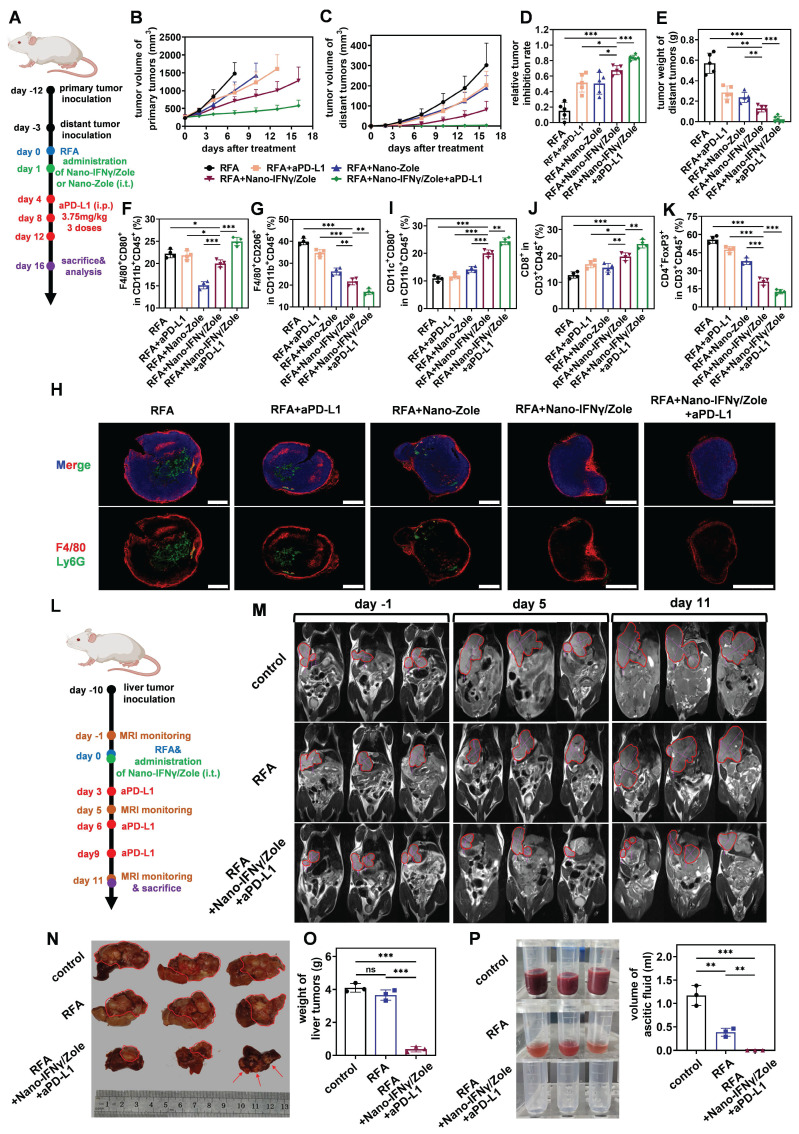
** Nano-IFNγ/Zole inhibits the metastasis of subcutaneous CRC and the recurrence of *in-situ* CRCLM after iRFA.** (A) Schematic illustration of the assessment of the distant anti-tumor efficacy of Nano-IFNγ/Zole and its synergistic effect with PD-L1 antibody; i.t., intratumoral injection; i.p. intraperitoneal injection. (B and C) Growth curves of tumor volume of primary tumors (B) and distant tumors (C) after different treatment (n = 5~6). (D) Relative tumor inhibition rate of primary tumors (n = 5~6). (E) Weight of the distant tumors (n = 5~6). (F and G) FCM analysis of tumor-infiltrating M1-TAMs (F), M2-TAMs (G) in distant tumors after different treatment (n = 4). (H) Immunofluorescence images of tumor-infiltrating macrophages and neutrophils in distant tumors, scale bar: 2 mm. (I to K) FCM analysis of tumor-infiltrating matured DC cells (I), CD8+ T cells (J) and T_reg_ cells (K) in distant tumors after different treatment (n = 4). (L) Schematic illustration of the assessment of *in-situ* anti-tumor efficacy of Nano- IFNγ/Zole plus PD-L1 antibody against CRCLM; i.t., intratumoral injection. PD-L1 antibody was administered intratumorally at 3.75 mg/kg. (M) T2-weighted MRI images of mice abdomen after different treatment. (n = 4). (N and O) Photograph (N) and corresponding weight of (O) liver tumors after different treatment (n = 3). (P) Photographs and corresponding volume of ascitic fluid collected from mice abdomen after different treatment (n = 3). All statistical data are presented as mean ± SD; data were analyzed with two-tailed unpaired t tests; ns, no significance; *, p < 0.05; **, p < 0.01; ***, p < 0.001.

In addition to the remodeling of TIME, the regulatory effect of Nano-IFNγ/Zole on systemic immunity was also investigated. Spleen immune cell subpopulation analysis showed that DC cells and effector memory T cells were significantly activated after the treatment with Nano-IFNγ/Zole (Figure 6J-L). The *in vitro* killing test of spleen cells further demonstrated that activated innate and adaptive immunity could effectively clear tumor cells (Figure S55). These findings implied that Nano-IFNγ/Zole was not only able to reshape the local tumor immune microenvironment but may also clear distal microlesions that RFA cannot treat by activating the systemic immune response. To test this hypothesis, we established a mouse model containing both the primary tumor and the distal lesion and designed a combination therapy regimen of Nano-IFNγ/Zole and iRFA according to the flowchart of Figure 7A. As shown in Figure 7B-E and Figure S56, administration of Nano-IFNγ/Zole after iRFA not only inhibited tumor recurrence *in situ*, but also limited the growth of distal tumors. Immune cell subpopulation analysis of the distal tumors showed that Nano-IFNγ/Zole also induced the trans-differentiation of TAMs from the M2 type to the M1 type (Figure 7F-G, Figure S57). Deep infiltration of tumor-associated macrophages and Ly6G^+^ myeloid suppressor cells in distal tumors was significantly reduced after treatment with Nano-IFNγ/Zole (Figure 7H). More matured DC cells and cytotoxic T cells were recruited to the distal lesion due to Nano-IFNγ/Zole treatment (Figure 7I-J, Figure S57-S58), while the distribution of regulatory T cells was significantly impaired (Figure 7K, Figure S56-S57). More importantly, the addition of aPD-L1 further enhanced the remodeling effect of Nano-IFNγ/Zole on the distant tumor immune microenvironment, and showed the strongest therapeutic efficacy against both primary and distant tumors with minimal influence on mice body weight (Figure 7A-K, Figure S56-S60). These findings suggest that Nano-IFNγ/Zole could overcome the inhibitory effect of iRFA on immune checkpoint blockade therapy and synergistically enhance the efficacy of immunotherapy through regulation of the TIME.

Finally, we investigated the synergistic effect of Nano-IFNγ/Zole administration on post-iRFA immunotherapy by constructing an *in-situ* CRCLM model in the liver (Figure 7L). CT26 tumor fragments were directly injected into the liver to induce liver metastasis *in situ*. When the liver metastases had grown to a certain size, the mice were anaesthetized and subjected to iRFA. Nano-IFNγ/Zole, which was dispersed in gel, was injected into the peripherally ablated tumor during the final stage of surgery.

Magnetic resonance imaging (MRI) was used to continuously monitor the recurrence of residual tumors in liver after iRFA. RFA alone limited residual tumor proliferation more gently than no treatment (Figure 7M-N). The administration of Nano-IFNγ/Zole and its combination with aPD-L1 were most effective in restraining the recurrence of residual tumors (Figure 7M-O). In addition, adverse reactions induced by sustained tumor proliferation in the liver, including ascites generation, were totally suppressed by the combination of Nano-IFNγ/Zole and aPD-L1 (Figure 7P). These findings confirmed the potential of Nano-IFNγ/Zole for synergistic immune checkpoint blockade therapy by reshaping the TIME. This provides a new adjuvant therapy option to overcome the therapeutic limitations of RFA due to tumor heterogeneity, which is superior to the current immune checkpoint inhibitors alone.

## Discussion

The development of RFA therapy is greatly limited by the anatomical complexity of tumor tissues and the heterogeneity of immune microenvironment 3. When RFA is performed on large, irregularly-shaped tumors or tumors adjacent to large blood vessels, the thermal killing effect triggered by the electrode often fails to completely cover all areas of the tumor, resulting in incomplete ablation 3 ,5. Residual tumor cells in these incompletely ablated areas proliferate, leading to tumor recurrence and distal metastasis 5 ,17. Incomplete thermal ablation can alter the local immune microenvironment 6. It has been reported that the accumulation of anti-inflammatory cytokines and the recruitment of neutrophils and myeloid suppressor cells impair the response of residual tumors to subsequent adjuvant therapy and accelerate disease progression 5. In this study, we confirmed the involvement of TAMs in the remodeling of the immune microenvironment after iRFA treatment and revealed the dynamic process of their transformation from M1-type to M2-type. In order to eliminate the negative effects of TAMs due to its contribution to residual tumor recurrence and metastasis, we designed and prepared bisphosphonate-mineralized nano-IFNγ and administered it in a single dose via intratumoral injection during RFA surgery. Nanoparticulated IFNγ could concentrate on the ablation site and play a long-term regulatory role in the tumor immune microenvironment after iRFA treatment through slow release, while avoiding toxic and side effects of IFNγ on surrounding normal tissues and the whole body. Zoledronate not only constructed the nano-IFNγ skeleton through coordination with calcium and zinc ions, but also directly participated in the immune regulation as an inhibitor of mevalonate metabolism. Zoledronate reduced lysosomal acidification by inhibiting isoprene modification of Rab family proteins, enhanced tumor antigen cross-presentation and activated the TFEB pathway. These outcomes, in conjunction with IFNγ-activated JAK/STAT1 signaling, promoted the trans-differentiation of tumor-associated macrophages from the M2 to M1 type, remodeled the suppressive tumor immune microenvironment created by iRFA, and restored therapeutic sensitivity to ICIs. Considering that zoledronate and IFNγ have already been approved for the clinical treatment of osteoporosis and internal rheumatoid arthritis, respectively, the combined delivery of these two components in a nanostructure will provide an effective strategy with great translational promise to overcome the poor prognosis after clinically incomplete RFA.

## Supplementary Material

Supplementary methods, figures and tables.

## Abbreviations

BMDCs: bone marrow-derived dendritic cells
BMDMs: bone marrow-derived macrophages
CRC: colorectal cancer
CRCLM: colorectal cancer liver metastasis
GM-CSF: granulocyte-macrophage colony stimulating factor
ICI: immune checkpoint inhibitor
IL-10: interleukin-10
IFNγ: interferon gamma
iNOS: inducible nitric oxide synthase
iRFA: incomplete radiofrequency ablation
M-CSF: macrophage colony-stimulating factor
PD-1: programmed cell death protein 1
PD-L1: programmed cell death ligand 1
RFA: radiofrequency ablation
qRT-PCR: quantitative reverse transcription-polymerase chain reaction
STAT1: signal transducer and activator of transcription 1
TAMs: tumor-associated macrophages
TFEB: transcription factor EB
TGF-β: transforming growth factor-beta
TIME: tumor immune microenvironment
TNF-α: tumor necrosis factor-alpha
