# Supplementary material for: Bisphosphonate-mineralized nano-IFNγ suppresses residual tumor growth caused by incomplete radiofrequency ablation through metabolically remodeling tumor-associated macrophages
Source: Theranostics. 2025 Jan 1;15(3):1057–76. doi: 10.7150/thno.100998 (PMC11700868; doi:10.7150/thno.100998)
Supplement: Supplementary file 1 — Supplementary methods, figures and tables. [file thnov15p1057s1.pdf]

Supplementary Materials

Bisphosphonate-mineralized nano-IFNγ suppresses residual tumor growth caused by incomplete radiofrequency ablation through metabolically remodeling tumor-associated macrophages

Zhicheng Yan [1,2#], **Bing Wang** [3#], Yuhan Shen [3], Junji Ren [1,2], Meifang Chen [1,2], Yunhui Jiang [1,2], Hao Wu [3], Wenbing Dai [1,2], Hua Zhang [1,2], Xueqing Wang [1,2], Qiang Zhang [1,2*], Wei Yang [3*], Bing He [1,2*]

Supplementary Methods

Materials

IGEPAL®CA-630 (Catalog No. I3021, MW~603) was obtained from Sigma-Aldrich (Missouri, USA). Cyclohexane, calcium chloride dihydrate and zinc chloride were supplied by Macklin (Shanghai, China). Recombinant murine IFNγ was obtained by Yeasen (Shanghai, China). Zoledronic acid monohydrate ($C_5H_{10}N_2O_7P_2 \cdot H_2O$) was purchased from Energy Chemical (Shanghai, China), sodium alendronate ($C_4H_{13}NO_7P_2$) was obtained from Aladdin (Shanghai, China), and pamidronic acid ($C_3H_{11}NO_7P_2$) was supplied by Meilunbio (Dalian, China). Sodium alginate was provided by J&K Chemical (Beijing, China). Lipopolysaccharide was obtained from Solarbio (Beijing, China). Recombinant murine M-CSF, GM-CSF and IL-4 were purchased from PeproTech (Rocky Hill, USA). Geranylgeranyl pyrophosphate ammonium salt (GGPP) was purchased from Glpbio (California, USA) and squalene was supplied by Tokyo Chemical Industry (Tokyo, Japan). In vivo anti-mouse PD-1 antibody (Catalog No. BE0146) and anti-mouse PD-L1 antibody (Catalog No. BE0101) was obtained from BioXell (New Hampshire, USA).

iRFA mouse model construction and treatment

A density of 5×10^5 CT26 cells were subcutaneously injected into female BALB/c mice (6–8 weeks) on the right flank. Twelve days after tumor inoculation, the tumor-bearing mice were randomly divided into four groups to evaluate the efficacy of immunotherapy with or without RFA: control group, aPD-L1 group, RFA group and RFA + aPD-L1 group. For RFA group and RFA + aPD-L1 group, radiofrequency ablation (65 °C, 20 W, 45 s) was performed on majority of the tumor tissue with sterile instruments, leaving minor residual tumor tissue alive. From Day 5, PD-L1 antibody (3.75 mg/kg) was intraperitoneally administered to mice in aPD-L1 group and RFA + aPD-L1 group every 3 days for a total of 3 times. Tumor size monitoring of mice was conducted every other day after RFA treatment. The tumor volume (mm^3) was calculated according to (length × width^2) × 0.5. Mice of all groups were euthanized on day 16, and tumor tissues were isolated for following tumor weight measurement and immunofluorescence assay.

Twelve days after tumor inoculation, the tumor-bearing mice were randomly

divided into three groups to evaluate the difference in efficacy of PD-1 antibody and PD-L1 antibody after RFA: RFA group, RFA + aPD-1 group, and RFA + aPD-L1 group. For all groups, radiofrequency ablation (65 °C, 20 W, 45 s) was performed on majority of the tumor tissue with sterile instruments, leaving minor residual tumor tissue alive. From Day 5, PD-1 antibody (3.75 mg/kg) or PD-L1 antibody (3.75 mg/kg) was intraperitoneally administered to mice in RFA + aPD-1 group and RFA+aPD-L1 group every 3 days for a total of 3 times, respectively. Tumor size monitoring of mice was conducted every other day after RFA treatment. The tumor volume (mm^3) was calculated according to (length × width^2) × 0.5. Mice of all groups were euthanized on day 15, and tumor tissues were isolated for following tumor weight measurement.

The temperature change of ablated tumors was monitored by a thermal infrared imager (Fotric, Shanghai, China).

Subpopulation analysis of immune cells

Freshly isolated tumors were first washed with cold 1× PBS. Samples were chopped, homogenized, subjected to enzymatic digestion in RPMI-1640 culture medium supplemented with 0.5 mg/mL collagenase IV (Solarbio), 0.2 mg/mL hyaluronidase (Solarbio), 0.2 mg/mL DNase (Solarbio) and incubated at 37 °C with constant shaking for 30 min. Furthermore, cells were strained using a 70-μm filter to obtain the single cell suspension. Tumor infiltrated leucocytes were isolated with mouse tumor-infiltrated leukocytes isolating kit (Solabio) and stained with diluted fluorochrome-conjugated antibodies. Freshly isolated spleens were chopped, homogenized, and incubated with 1× red blood cell lysis buffer for 10 min. After centrifugation, samples were strained using a 70-μm filter to obtain the single cell suspension and then stained with diluted fluorochrome-conjugated antibodies. The antibodies involved in the experiment include APC-Cy7 anti-CD45 (Biolegend, Catalog No.103116), FITC anti-CD3 (Biolegend, Catalog No.100204), Alexa Fluor 700 anti-CD4 (Biolegend, Catalog No.100430), PerCP-Cy5.5 anti-CD4 (Biolegend, Catalog No.100433), APC anti-CD8α (Biolegend, Catalog No. 100711), PE anti-FoxP3 (Biolegend, Catalog No.126403), PE-Cy5 anti-CD44 (Biolegend, Catalog No.), PE anti-CD62L (eBioscience, Catalog No.12-0621-81), FITC anti-CD11b (Biolegend, Catalog No.101206), Alexa Fluor 700 anti-F4/80 (Biolegend, Catalog No.123130), PE anti-Ly6G (Biolegend, Catalog No.127607), BV421 anti-CD80 (Biolegend, Catalog No.104725), PE-Cy7 anti-CD80 (Biolegend, Catalog No.104733), FITC anti-CD86 (Biolegend, Catalog No. 105109), APC anti-CD206 (Biolegend, Catalog No.141707), PE anti-CD163 (Biolegend, Catalog No. 155307), PE-Cy7 anti-CD11c (Biolegend, Catalog No. 117317), BUV661 anti CD11b (Invitrogen, Catalog No. 376-0112-80), BV570 anti-Ly6G (Biolegend, Catalog No. 127629), Alexa Fluor 488 anti-Ly6C (Biolegend, Catalog No. 128021) Spark Blue 550 anti-CD3 (Biolegend, Catalog No. 100259), Spark NIR 685 anti-CD4 (Biolegend, Catalog No. 100475), Spark UV 387 anti-CD8 (Biolegend, Catalog No. 100797), PE/Fire 810 anti-PD-1 (Biolegend, Catalog No. 135253), BV711 anti-TIM-3 (Biolegend, Catalog No. 119727). Other reagents, including the red blood cell lysis buffer (Catalog No.00-4300-54), permeabilization buffer (Catalog No.00-8333-56) and FoxP3 transcription factor staining buffer set

(Catalog No.00-5523-00) were obtained from Thermo Fisher Scientific, Zombie Aqua (Catalog No. 423101) was purchased from Biolegend, and Brilliant Stain Buffer (Catalog No. 563794) was supplied by BD (USA). Finally, the stained cells were filtered, detected by FCM (LSRFortessa, BD) or Full spectrum FCM (Cytek, Aurora), and data was analyzed by Flowjo (TreeStar,10.10.1).

RNA sequencing

Total RNA of different treated groups was extracted from the tumor tissue using TRIzol® Reagent (Invitrogen, USA) according the manufacturer's instructions and genomic DNA was removed using DNase I. RNA degradation and contamination was monitored on 1% agarose gels. The purity and concentration of RNA were determined using NanoDrop 2000 spectrophotometer (Thermo, USA). The integrity of RNA was evaluated with Agilent Bioanalyzer 2100 system (Agilent Technologies, USA) and LabChip GX (PerkinElmer, USA). Only high-quality RNA sample (OD260/280= 1.8~2.2, OD 260/230≥ 2.0, RIN≥ 8.0, 28S:18S≥ 1.0) was used to construct sequencing library. Then the sequencing library was prepared following TruSeqTM RNA sample preparation Kit from Illumina (California, USA) using 1μg of total RNA and index codes were added to attribute sequences to each sample for further clustering and analysis. After cluster generation, the library preparations were sequenced on Illumina Hiseq X and NovaSeq platform (California, USA) and paired-end reads were generated for subsequent bio-information analysis. Data analysis and processing were performed using BMKCloud (https://www.biocloud.net) and Majorbio (https://cloud.majorbio.com).

qRT-PCR assay

Isolated tumor tissues were washed and weighted to approximately 100 mg per sample; BMDMs were collected, washed, and centrifuged to pallets; TAMs were isolated from tumor tissue using FACS. The live/dead dye as well as antibodies involved in the FACS experiment include Zombie Aqua (Biolegend, Catalog No.423101), APC-Cy7 anti-CD45 (Biolegend, Catalog No. 103116), FITC anti-CD11b (Biolegend, Catalog No.101206) and PE anti-F4/80 (Biolegend, Catalog No. 123110). The total RNA was extracted using TRIzol® Reagent (Invitrogen, USA) and cDNA was synthesized by a PrimeScript RT Reagent Kit (Promega, USA). Subsequently, M1-related genes (*Tnfα*, *Nos2*, *Nfκb(p65)*, *Stat1*) and M2-related genes (*Il10*, *Arg1*, *Stat6*, *Irf4*) were analyzed by qPCR. GAPDH and PCNA were used as reference genes, and the fold change of gene expression was calculated using the $2^{-\Delta\Delta Ct}$ method. Primer sequences of target genes and reference genes are provided in Table S3.

Immunofluorescence assay

Freshly isolated tumor tissues were quickly frozen in liquid nitrogen, embedded in OCT, and then sliced to sections using freezing microtome (Leica, Germany) before been mounted onto glass slides. After been fixed with 4% paraformaldehyde and blocked with 5% BSA, the frozen sections were further incubated with primary antibodies and corresponding secondary antibodies according to the instructions of the

antibodies. The primary antibodies involved in the experiment include anti-PD-1 antibody (CST, Catalog No. 84651T, diluted at 1:400), anti-PD-L1 antibody (abcam, ab213480, diluted at 1:1000), anti-F4/80 antibody (abcam, Catalog No. ab90247, diluted at 1:1000), anti-Ly6G antibody (Servicebio, Catalog No. GB11229, diluted at 1:500), anti-CD11b antibody (abcam, Catalog No. ab8878, diluted at 1:100), anti-CD8 antibody (abcam, Catalog No. ab217344, diluted at 1:1000), anti-FoxP3 antibody (Servicebio, Catalog No. GB112325, diluted at 1:1000), anti-CD206 antibody (abcam, Catalog No. ab64693, diluted at 1:1000). Finally, the nuclei were stained with DAPI before section sealing, and all sections were scanned by Pannoramic Scan II (3D Histech, Hungary) under fluorescence channels.

Preparation and characterization of Nano-BPs and Nano-IFNγ/Zole in gel

The Nano-BPs (Nano-Zole, Nano-Alen, and Nano-Pami) were fabricated by nanoprecipitation method as previously reported with minor modifications [1]. Briefly, bisphosphonate solution (50 mM) and $CaCl_2$ solution (250 mM) were separately dispersed in mixed solvent (IGEPAL®CA-630: cyclohexane, 6.5: 3.5, v/v) under vigorous stirring. Then, the $CaCl_2$-mixed solvent system was added dropwise into bisphosphonate-mixed solvent system under gentle stirring at room temperature. Next, equivalent volume of ethanol was quickly added to the mixture under gentle stirring to form stable precipitation. The precipitation was centrifuged, washed, and resuspended with deionized water and probe ultrasound was performed to obtain the Nano-BPs. The particle size and zeta potential of Nano-BPs were characterized using Malvern Zetasizer (Nano ZS, Malvern, UK). The morphologies of the Nano-BPs were observed using TEM (JEM-1400, JEOL, Japan) and SEM (JSM-7900F, JEOL, Japan).

The fabrication of Nano-IFNγ/Zole in gel is described below. First, sodium alginate was dissolved in ultrapure water to form 4% alginate gel as concentrated gel. Then, 4% alginate gel was slowly added to Nano-IFNγ/Zole solution under gentle stirring and the Nano-IFNγ/Zole in gel was obtained when the concentration of alginate reached 2.5%. The blank alginate gel (2.5%) was prepared by adding 4% alginate gel to ultrapure water until its concentration reached 2.5%. The morphologies and element mapping of the Nano-IFNγ/Zole in gel were observed using cyro-SEM (JSM-7900F, (JEOL, Japan) equipped with Cryo Sample Preparation System (PP3010T, Quorum, Britain)). The fluorescence images of Nano-IFNγ/Zole in gel were captured by STELLARIS 8 (Leica Germany), with Nano-IFNγ/Zole labeled with 5-FAM-ZOL (BIOVINC, Catalog No. BV111001) and Cy5-IFNγ, and the 3D reconstruction of the images was performed with LAS X software (Leica, Germany). The viscosity and Young's modulus (G' and G'') was measured by rotational rheometer (MCR92, Anton Paar, Austria).

Detection of encapsulation efficiency of Nano-Zole and Nano-IFNγ/Zole

Detection of encapsulation efficiency of zoledronate was described as below. Certain amount of Nano-Zole and Nano-IFNγ/Zole powder were separately deconstructed through 0.5 M hydrochloric acid treatment overnight. Then the obtained solutions were centrifuged and the supernatants were fixed to 1.2 mL with ultra-pure

water and diluted by chromatography buffer (2.30 g ammonium dihydrogen phosphate and 5 mL tetrabutylammonium hydroxide were dissolved in ultra-pure water, fixed to 1000 mL, and adjusted to pH = 2.10 with phosphoric acid) before sample injection. Determination of encapsulation efficiency of zoledronate was performed using high performance liquid chromatography (HPLC) on LC-20AD High Performance Liquid Chromatograph (Shimadzu, Japan). The mobile phase was chromatography buffer: acetonitrile=75:25 (v/v). The chromatographic column was Ultimate Plus® C18 (150 × 4.6 mm, 5 μm) and the detected wavelength was 210 nm. The standard sample of the experiment was prepared by dissolving zoledronate to 15.0mg/ml with ultra-pure water and diluted in gradient to 30.0, 60.0, 90.0, 120.0, 150.0 μg/mL using chromatography buffer.

Detection of encapsulation efficiency of IFNγ was described as below. Certain amount of Nano-IFNγ/Zole powder with different loading amount of IFNγ (1 μg, 5 μg, 10 μg) were deconstructed through 0.5 M hydrochloric acid treatment overnight. Then the obtained solutions were centrifuged and supernatants were adjusted to pH = 7.4 before quantitative detection of IFNγ using IFNγ ELISA kit (Solarbio, Catalog No. SEKM-0031) according to the manufacturer's instructions.

Detection of encapsulation efficiency of Cy5-labeled IFNγ (Cy5-IFNγ) was described as below. Cy5-IFNγ was prepared through the coupling reaction between Cy5(1 mg/mL in DMSO) and IFNγ (2 mg/mL) for 6 h at room temperature in dark. Then the obtained mixture was transferred to a dialysis bag (COMW = 3000 Da) and subjected to dialysis in pure water for three days to remove the unreacted Cy5 before being lyophilized. The standard sample was prepared by dissolving Cy5-IFNγ to 1.5 mg/mL and diluted in gradient to 125.0, 62.5, 31.25, 15.625, 7.8125 μg/mL with ultra-pure water followed by the detection of standard curve using fluorescence spectrophotometer (Cary Eclipse, Agilent, USA). Nano-IFNγ/Zole solution with 10μg of Cy5-IFNγ loading amount was prepared and diluted to the linear range of standard curve prior to fluorescence quantitative detection.

Nanoparticle degradation behavior and in vitro drug-release profile

The degradation of BP-NPs was evaluated by centrifuging the nanoparticles to pallets and resuspending them with different aqueous medium of different pH levels (pH = 5.0 phosphate buffer, pH = 7.4 phosphate buffer, distilled water). Then the capped centrifuge tubes were places on a fixed rotator and kept rotating for 4 h at room temperature. Next, the undegraded nanoparticles were removed by centrifugation and the supernatant of each tube was collected for the measurement of Ca^{2+} concentration using ICP-OES (iCAP 7200, Thermo, USA).

The drug-release profile of Nano-IFNγ/Zole and Nano-IFNγ/Zole in gel was determined using dialysis bag method. Briefly, Nano-IFNγ/Zole or Nano-IFNγ/Zole in gel (equivalent to 100 ng IFNγ) was injected into dialysis bags (MWCO: 100 kDa) and placed into 50 mL centrifuge tubes containing release medium (pH 5.0 or pH 7.4 phosphate buffer). Then sample tubes of four groups were placed in a horizontal thermostatic rotator and kept rotating at 100 rpm, 37°C. 0.5mL of release medium was withdrawn at scheduled time points and fresh medium was replenished to each sample

tube. Finally, released IFNγ of each group was measured using IFNγ ELISA kit (Solarbio, Catalog No. SEKM-0031) according to the manufacturer's instructions.

In vivo metabolism assay of Nano-IFNγ/Zole

Determination of *in vivo* metabolism of Nano-IFNγ/Zole was evaluated using HPLC, with intravenously injected zoledronate set as positive control. For positive control panel, zoledronate (150 μL, 1 mg/mL in PBS) was intravenously injected into healthy female BALB/c mice (6–8 weeks). Blood as well as femurs and tibias of three randomly selected mice were collected at 10 min, 20 min, 40 min, 60 min post injection. Blood was mixed with anticoagulant and centrifuged to obtain plasma. 1 mL of PBS (containing protease inhibitors cocktail) were added to bone sample tube of each mouse followed by homogenization at -20 °C for 15 min. Next, sample tubes were centrifuged twice (10000 rpm, 4 °C, 10 min) and the supernatant of each sample was collected and filtrated before sample injection. Determination of zoledronate content in the plasma and bones of mice was performed using 1260 Infinity High Performance Liquid Chromatograph (Agilent, USA). Chromatography buffer was prepared by dissolving 2.30 g ammonium dihydrogen phosphate and 5mL tetrabutylammonium hydroxide in ultra-pure water, fixing to 1000 mL, and adjusting to pH = 2.10 with phosphoric acid). The mobile phase consists of chromatography buffer: acetonitrile=75:25 (v/v). The chromatographic column was SinoChrom ODS-BP (250×4.6 mm, 5 μm) and the detected wavelength was 218 nm. The standard sample of the experiment was prepared by dissolving zoledronate to 15.0 mg/mL with ultra-pure water and diluted in gradient to 0.25, 0.30, 0.35, 0.40, 0.50 0.55 μg/mL using chromatography buffer.

For Nano-IFNγ/Zole panel, a density of 5×10^5 CT26 cells were subcutaneously injected into female BALB/c mice (6–8 weeks) on the right flank. Ten days after tumor inoculation, the tumor-bearing mice were subjected to radiofrequency ablation (65 °C, 20 W, 45 s) on majority of the tumor tissue with sterile instruments, leaving minor residual tumor tissue alive. On the next day (set as day 0), Nano-IFNγ/Zole in gel was intratumorally injected into the residual tumor tissue (zoledronate was equivalent to 25 mg/kg, and IFNγ was equivalent to 10 μg/kg) of iRFA-treated mice. Blood, femurs, and tibias of three randomly selected mice were collected at 0.5 h, 2 h, 6 h, 12 h, 24 h, 48 h and 168 h post injection. Sample preparation, chromatographic conditions and zoledronate detection was similar as mentioned in the positive control panel.

Preparation and culture of BMDMs and BMDCs

BMDMs (bone marrow-derived macrophages) were acquired from femurs and tibias of C57BL/6 mice as described previously with minor modifications [2]. Briefly, the femurs and tibias were isolated from male C57BL/6 mice (6-8 weeks), sterilized in 75% ethanol, and washed with cold PBS. Both ends of the bones were removed and the bone marrow was flushed with blank culture medium using a BD syringe. After been dispersed by vigorous pipetting, the suspended cells were filtered through a 70μm cell strainer to obtain single cell suspension and washed with culture medium for two times. Then BMDMs were seeded at a density of 5×10^6/well in 6-well plate (day 0) and cultured in complete DMEM medium containing 10% fetal bovine serum (heat-

inactivated and sterile filtered), 1% penicillin, 1% streptomycin and 200 U/mL recombinant murine M-CSF at 37°C in 5% CO_2 humidified air. On day 2 and day 4, half of the culture medium of each well was collected and replenished with fresh culture medium containing 200 U/mL recombinant murine M-CSF. Cells in the collected culture medium were centrifuged, resuspended, and sent back into the original wells. The cultured BMDMs were subjected to following experiments on day 6.

BMDCs (bone marrow-derived dendritic cells) were acquired using similar protocols to that of BMDMs, except that the adopted culture medium was compete RPMI 1640 medium and that the inducible cytokine was 200 U/mL recombinant murine GM-CSF. Fresh culture medium replenishment (containing 200 U/mL GM-CSF) was also like that of BMDMs. The cultured BMDCs were subjected to following experiments on day 6.

In vitro activation of Nano-BPs on BMDCs

BMDCs were seeded in 12-well plate and treated with either Nano-Zole, Nano-Alen, and Nano-Pami at gradient concentration. Cells were incubated under optimal condition for another 24 h and then collected for FCM staining of specific surface markers. The FCM antibodies involved in the experiment include APC anti-CD11c (Biolegend, Catalog No. 117310), FITC anti-CD86 (Biolegend, Catalog No. 105109), PE-Cy7 anti-CD80 (Biolegend, Catalog No. 104733), PE anti-I-A/I-E (Biolegend, Catalog No. 107607). Finally, the stained cells were filtered, detected by FCM (FACSCalibur, BD), and analyzed by Flowjo.

In vivo anti-tumor efficacy of the Nano-Zole, Nano-Alen, and Nano-Pami after iRFA

A density of 5×10^5 CT26 cells were subcutaneously injected into female BALB/c mice (6–8 weeks) on the right flank. Twelve days after tumor inoculation, the tumor-bearing mice were randomly divided into six groups: control group, iRFA, iRFA + blank gel, RFA + Nano-Zole in gel, RFA + Nano-Alen in gel, and RFA + Nano-Pami in gel. Except for control group, mice in other groups were subjected to radiofrequency ablation (65 °C, 20 W, 45 s) on majority of the tumor tissue with sterile instruments, leaving minor residual tumor tissue alive. After one hour, the blank gel or nanoparticles-containing gel was intratumorally injected into the residual tumor tissue (BP was equivalent to 15 mg/kg) of iRFA-treated mice. Monitoring of tumor size was conducted every other day after iRFA treatment. The tumor volume (mm^3) was calculated according to (length × width^2) × 0.5. Mice of all groups were euthanized on day 20, and tumor tissues were isolated for following experiments.

Antigen-specific activation of BMDMs by Nano-IFNγ/Zole

BMDMs were seeded in 12-well plate respectively and treated with Nano-IFNγ/Zole plus B16-OVA tumor cell lysate (100 μg/mL, obtained using repeated freeze-thaw method), with several other groups set as control groups. After been incubated under optimal condition for another 24 h, cells were collected for FCM staining of specific markers of BMDMs, and cell culture supernatant was collected for

cytokines detection. The FCM antibodies involved in the experiment include FITC anti-F4/80 (Biolegend, Catalog No 123107), APC anti-CD86 (Biolegend, Catalog 105011), PE anti-SIINFEKL-H-2K^b (Biolegend, Catalog No. 141603). The stained cells were then filtered, detected by FCM (FACSCalibur, BD), and analyzed by Flowjo. The collected cell culture supernatant of BMDMs was kept for ELISA detection of TNF-α and IL-12p70 according to the manufacturer's instructions.

Repolarization of RAW264.7 cells and BMDMs by Nano-IFNγ/Zole

In the repolarization effect study of RAW264.7 cell line, cells were seeded in 12-well plate and pre-polarized to M2 phenotype (20 ng/mL IL-4 treated for 24 h). Next, cells were washed and incubated with Nano-IFNγ/Zole for another 24 h, with several other groups set as control groups. After incubation, cells were collected, washed, and stained with FCM antibodies for flow analysis.

In the repolarization effect study of BMDMs, cells were seeded in 12-well plate and pre-polarized to M2 phenotype (20 ng/ml IL-4 treated for 24 h). Next, cells were washed and treated with Nano-IFNγ/Zole for another 24h, with several other groups set as control groups. After incubation, cells were collected, washed, and stained with FCM antibodies for FCM or kept in 80 °C for further qRT-PCR experiment.

In the repolarization mechanism study of BMDMs, cells were pre-polarized to M2 phenotype and then treated with Nano-IFNγ/Zole, Nano-IFNγ/Zole + GGPP or Nano-IFNγ/Zole + squalene for 24 h. After incubation, cells were collected, washed, and stained with FCM antibodies. The antibodies involved in the experiment include Alexa Fluor 700 anti-F4/80 (Biolegend, Catalog No.123130), FITC anti-CD86 (Biolegend, Catalog No. 105109), APC anti-CD206 (Biolegend, Catalog No.141707). The stained cells were then filtered, detected by FCM (Beckman, Gallios) and analyzed by Flowjo.

Cytokine detection

Cell culture supernatant of BMDMs and BMDCs in different treated groups was collected. Then, the level of TNF-α, IL-12p70 and IL-10 in the supernatant was detected by ELISA to evaluate the activation and the reprogramming status of BMDMs.

Isolated tumor tissues were washed and weighted to approximately 100 mg per sample. Then, 3 grinding beads and 1mL of PBS (containing protease inhibitors cocktail) were added to each sample tube followed by homogenization at -20 °C for 10 min. Next, sample tubes were centrifuged twice (10000 rpm, 4 °C, 10 min) and the supernatant of each sample was collected. The level of TNF-α, IL-1β and IL-10 in the supernatant was detected by ELISA to evaluate the time-dynamics changing of TME after iRFA plus Nano-Zole or iRFA plus Nano-IFNγ/Zole treatment.

The ELISA kit involved in the experiment include TNF-α (Solarbio, Catalog No. SEKM-0034), IL-12p70 (Solarbio, Catalog No. SEKM-0013), IL-10 (Solarbio, Catalog No. SEKM-0010), IL-1β (Solarbio, Catalog No. SEKM-0002), and all procedures were performed according to the manufacturer's instructions.

Proteomics analysis using LC-MS/MS

The influence of Nano-IFNγ/Zole and GGPP on generalized protein profile of

BMDMs was evaluated by Proteomics analysis using LC-MS/MS. Total cell protein of BMDMs was obtained using RIPA Lysis Buffer supplemented with cocktail of protease inhibitors and phosphatase inhibitors. The equivalent protein was mixed with protein loading buffer and boiled at 100 °C for 10 min. After gel electrophoresis and Coomassie blue staining, protein bands were cut out and placed in centrifuge tubes followed by distained to colorlessness at 37 °C (50 mM NH_4HCO_3: acetonitrile (ACN) = 1V:1V), washed twice with ACN, and dried. Then, 5 mM dithiothreitol (DTT) was added for reduction and samples were incubated at 45 °C for 30 min, washed twice with ACN and dried. Further, samples were treated with 15 mM iodoacetamide (IAA) for 20 min, washed twice with ACN, and dried. 10 μg/mL trypsin was added to each sample and left at 4 °C for 60 min followed by 37 °C overnight. Next, 10% trifluoroacetic acid (TFA) was added to stop the zymolytic reaction, and the obtained peptide mixture was extracted twice with 50% ACN/0.1% TFA (1V:1V) and lyophilized for subsequent identification. The freeze-dried samples were redissolved in 0.1% trifluoroacetic acid solution and subjected to gradient elution for 120 min at a flow rate of 0.3 μL/min using Thermo Ultimate 3000 nano-UPLC system. The chromatographic column was Acclaim PepMap RSLC C18 (75 μm ID, 250 mm length) with ultra-pure water/formic acid as mobile phase A (99.1V:0.1V) and acetonitrile/formic acid as mobile phase B (99.1V:0.1V). The liquid chromatograph was directly connected to the Oribitrap Fusion Lumos mass spectrometer to identify the peptides. Proteome Discovery (version 2.4) software was used to search the MS/MS spectrum corresponding to each LC-MS/MS running result in the UniProt Mouse database (release 2021_03), and the proteomic identification data was output according to the search result.

Western Blotting assay

Total cell protein of BMDMs was obtained using RIPA Lysis Buffer (Beyotime, Catalog No. P0013) supplemented with cocktail of protease inhibitors and phosphatase inhibitors (Beyotime, Catalog No. P1045). Membrane protein and cytoplasmic protein of BMDMs were separated with membrane protein extraction kit (Solabio, Catalog No. EX1110). Nuclear protein and cytosol protein of BMDMs were separated with nuclear protein & cytoplasmic protein extraction kit (Beyotime, Catalog No. P0027). Furthermore, the equivalent protein (quantitative with a bicinchoninic acid protein assay kit, Beyotime, Catalog No. P0012) was mixed with protein loading buffer (Beyotime, Catalog No. P0015) and boiled at 100 °C for 5 min. After gel electrophoresis and protein transfer, the PVDF membranes were blocked and incubated with anti-Rab5 antibody (Abcam, Catalog No. ab218624, diluted at 1:1000), anti-Rab7 antibody (Abcam, Catalog No. ab218624, diluted at 1:1500), anti-STAT1 antibody (Abcam, Catalog No. ab92506, diluted at 1:1000), anti-STAT1 (phospho S727) antibody (Abcam, Catalog No. ab109461, diluted at 1:1000), anti-TFEB antibody (CST, Catalog No. 32361T, diluted at 1:1000), anti-beta tubulin antibody (Proteintech, 66240-1-IG, diluted at 1:50000) or anti-histone H3 antibody (CST, Catalog No. 4499, diluted at 1:2000), at 4 °C overnight followed by incubation with HRP-labeled goat anti-rabbit secondary antibody (Beyotime, Catalog No. A0208, diluted at 1:2000) or HRP-labeled goat anti-mouse secondary antibody (Beyotime, Catalog No. A0216, diluted at 1:2000)

for 1 h at room temperature. Electrochemiluminescence imaging was conducted with Tanon-5200 automatic chemiluminescence image analysis system (Tanon, Shanghai, China).

Lysosome acidification of BMDMs

The influence of Nano-IFNγ/Zole and GGPP on lysosome acidification of BMDMs was evaluated by CLSM. First, BMDMs were seeded in confocal dishes at a density of 6×10^5 cells per dish and cultured overnight. Then the cells were pre-polarized with IL-4 for 24 h and then incubated with Nano-IFNγ/Zole or Nano-IFNγ/Zole + GGPP for another 24 h, with untreated pre-polarized BMDMs set as control group. Next, BMDMs in the dishes were washed and incubated with 2 μM LysoSensor Green DND-189 (Yeason, Catalog No. 40767ES50) at 37 ℃ for 60 min followed by nuclei staining with Hoechst 33342 living cell stain reagent (Beyotime, Catalog No. C1027). Finally, The CLSM observation was performed on STED Confocal Microscope (Leica, Germany) and results were analyzed with LAS X software (Leica, Germany).

Detection of IFNγ release from endosomes by Nano-IFNγ/Zole in BMDMs

Cy5-labelled IFNγ (Cy5-IFNγ) was prepared as mentioned above and loaded into mineralized nanoparticles to obtain Nano-IFNγ(Cy5)/Zole. The detection of IFNγ released into the cytoplasm of BMDMs was described as below. First, BMDMs were seeded in 12-well plates at a density of 6×10^5 cells per well and cultured overnight. Then the cells were pre-polarized with IL-4 for 24 h and then incubated with free Cy5-IFNγ or Nano-IFNγ/Zole for 2 h, 4 h, 8 h and 12 h. After incubation, cells were collected and cytoplasm was isolated using nuclear protein & cytoplasmic protein extraction kit (Beyotime, Catalog No. P0027) prior to the detection on the fluorescence spectrophotometer (Cary Eclipse, Agilent, USA).

The detection of IFNγ secreted into the extracellular space of BMDMs was described as below. First, BMDMs were seeded in 12-well plates at a density of 6×10^5 cells per well and cultured overnight. Then the cells were pre-polarized with IL-4 for 24 h and then incubated with free Cy5-IFNγ or Nano-IFNγ/Zole for 8h. After incubation, nanoparticles were removed and fresh culture medium was replenished to each well, followed by 0 h, 0.5 h, 1 h, 2 h and 4 h of incubation. After incubation, cell supernatant was collected, centrifuged to removed residual cells and subjected to the detection on the fluorescence spectrophotometer (Cary Eclipse, Agilent, USA).

The CLSM observation of Nano-IFNγ(Cy5)/Zole in the cytoplasm of BMDMs was described as below. First, BMDMs were seeded in confocal dishes at a density of 6×10^5 cells per dish and cultured overnight. Then the cells were pre-polarized with IL-4 for 24 h and then incubated with Nano-IFNγ/Zole for 2 h, 4 h and 8 h. After incubation, BMDMs in the dishes were washed and incubated with 2 μM LysoSensor Green DND-189 (Yeason, Catalog No.40767ES50) at 37 ℃ for 60 min followed by nuclei staining with Hoechst 33342 living cell stain reagent (Beyotime, Catalog No. C1027). Finally, The CLSM observation was performed on AXR Confocal Microscope (Nikon, Japan) and results were analyzed with NIS-Elements Viewer AR software (Nikon, Japan).

Antigen retention and presentation capability of BMDMs

CLSM and FCM were utilized to study the influence of Nano-IFNγ/Zole and GGPP on antigen retention capability of BMDMs. First, BMDMs were seeded in confocal dishes or 12-well plates at a density of 6×10^5 cells per dish/well and cultured overnight. Then the cells were pre-polarized with IL-4 for 24 h and then incubated with Nano-IFNγ/Zole or Nano-IFNγ/Zole+GGPP for another 24 h, with untreated pre-polarized BMDMs set as control group. Next, nanoparticles were removed and fresh culture medium containing 20 μg/mL Cy5-OVA was replenished to each dish/well followed by 12 h of incubation. After incubation, culture medium in the confocal dishes or 12-well plates were replaced with fresh culture medium for further incubation. At scheduled time points, BMDMs for CLSM were washed, fixed, stained with Hoechst 33342, and reserved for detection. The CLSM observation was performed on STED Confocal Microscope (Leica, Germany) and results were analyzed with LAS X software (Leica, Germany). BMDMs for FCM were collected, washed, fixed, and reserved for detection. The FCM detection was conducted on Calibur (BD Biosciences, USA) and results were analyzed with Flowjo.

The influence of Nano-IFNγ/Zole and GGPP on antigen presentation capability of BMDMs was evaluated by FCM. Cell culture and treatment of nanoparticles were like that of antigen retention study. Next, nanoparticles were removed and fresh culture medium containing 25 μg/mL OVA was replenished to each well followed by 24 h of incubation. After incubation, cells were collected, washed, and stained with FCM antibodies. The antibodies involved in the experiment include FITC anti-CD11b (Biolegend, Catalog No.101206), Alexa Fluor 700 anti-F4/80 (Biolegend, Catalog No.123130), PE anti-SIINFEKL-H-2Kb (Biolegend, Catalog No. 141603). The stained cells were then filtered, detected by FCM (Gallios, Beckman) and analyzed by Flowjo.

Detection of IFNγ-induced PD-L1 upregulation

IFNγ-induced PD-L1 upregulation of CT26 cells was determined using FCM and CLSM. For FCM quantitative detection, CT26 cells were seeded in 12-well plates at a density of 2×10^5 cells per well and cultured overnight. Then the cells were treated with free IFNγ and Nano-IFNγ/Zole respectively (IFNγ was equivalent to 100 ng/mL) and incubated for another 24 h, with untreated cells set as control group. Finally, the cells were collected, washed, and stained with PE-Cy7 anti-PD-L1 antibody (Biolegend, Catalog No. 124313). The FCM detection was conducted on Calibur (BD Biosciences, USA) and results were analyzed with Flowjo 10.6.2.

As for CLSM qualitative detection, CT26 cells were seeded in confocal dishes (2×10^5 cells/dish) and the treatment of drugs was similar as that of FCM. After incubation, the cells were washed, fixed, blocked, and incubated with anti-PD-L1 primary antibody (abcam, ab213480, diluted at 1:1000) and corresponding secondary antibody. The CLSM observation was performed on STED Confocal Microscope (Leica, Germany) and results were analyzed with LAS X software (Leica, Germany).

In vivo anti-tumor efficacy of Nano-IFNγ/Zole after iRFA

For evaluation of the anti-tumor efficacy of Nano-IFNγ/Zole against CT26 tumor

recurrence after iRFA, tumor model was established with similar method mentioned above. A density of 5×10^5 CT26 cells were subcutaneously injected into female BALB/c mice (6–8 weeks) on the right flank. Twelve days after tumor inoculation, the tumor-bearing mice were randomly divided into five groups: RFA group, RFA + aPD-L1 group, RFA + Nano-Zole in gel group, RFA + Nano-IFNγ/Zole in gel group, and RFA + Nano-IFNγ/Zole in gel + aPD-L1 group. Mice in all groups were subjected to radiofrequency ablation (65 °C, 20 W, 45 s) on majority of the tumor tissue with sterile instruments (set as day 0), leaving minor residual tumor tissue alive. On the next day, nanoparticles-containing gel was intratumorally injected into the residual tumor tissue (zoledronate was equivalent to 22.5 mg/kg, and IFNγ was equivalent to 10 μg/kg) of iRFA-treated mice. From Day 7, PD-L1 antibody (3.75 mg/kg) was intraperitoneally administered to mice in corresponding groups every 4 days for a total of 3 times. Monitoring of tumor size and body weight was conducted every other day after iRFA treatment. The tumor volume (mm^3) was calculated according to (length × $width^2$) × 0.5. Mice of all groups were euthanized on day 21, and tumor tissues and spleens were isolated for following experiments.

For evaluation of the anti-tumor efficacy of Nano-IFNγ/Zole against CT26 distant tumor recurrence after iRFA, a density of 5×10^5 CT26 cells were subcutaneously injected into female BALB/c mice (6–8 weeks) on the right flank and a density of 5×10^5 CT26 cells were subcutaneously injected to mice on the left flank seven days later. Three days afterwards, the tumor-bearing mice were randomly divided into five groups (same as above); iRFA and drug administration were also identical to the above in situ experiment. From Day 5, PD-L1 antibody (3.75 mg/kg) was intraperitoneally administered to mice in corresponding groups every 3 days for a total of 3 times. Monitoring of tumor size and body weight was conducted every 2~3 days after iRFA treatment. The tumor volume (mm^3) was calculated according to (length × $width^2$) × 0.5. Mice of all groups were euthanized on day 16, and tumor tissues were isolated for following experiments.

For evaluation of the anti-tumor efficacy of Nano-IFNγ/Zole against *in situ* CRCLM recurrence after iRFA, tumor block transplanting method was used to establish the mouse CT26 CRCLM model. Female BALB/c mice (6–8 weeks) were inoculated with fragment (~$5mm^3$ in volume) of CT26 tumor into the right lobe of the liver. Nine days after tumor inoculation, the tumor-bearing mice were randomly divided into three groups: control group, RFA group and RFA + Nano-IFNγ/Zole in gel + aPD-L1 group, and magnetic resonance imaging (MRI) was carried out to record the tumor background before medical treatment (set as day -1). On the next day, mice in RFA group and RFA + Nano-IFNγ/Zole in gel + aPD-L1 group were subjected to radiofrequency ablation (65 °C, 20 W, 15 s) on majority of the tumor tissue with sterile instruments (set as day 0), leaving minor residual tumor tissue alive. From Day 3, PD-L1 antibody (3.75 mg/kg) was intraperitoneally administered to mice in RFA+Nano-IFNγ/Zole in gel + aPD-L1 group every 3 days for a total of 3 times. Monitoring of tumor size was conducted on day 5 and day 11 after iRFA treatment using MRI. Mice of all groups were euthanized on day 11, and tumor tissues and ascitic fluid were isolated for following experiments.

In vivo anti-tumor efficacy of non-mineralized zoledronate and IFNγ after iRFA

For evaluation of the anti-tumor efficacy of non-mineralized zoledronate and IFNγ against CT26 tumor recurrence after iRFA, tumor model was established with similar method mentioned above. A density of 5×10^5 CT26 cells were subcutaneously injected into female BALB/c mice (6–8 weeks) on the right flank. Twelve days after tumor inoculation, the tumor-bearing mice were randomly divided into five groups: RFA group, RFA + free IFNγ in gel group, RFA + free Zole in gel group, RFA + free IFNγ/free Zole in gel group, RFA + Nano-IFNγ/Zole in gel group. Mice in all groups were subjected to radiofrequency ablation (65 °C, 20 W, 45 s) on majority of the tumor tissue with sterile instruments (set as day 0), leaving minor residual tumor tissue alive. On the next day, nanoparticles-containing gel was intratumorally injected into the residual tumor tissue (zoledronate was equivalent to 22.5 mg/kg, and IFNγ was equivalent to 10μg/kg) of iRFA-treated mice. Monitoring of tumor size was conducted every other day after iRFA treatment. The tumor volume (mm^3) was calculated according to (length × $width^2$) × 0.5. Mice of all groups were euthanized on day 9, and tumor tissues were isolated for following experiments.

Immunohistochemical assay

Isolated tumor tissues were fixed with 4% paraformaldehyde for more than 24 h, embedded in paraffin blocks by dehydration, sliced to sections using rotary microtome (Leica, Germany) before been mounted onto glass slides. Then the paraffin sections were dewaxed and rehydrated prior to antigen retrieval. After the blockade of endogenous peroxidase (in 3% hydrogen peroxide) and nonspecific binding (in 5% BSA), the paraffin sections were further incubated with anti-Ki67 primary antibody (Abcam. Catalog No. ab16667, diluted at 1:200) and corresponding secondary antibodies according to the instructions of the antibodies. DAB (Sigma, Catalog No. D8001) was used as the chromogen and DAPI was used for nuclei staining. All sections were subsequently dehydrated, sealed and scanned by Pannoramic Scan II (3D Histech, Hungary) under bright field.

H&E staining

Paraffin sections of tumors and major organs were prepared using the similar method as the immunohistochemical assay. The prepared paraffin sections were dewaxed and rehydrated prior to staining. Sections were first stained with hematoxylin for 3 min and 1% hydrochloride acid ethanol for another 5 s and rinsed with 0.6% ammonia until sections turn blue. Eosin staining was performed for 30 s followed by dehydration. Finally, sections were subsequently dehydrated, sealed and scanned by Pannoramic Scan II (3D Histech, Hungary) under bright field.

TUNEL assay

Terminal deoxynucleotidyl transferase (TdT) dUTP Nick-End Labeling (TUNEL) assay was performed to determine the apoptosis level of tumor tissues. Frozen sections of freshly isolated tumor tissues were prepared using the similar method as the

immunofluorescence assay**.** The prepared frozen sections were dehydrated and fixed with 4 % paraformaldehyde followed by 0.1% Triton X-100 permeabilization. Sections were then incubated in 1×PBS and dried using tissue paper. Furthermore, the labeling reaction was set up by adding 50 μL of TUNEL reaction mixture on the frozen sections and sections were incubated at 37 °C for 1 h in a humidified chamber away from light. Finally, the nuclei were stained with DAPI before section sealing, and all sections were scanned by Pannoramic Scan II (3D Histech, Hungary) under fluorescence channels.

Biosafety analysis

Biosafety of Nano-IFNγ/Zole was evaluated by mice body weight monitoring, blood analysis and H&E staining of major organs. During the observation of *in vivo* anti-tumor immunotherapy post iRFA, the mice were weighed every other day. On the end day of experiment, mice serum and whole blood were extracted for blood biochemical and blood routine analysis, while the major organs (heart, liver, spleen, lungs, and kidneys) were isolated for H&E staining.

Furthermore, the difference in the biosafety of administration mode of IFNγ was also evaluated, and tumor model was established with similar method mentioned above. A density of 5×10^5 CT26 cells were subcutaneously injected into female BALB/c mice (6–8 weeks) on the right flank. Twelve days after tumor inoculation, the tumor-bearing mice were randomly divided into four groups: RFA group, RFA + free IFNγ (i.v.) group, RFA + free IFNγ in gel (i.t.) group, RFA + Nano-IFNγ/Zole in gel group. Mice in all groups were subjected to radiofrequency ablation (65 °C, 20 W, 45 s) on majority of the tumor tissue with sterile instruments, leaving minor residual tumor tissue alive. On the next day (set as day 0), free IFNγ or Nano-IFNγ/Zole in gel was administered through indicated routes (zoledronate was equivalent to 25mg/kg, and IFNγ was equivalent to 10μg/kg). Monitoring of body weight was conducted every other day after iRFA treatment. 1 day, 2 days and 7 days after IFNγ administration, three mice in each group were randomly selected and then euthanized. Serum and tumor tissues were collected for cytokine detection. ELISA kits involved in the experiment include IFNγ (Solarbio, Catalog No. SEKM-0031), TNF-α (Solarbio, Catalog No. SEKM-0034), IL-12p70 (Solarbio, Catalog No. SEKM-0013), IL-6 (Solarbio, Catalog No. SEKM-0007), CXCL10 (Solarbio, Catalog No. SEKM-0049), CCL2 (Solarbio, Catalog No. SEKM-0108) and all procedures were performed according to the manufacturer's instructions. Spleens and livers were isolated for H&E staining to evaluated the pathological injury of organs.

Statistical Analysis

Data were analyzed by two-tailed unpaired t tests or one-way ANOVA with Tukey's multiple comparisons within GraphPad Prism 8 software, and quantitative statistics were represented as mean ± standard deviation (SD). Statistical significance was set as *P <0.05, **P < 0.01, ***P < 0.001, ns, no significance.

Supplementary Figures and Tables

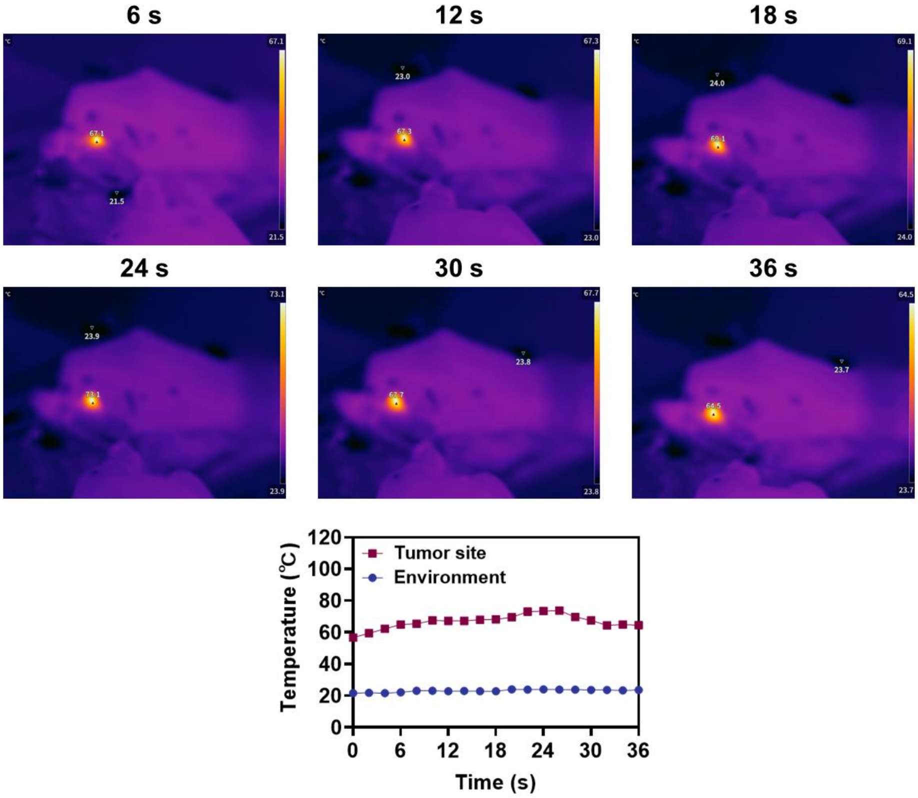

Figure S1 Representative images and the variation of local temperature of tumor tissue during iRFA (65 °C, 20 W).

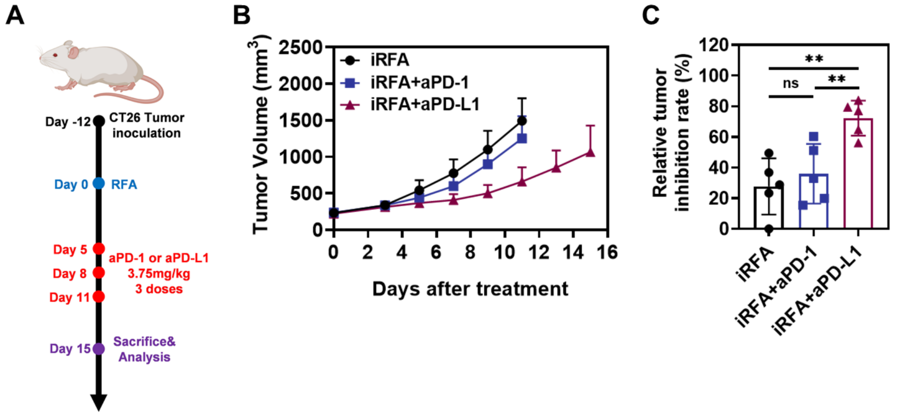

Figure S2 (A) Schematic illustration of the assessment of the different *in vivo* anti-tumor efficacy of PD-1 and PD-L1 antibody after iRFA. (B) Growth curves of tumor volume after different treatment (n = 5). (C) Relative tumor inhibition rate based on tumor weight (n = 5). All statistical data are presented as mean ± SD; data were analyzed with two-tailed unpaired t tests; ns, no significance; **, $p < 0.01$.

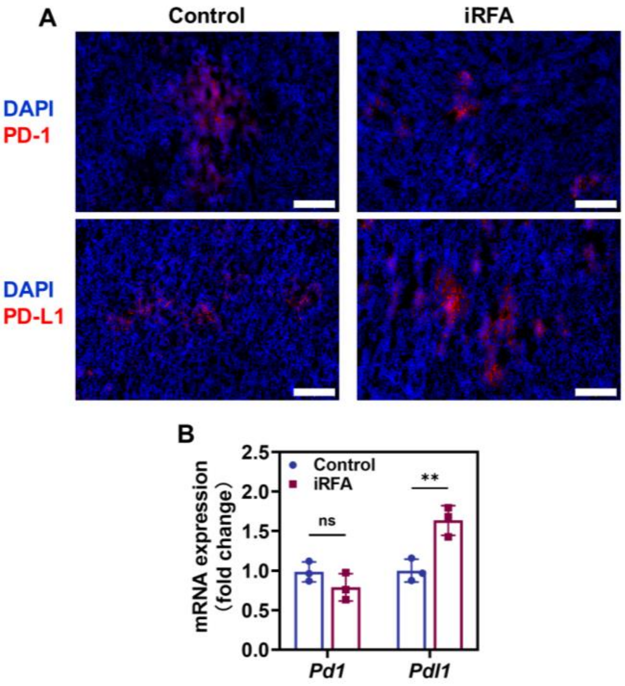

Figure S3 (A) Representative immunofluorescence images of CT26 tumor tissue (staining of PD-1 or PD-L1) with or without iRFA treatment, scale bar: 200 μm. (B) qRT-PCR analysis of *Pd1* gene and *Pdl1* gene expressed in CT26 tumor tissue with or without iRFA treatment (n = 3). All statistical data are presented as mean ± SD; data were analyzed with two-tailed unpaired t tests; ns, no significance; **, p < 0.01.

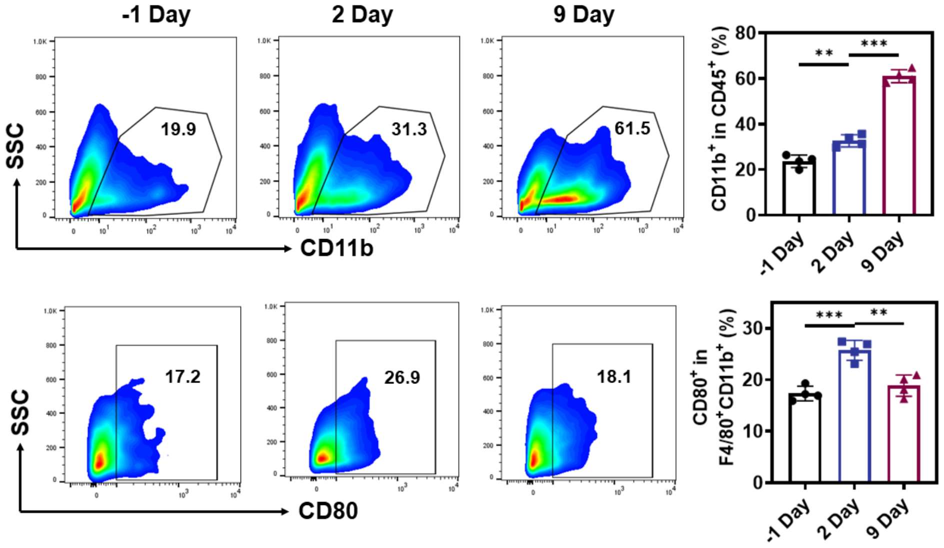

Figure S4 Representative flow cytometry plots and corresponding quantification of tumor-infiltrating monocytes and M1-type macrophages on different days before or after iRFA treatment (n = 4). All statistical data are presented as mean ± SD; Data were analyzed with two-tailed unpaired t tests; **, p < 0.01; ***, p < 0.001.

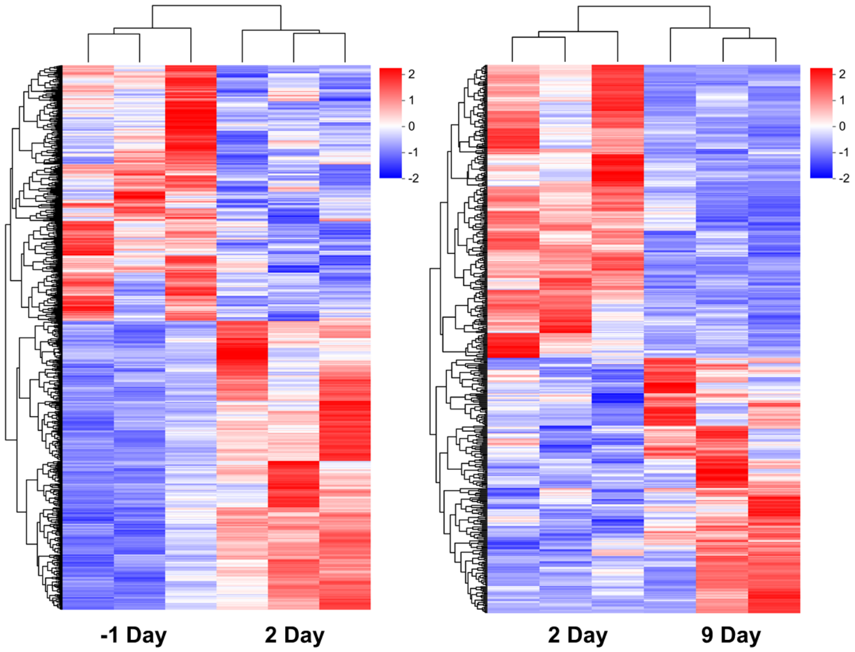

Figure S5 Heat plots of differentially expressed genes (DEGs) in tumor tissue on different days before or after incomplete RFA treatment (n = 3).

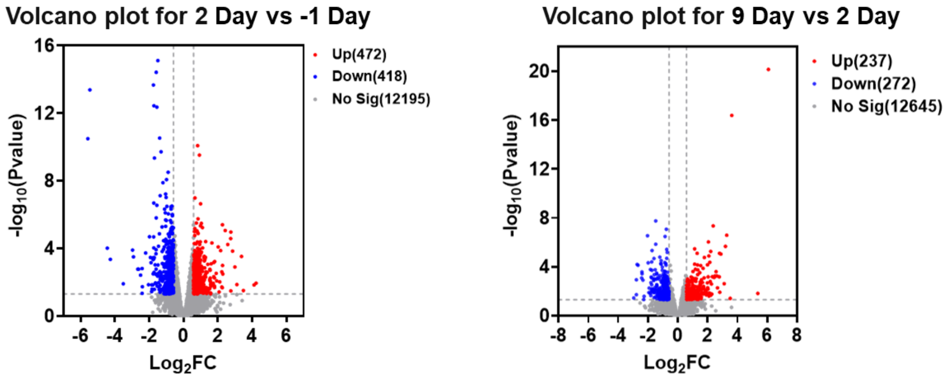

Figure S6 Volcano plot of DEGs in tumor tissue between different groups before or after iRFA treatment (n = 3).

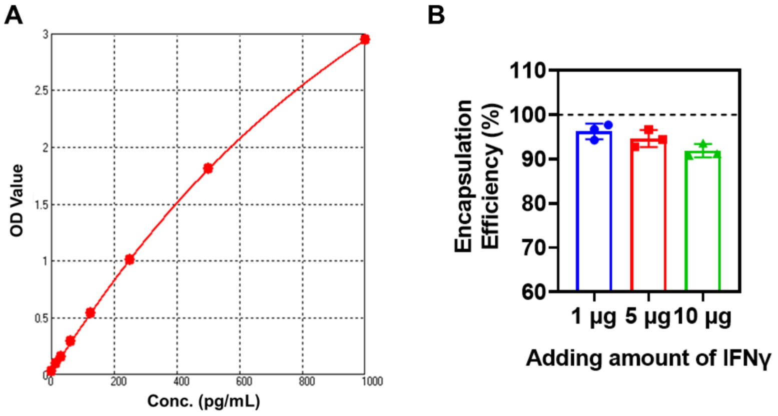

Figure S7 (A) ELISA standard curve of IFNγ. (B) Quantitative analysis of the encapsulation efficiency of IFNγ in Nano-IFNγ/Zole at different adding amount of IFNγ (n = 3).

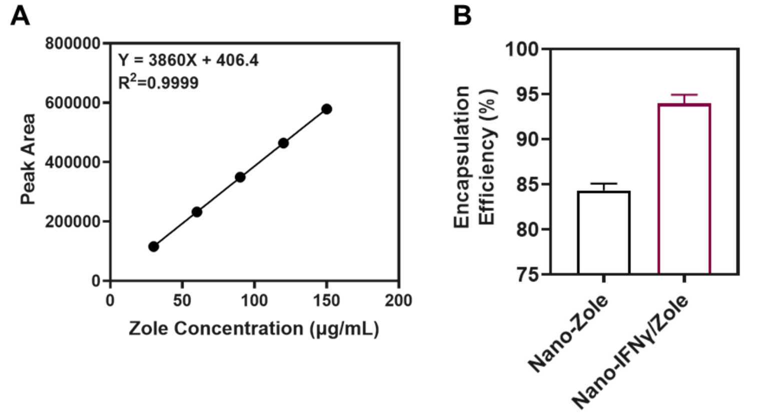

Figure S8 (A) HPLC standard curve of zoledronate in mobile phase. (B) Quantitative analysis of the encapsulation efficiency of zoledronate in Nano-Zole and Nano-IFNγ/Zole (n = 3).

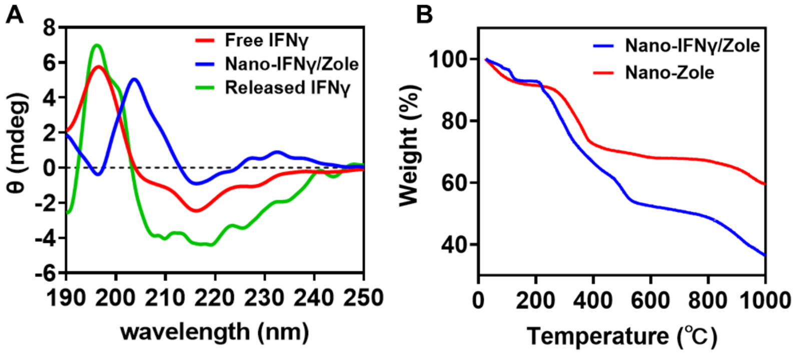

Figure S9 (A) Circular dichroism spectrum analysis of free IFNγ, Nano-IFNγ/Zole and IFNγ released from Nano-IFNγ/Zole. (B) Thermogravimetric analysis of Nano-Zole and Nano-IFNγ/Zole.

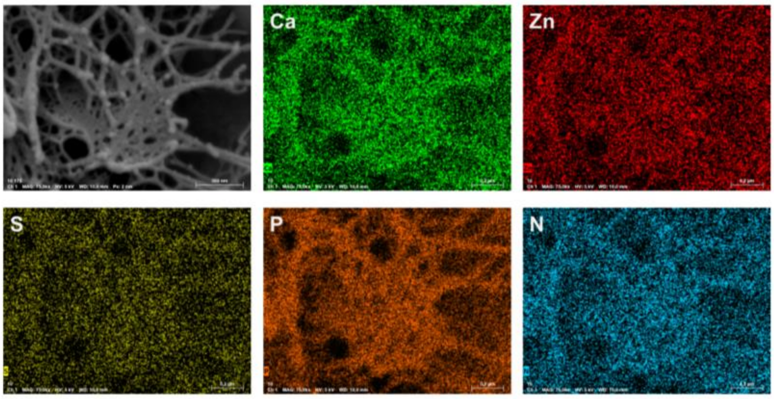

Figure S10 Element mapping of Nano-IFNγ/Zole in gel using cyro-SEM.

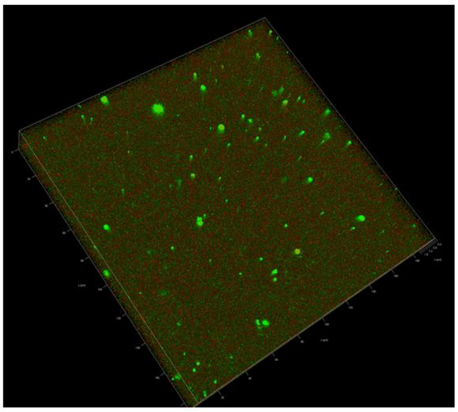

Figure S11 3D reconstruction images of Nano-IFNγ/Zole in gel (zoledronate labelled with 5-FAM, green; IFNγ labeled with Cy5, red).

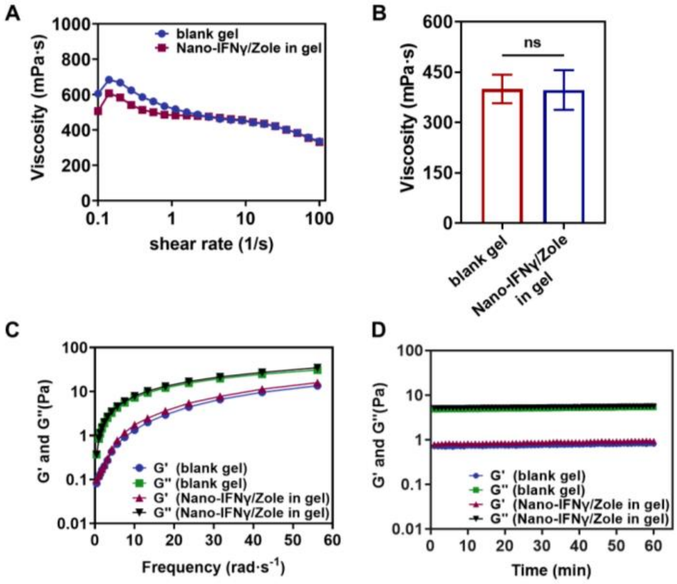

Figure S12 (A-B) Variation of viscosity of Nano-IFNγ/Zole in gel and blank gel with sheer rate (A) and corresponding quantification (B) (n=3). (C) Variation of Young's

modulus of Nano-IFNγ/Zole in gel and blank gel with angular frequency. (D) Variation of Young's modulus of Nano-IFNγ/Zole in gel and blank gel with time. ns, no significance.

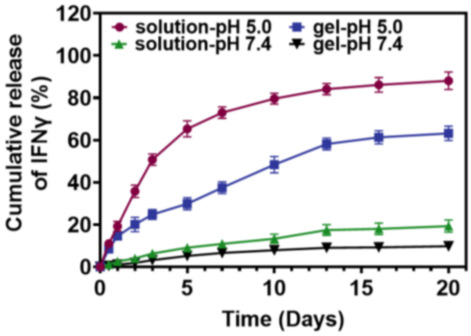

Figure S13 IFNγ released from Nano-IFNγ/Zole or Nano-IFNγ/Zole in gel in pH 5.0 or pH 7.4 phosphate buffer (n = 3).

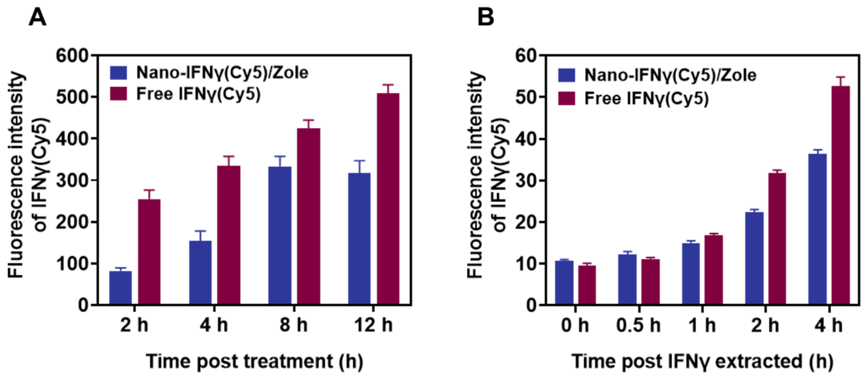

Figure S14 (A) Fluorescence quantitative analysis of IFNγ (Cy5) released into the cytoplasm at different time points after treatment (n = 3). (B) Fluorescence quantitative analysis of IFNγ(Cy5) released into the cell culture medium at different time points after IFNγ(Cy5) or Nano-IFNγ(Cy5)/Zole was extracted (n = 3).

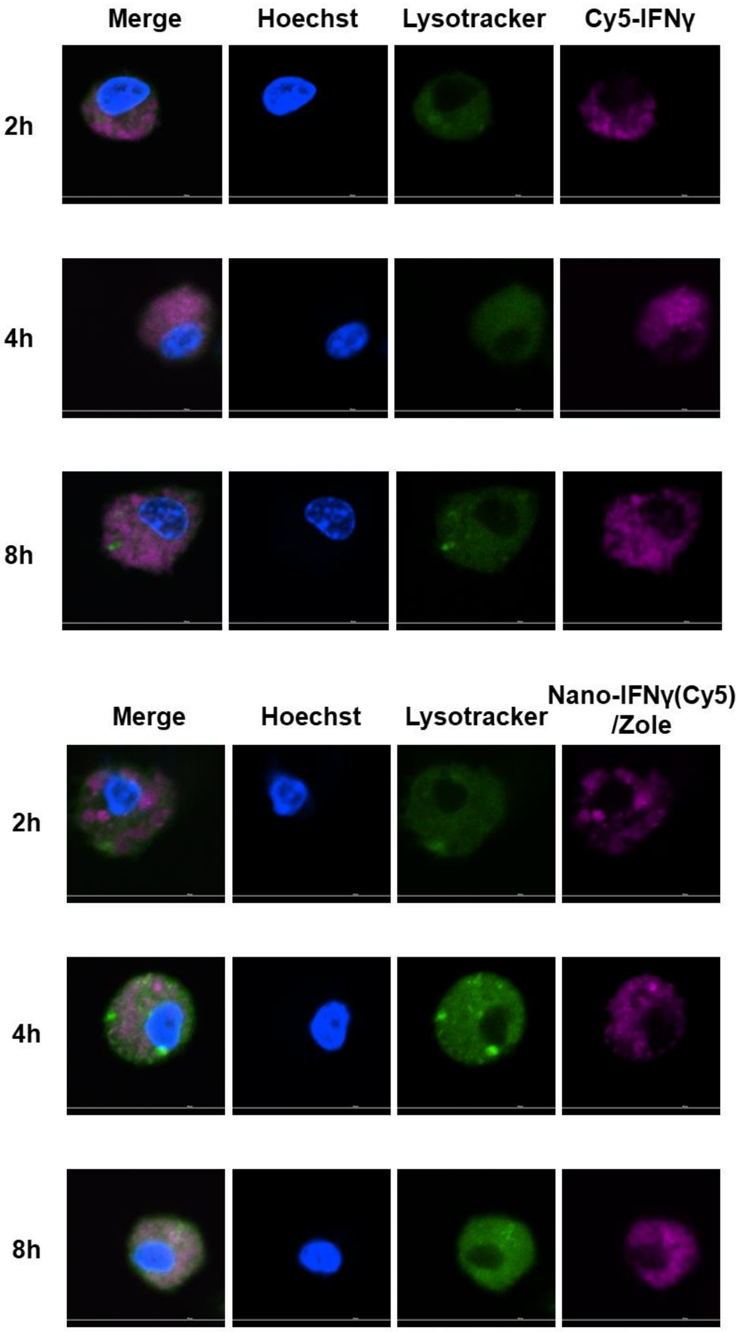

Figure S15 Variation of fluorescence signal of Cy5 in BMDMs at different time points after free Cy5-IFNγ or Nano-IFNγ(Cy5)/Zole incubation. Nano-IFNγ/Zole was labeled with Cy5-IFNγ.

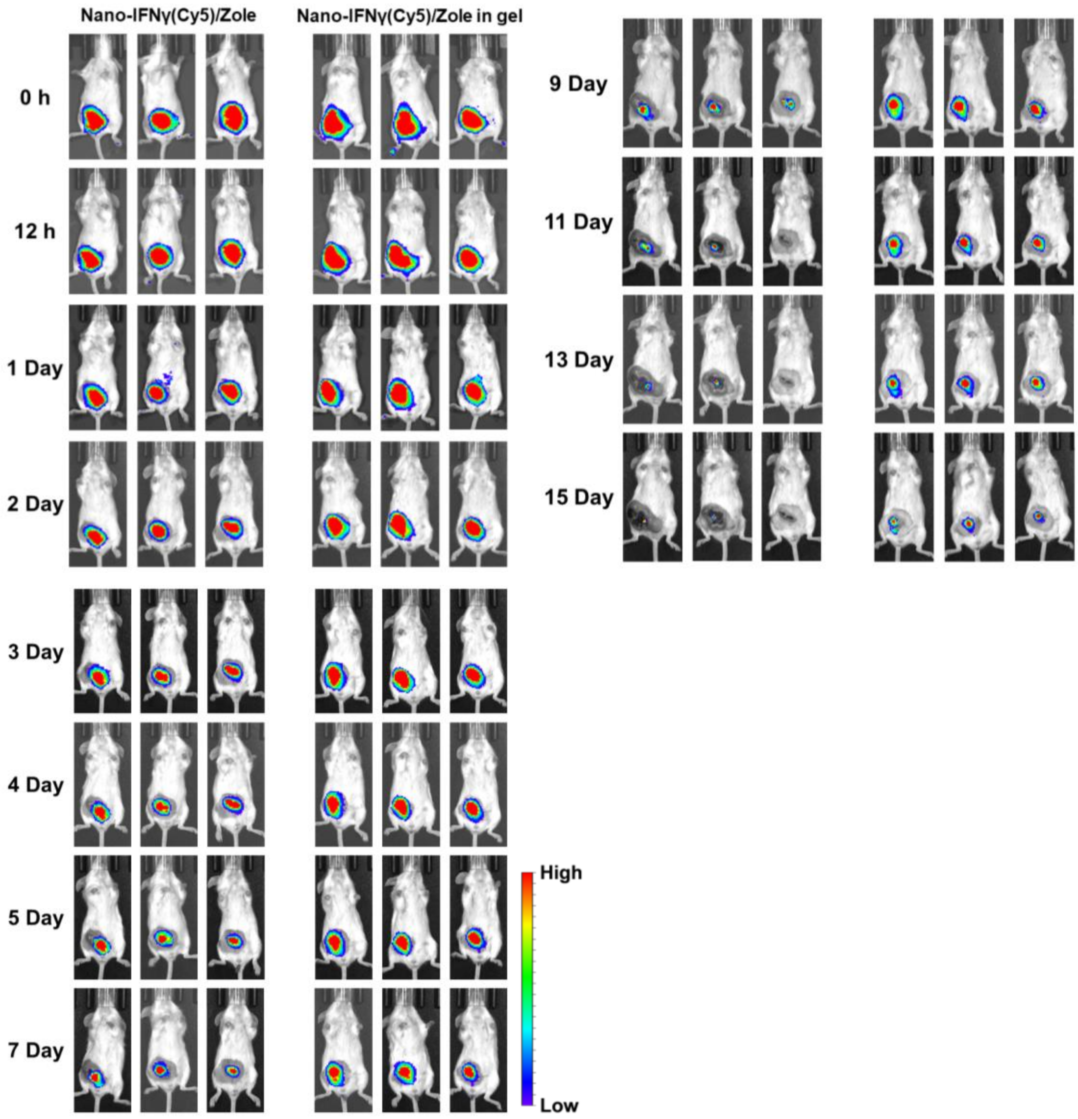

Figure S16 IVIS images of pretreated dead tumor of the tumor-bearing mice at different time points after Nano-IFNγ/Zole or Nano-IFNγ/Zole in gel administration (n = 3). Nano-IFNγ/Zole was labeled with Cy5-IFNγ.

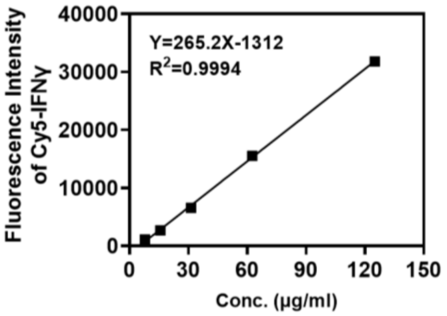

Figure S17. Fluorescence standard curve of Cy5-IFNγ. The encapsulation efficiency of Cy5-IFNγ in Nano-IFNγ(Cy5)/Zole was 92.29±2.46% according to the standard curve calculation.

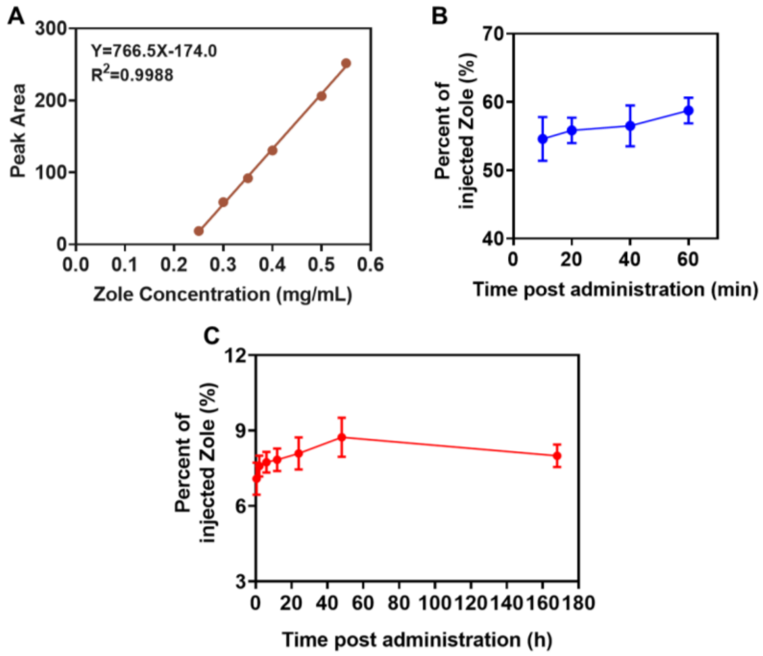

Figure S18 (A) HPLC standard curve of zoledronate in mobile phase. (B) Quantitative analysis of zoledronate in the femurs and tibias of mice at different time points after intravenous injection of free zoledronate (n = 3). (C) Quantitative analysis of zoledronate in the femurs and tibias of mice at different time points after intratumoral injection of Nano-IFNγ/Zole in gel (n = 3).

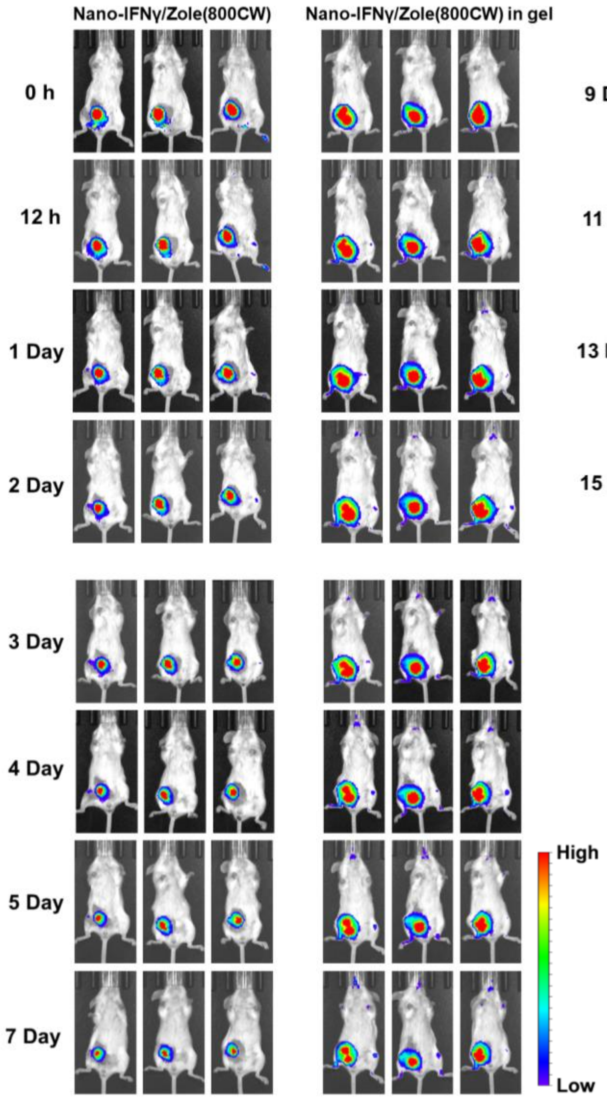

Figure S19 IVIS images of pretreated dead tumor of the tumor-bearing mice at different time points after Nano-IFNγ/Zole or Nano-IFNγ/Zole in gel administration (n = 3). Nano-IFNγ/Zole was labeled with 800CW-ZOL (BIOVINC, Catalog No. BV551001).

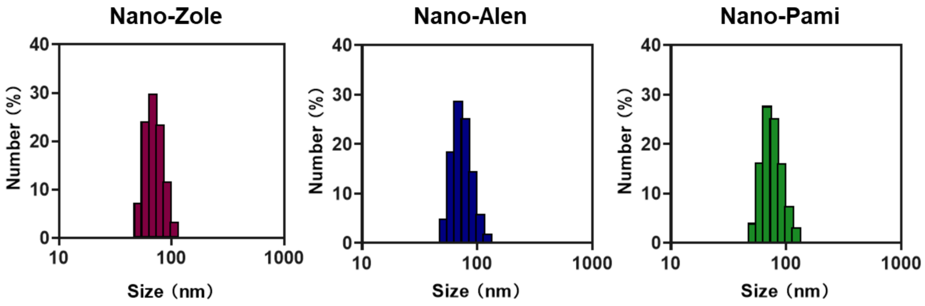

Figure S20 Particle size distributions of IFNγ-unloaded Nano-Zole, Nano-Alen and Nano-Pami, measured by DLS.

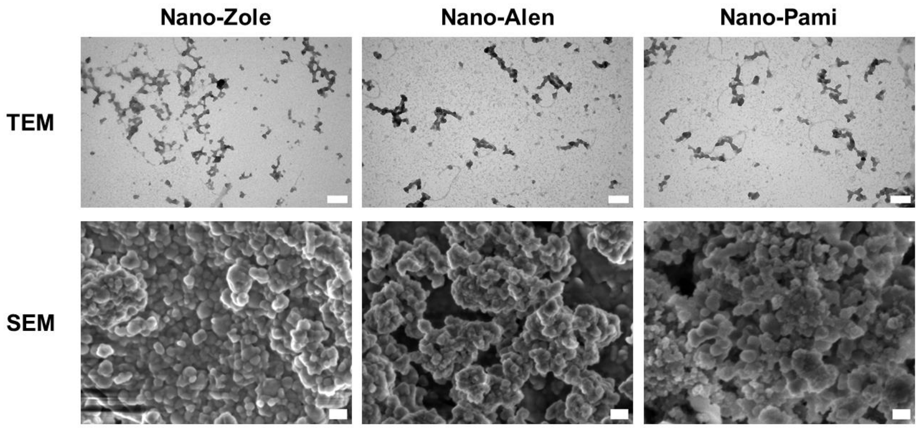

Figure S21 Representative TEM images (B, scale bar: 100 nm) and SEM images (C, scale bar: 200 nm) of Nano-Zole, Nano-Alen and Nano-Pami.

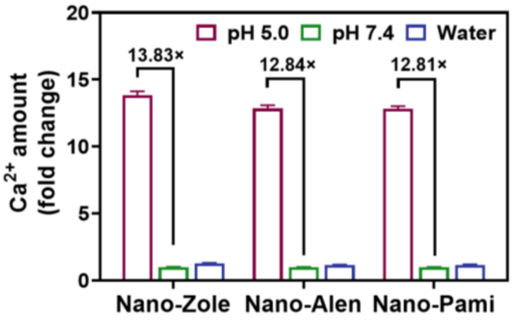

Figure S22 The amount of Ca^{2+} released from Nano-Zole, Nano-Alen and Nano-Pami, after incubated in different medium for 4 h (n = 3).

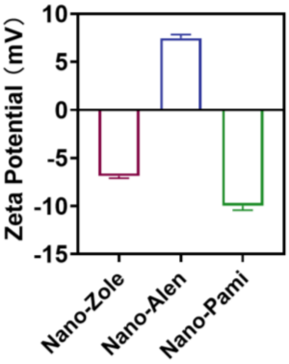

Figure S23 The zeta potentials of Nano-Zole, Nano-Alen and Nano-Pami measured by DLS (n = 3).

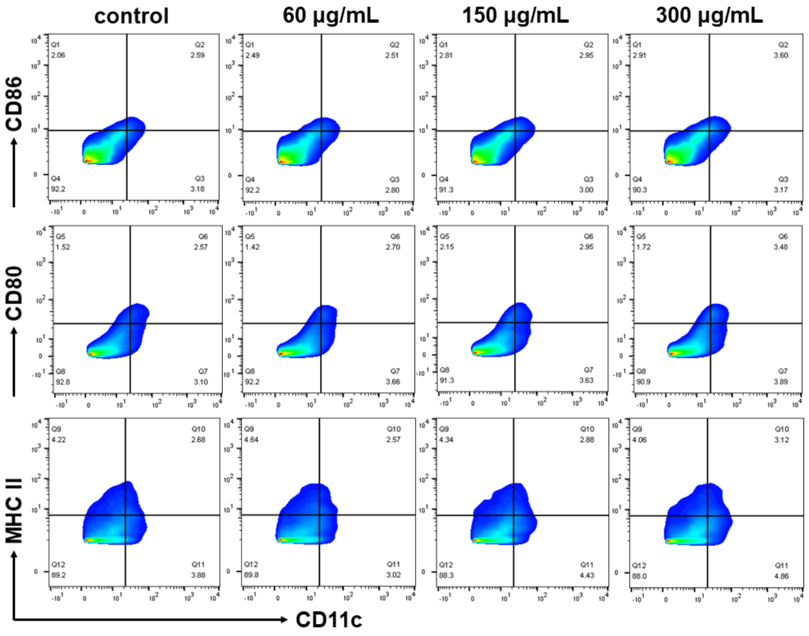

Figure S24 Representative flow cytometry plots of BMDCs after treated with different concentration of Nano-Alen.

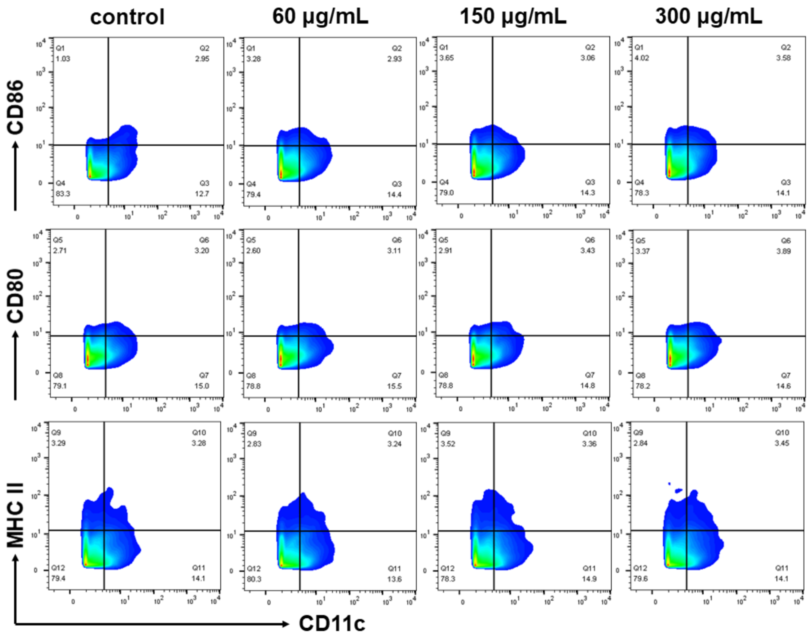

Figure S25 Representative flow cytometry plots of BMDCs after treated with different concentration of Nano-Pami.

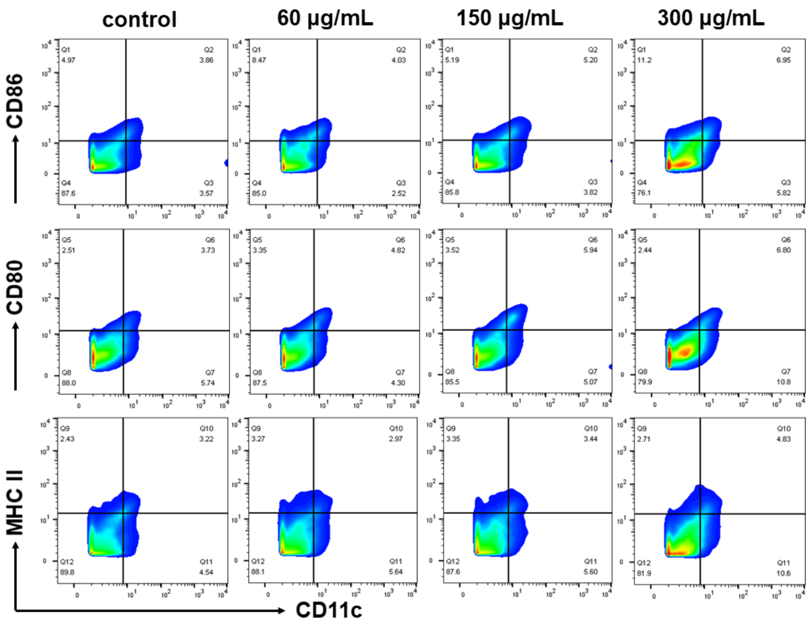

Figure S26 Representative flow cytometry plots of BMDCs after treated with different concentration of Nano-Zole.

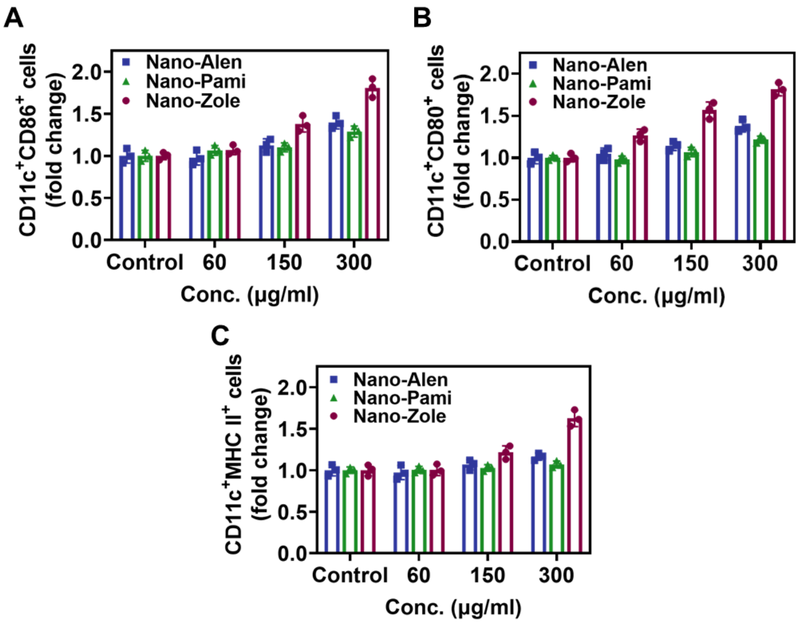

Figure S27 Quantification of flow cytometry analysis of flow cytometry detection of CD86 (A), CD80 (B), and MHC II (C) expression on CD11c$^+$ BMDCs after Nano-Zole, Nano-Alen and Nano-Pami incubation (n = 3).

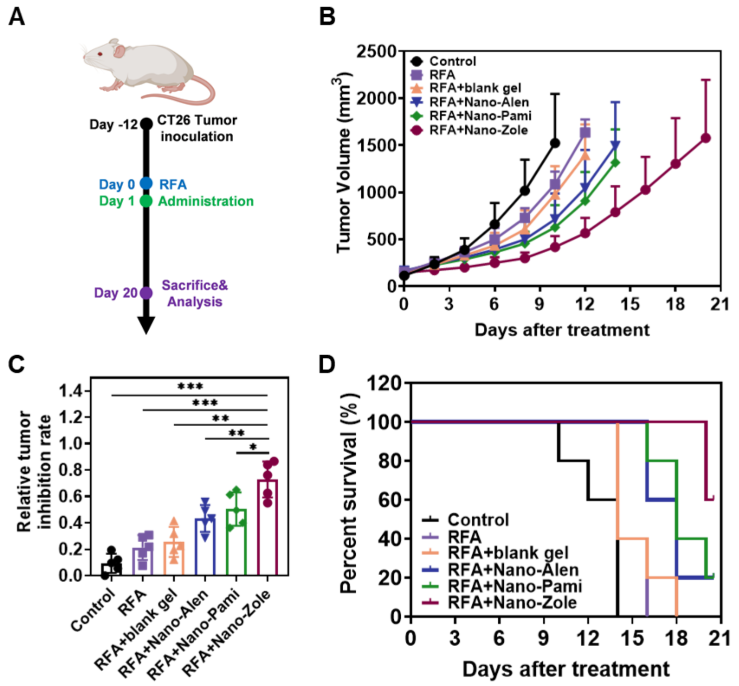

Figure S28 (A)The schematic illustration of the assessment of the *in vivo* anti-tumor effect of Nano-Zole, Nano-Alen and Nano-Pami. (B) Growth curves of the tumor volume after different treatment (n = 5). (C) Relative tumor inhibition rate of all experiment groups based on tumor weight (n = 5). (D) Survival curves of all experiment groups (n = 5). *, $p < 0.05$; **, $p < 0.01$; ***, $p < 0.001$.

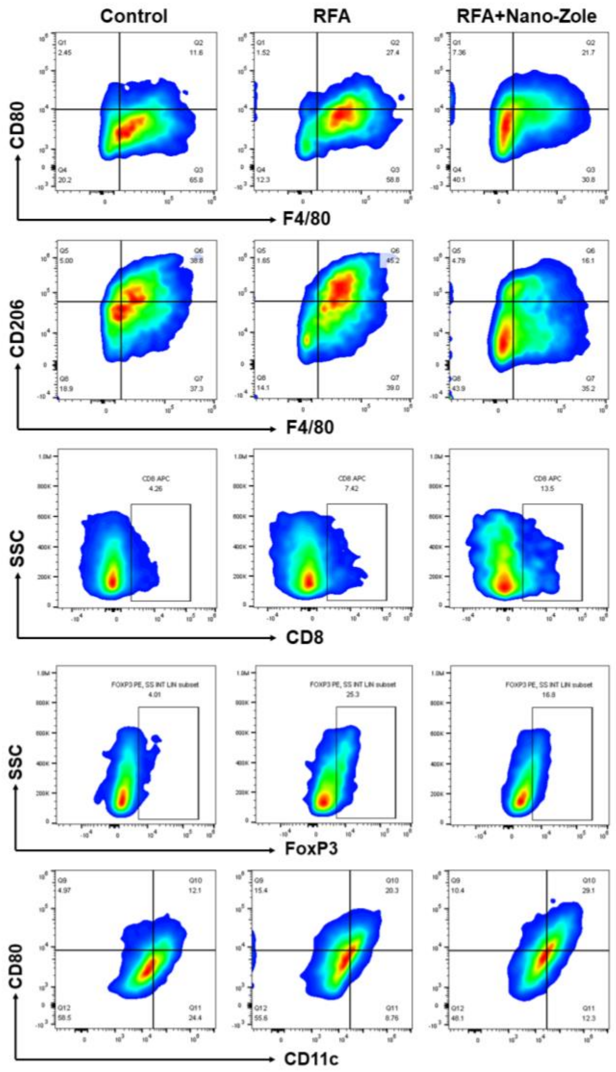

Figure S29 Representative flow cytometry plots of tumor-infiltrating M1-TAMs, M2-TAMs, CD8+ T cells, T_{reg} cells as well as matured DC cells after different treatment.

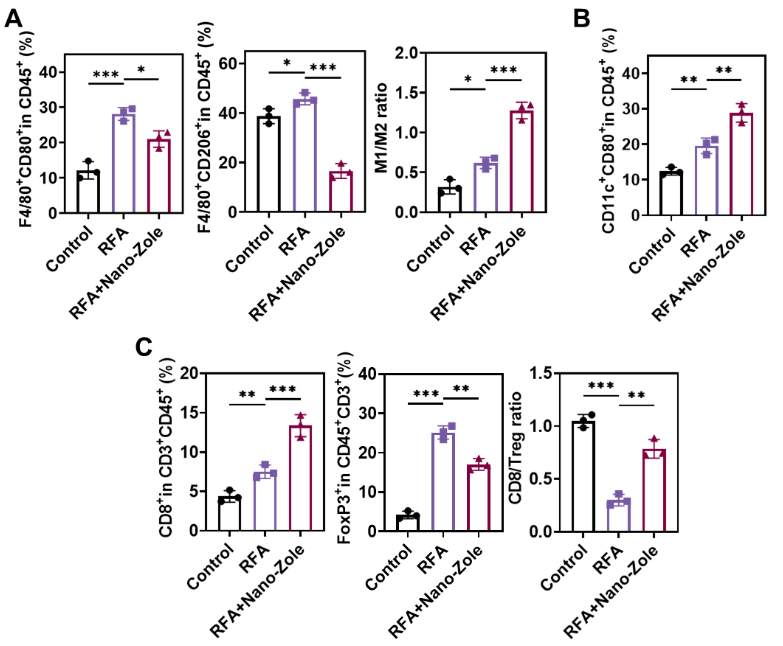

Figure S30 Quantification of flow cytometry analysis of tumor-infiltrating M1-TAMs, M2-TAMs, and their ratio (A), matured DC cells (B), as well as $CD8^+$ T cells, T_{reg} cells and their ratio (C) after iRFA and iRFA + Nano-Zole treatment (n = 3). *, $p < 0.05$; **, $p < 0.01$; ***, $p < 0.001$.

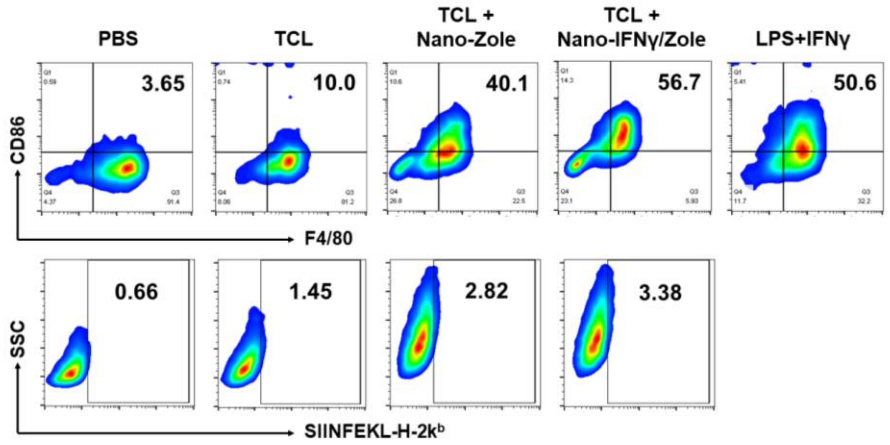

Figure S31 Representative flow cytometry plots BMDMs after different treatment. The in vitro activation status of BMDMs was measured by detecting the expression of CD86 in $F4/80^+$ BMDMs. The antigen cross-presentation ability of BMDM cells was measured by detecting the expression of SIINFEKL-bounded H2-K^b in $F4/80^+$ BMDMs.

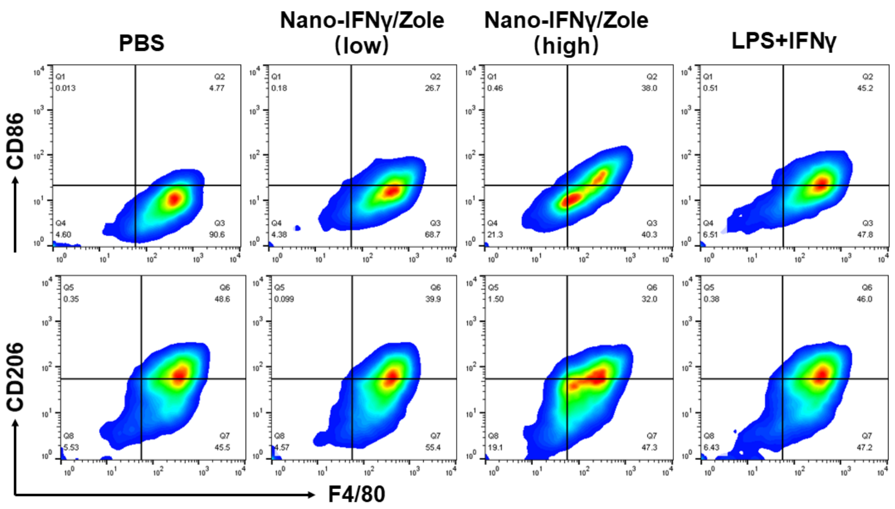

Figure S32 Representative flow cytometry plots of M1 and M2 markers on BMDMs after different treatment.

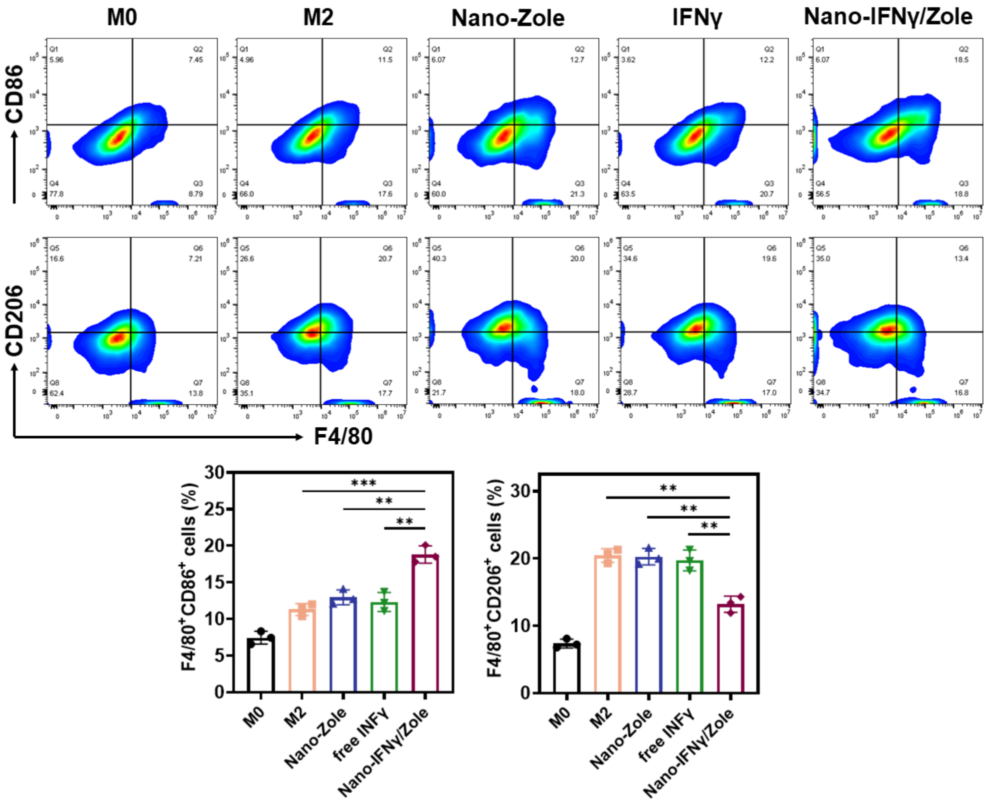

Figure S33 Representative flow cytometry plots and corresponding quantification of M1 and M2 markers on RAW 264.7 cells after different treatment (n = 3). All statistical data are presented as mean ± SD; Data were analyzed with two-tailed unpaired t tests; **, $p < 0.01$; ***, $p < 0.001$.

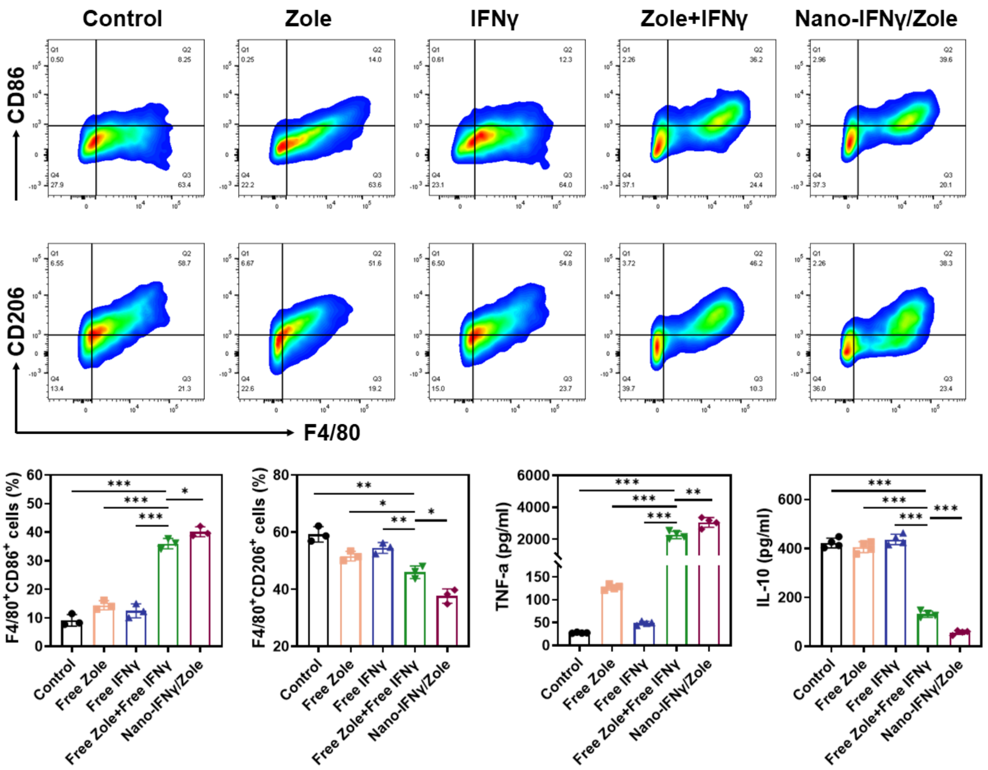

Figure S34 Representative flow cytometry plots and corresponding quantification of M1 and M2 markers on BMDMs (n = 3) as well as cytokines secreted by BMDMs (n = 4) after different treatment. All statistical data are presented as mean ± SD; Data were analyzed with two-tailed unpaired t tests; *, $p < 0.05$; **, $p < 0.01$; ***, $p < 0.001$.

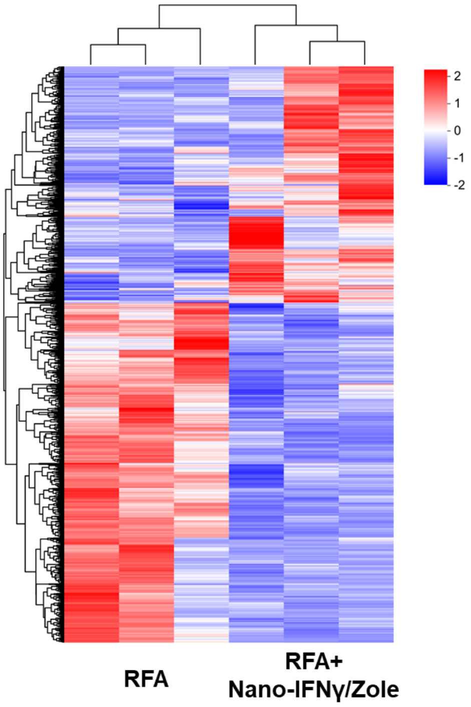

Figure S35 Clustering heat map of DEGs in CT26 tumors after iRFA or iRFA + Nano-IFNγ/Zole treatment (n = 3).

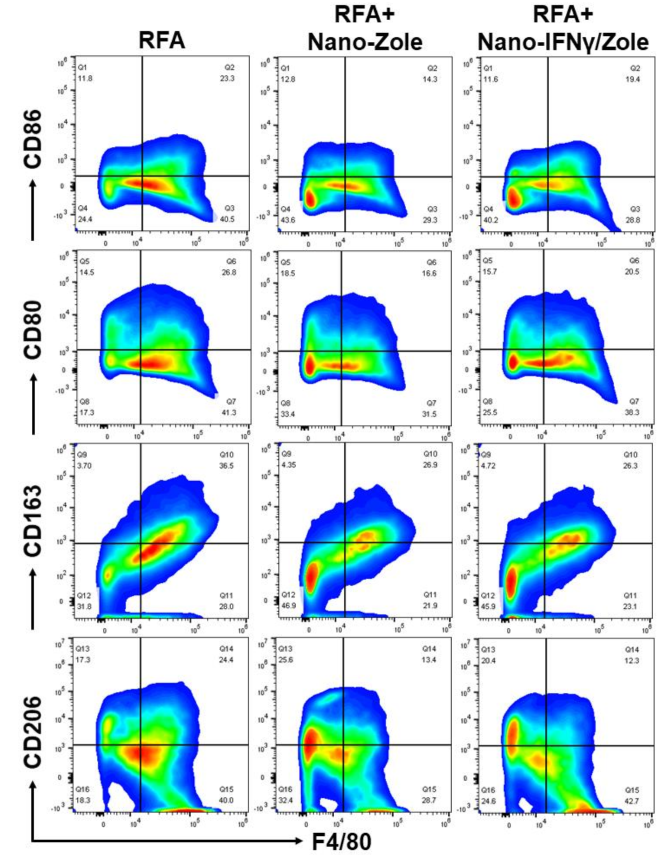

Figure S36 Representative flow cytometry plots of M1 and M2 markers on TAMs 2 days after different treatment.

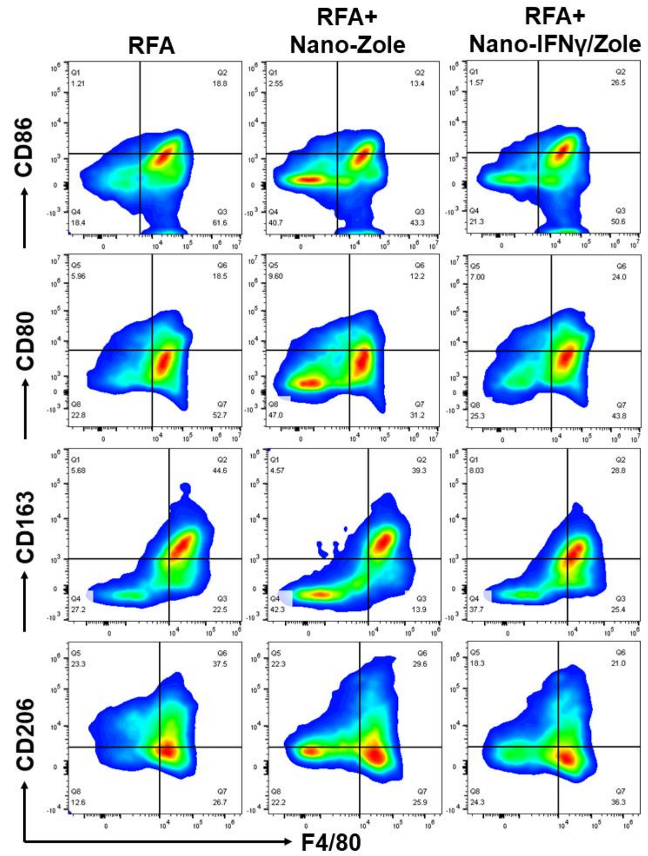

Figure S37 Representative flow cytometry plots of M1 and M2 markers on TAMs 9 days after different treatment.

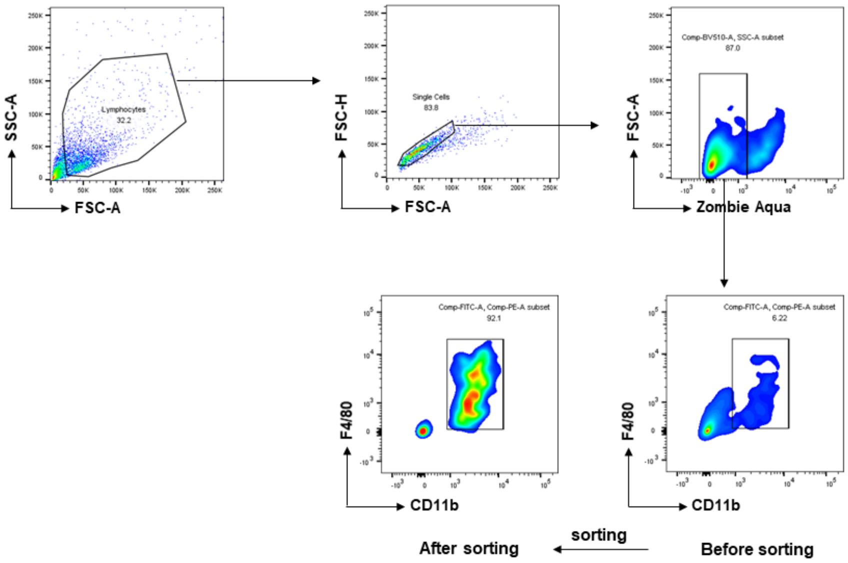

Figure S38 Gating strategy and sorting efficiency of TAMs using FACS.

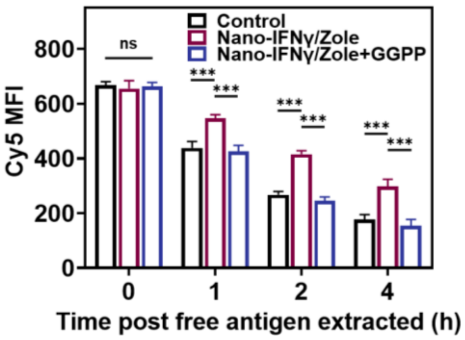

Figure S39 Flow cytometry analysis of Cy5-labeled OVA retained in M2-BMDMs at different time post Cy5-OVA extracted (n = 3). BMDMs were pre-incubated with Nano-IFNγ/Zole or Nano-IFNγ/Zole + GGPP. All statistical data are presented as mean ± SD; Data were analyzed with two-tailed unpaired t tests; ns, no significance; ***, p < 0.001.

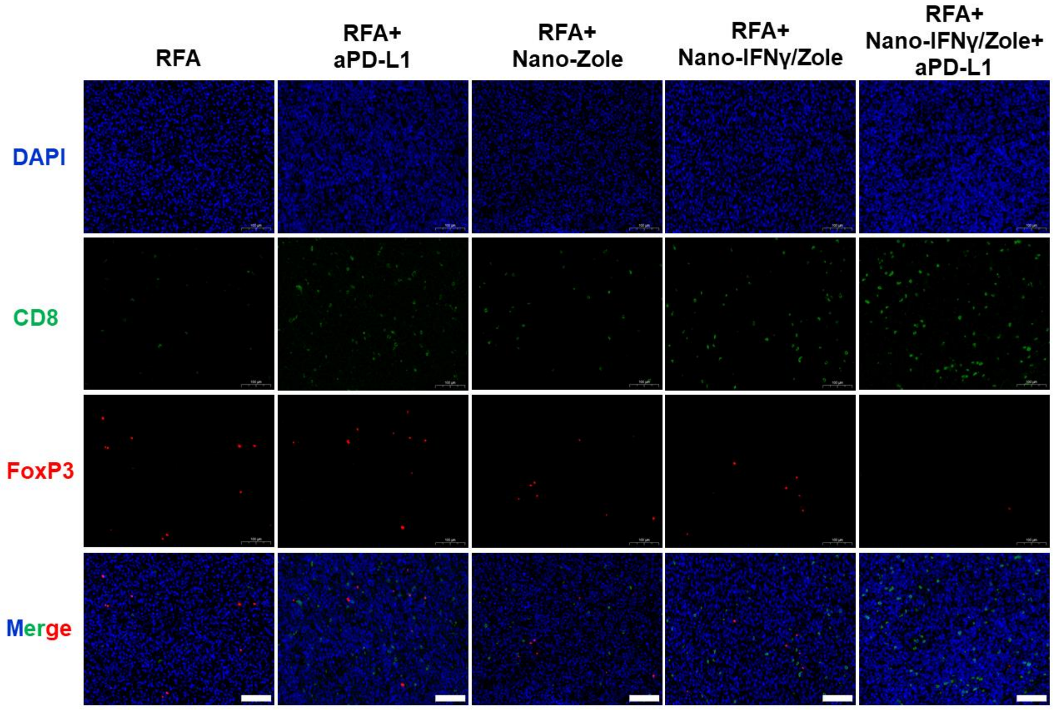

Figure S40 Representative immunofluorescence images of tumor infiltrating T cells after different treatment, scale bar: 100 μm.

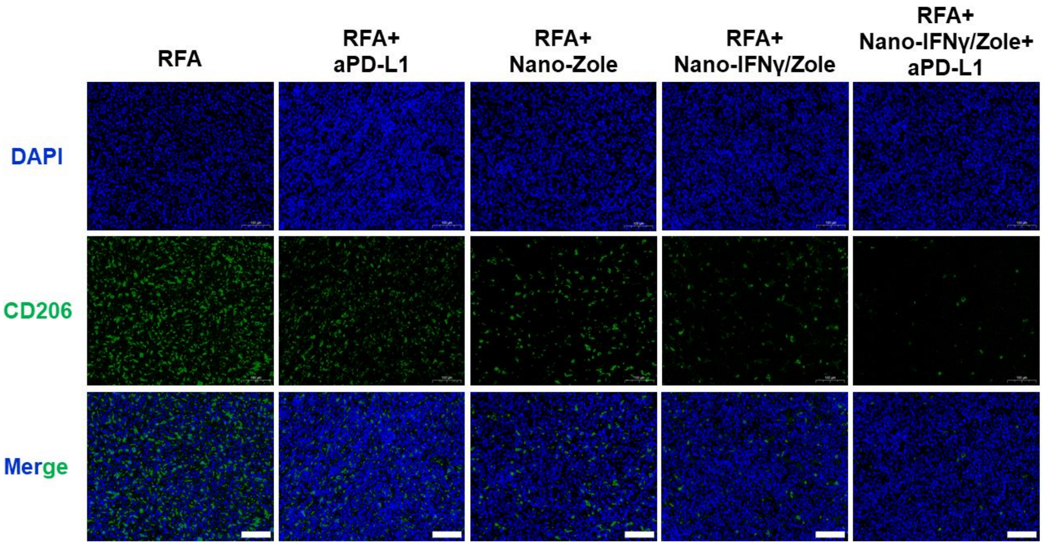

Figure S41 Representative immunofluorescence images of tumor infiltrating M2-type TAMs after different treatment, scale bar: 100 μm.

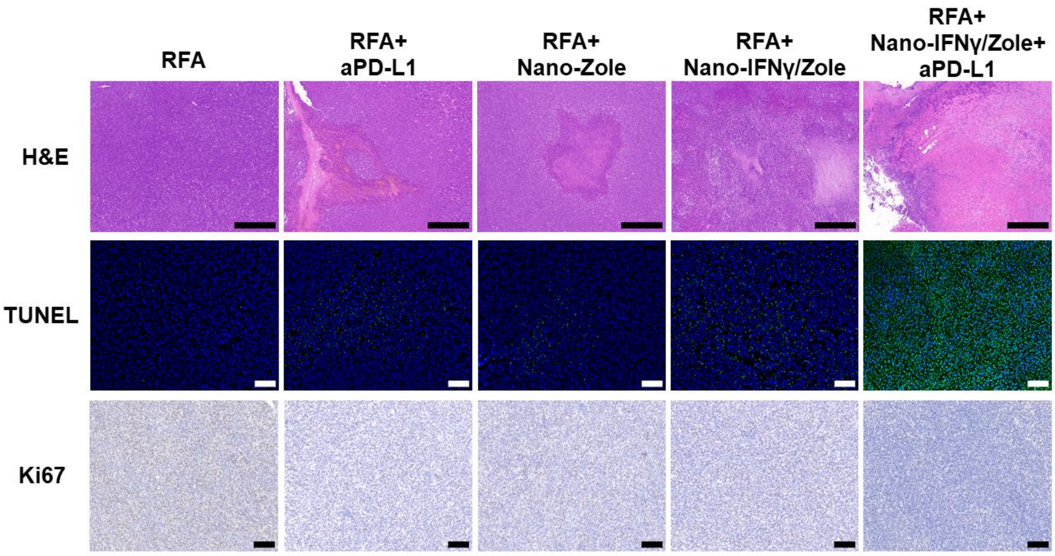

Figure S42 Representative H&E, TUNEL and Ki67 staining images of tumor tissue after different treatment, scale bar for H&E: 100 μm, scale bar for TUNEL: 100 μm, scale bar for Ki67: 100 μm.

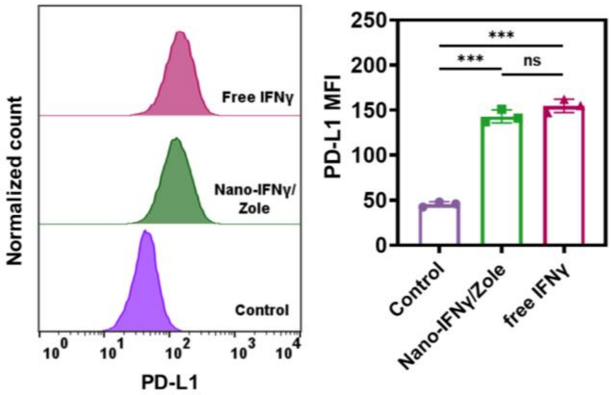

Figure S43 Representative flow cytometry histograms and corresponding quantification of PD-L1 expression on CT26 cells after different treatment (n = 3). All statistical data are presented as mean ± SD; Data were analyzed with two-tailed unpaired t tests; ns, no significance; ***, $p < 0.001$.

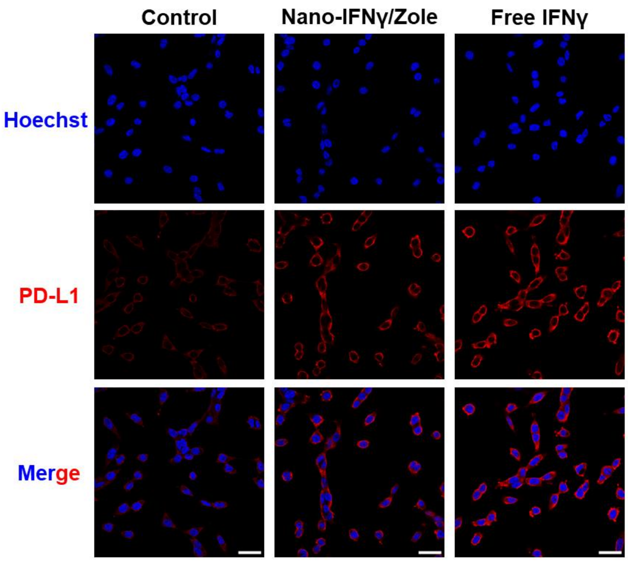

Figure S44 Representative CLSM images of PD-L1 expression on CT26 cells after different treatment, scale bar: 50 μm.

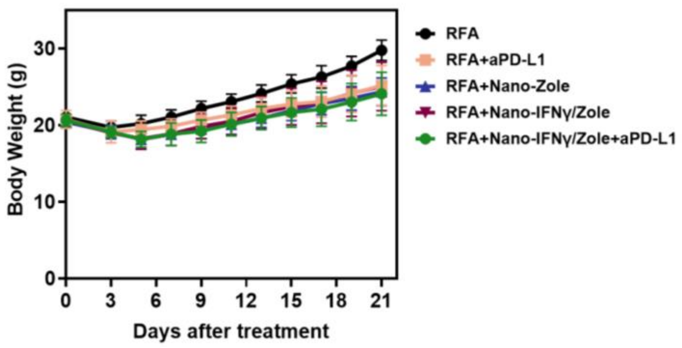

Figure S45 Variation of body weight of tumor-bearing mice after different treatment (n = 5~6).

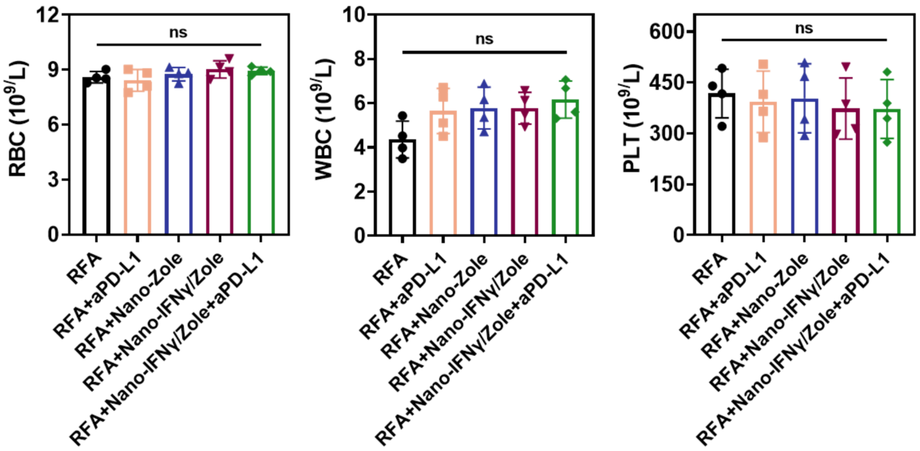

Figure S46 Blood routine detection (BRC, WBC, PLT) of mice after different treatment (n = 4). All statistical data are presented as mean ± SD; Data were analyzed with one-way ANOVA with Tukey's multiple comparisons; ns, no significance.

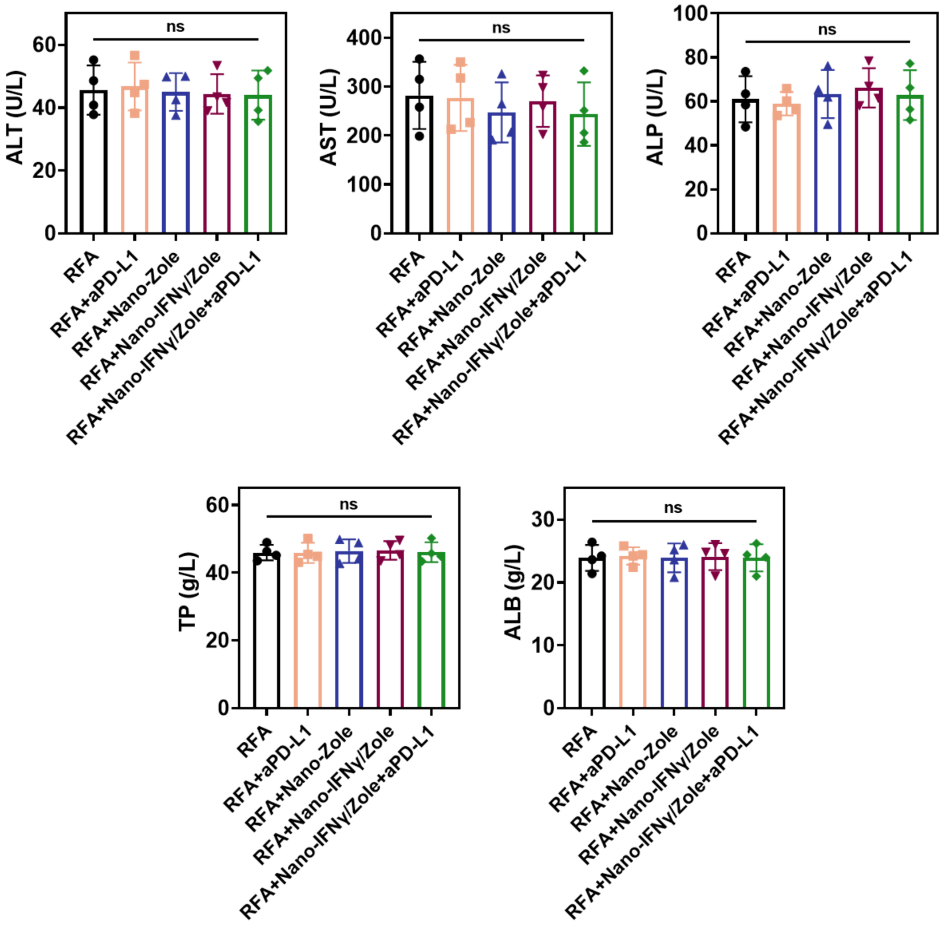

Figure S47 Serum biochemistry analysis detecting liver function of mice after different treatment (n = 4). All statistical data are presented as mean ± SD; Data were analyzed with one-way ANOVA with Tukey's multiple comparisons; ns, no significance.

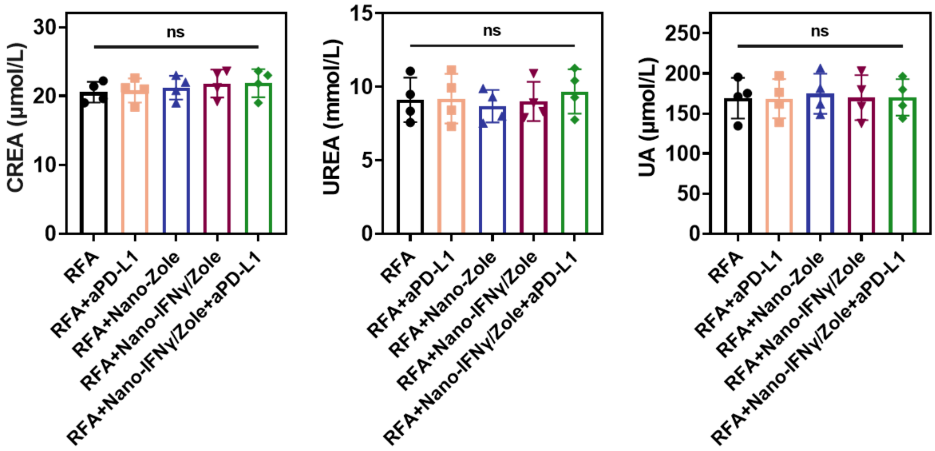

Figure S48 Serum biochemistry analysis detecting kidney function of mice after different treatment (n = 4). All statistical data are presented as mean ± SD; Data were analyzed with one-way ANOVA with Tukey's multiple comparisons; ns, no significance.

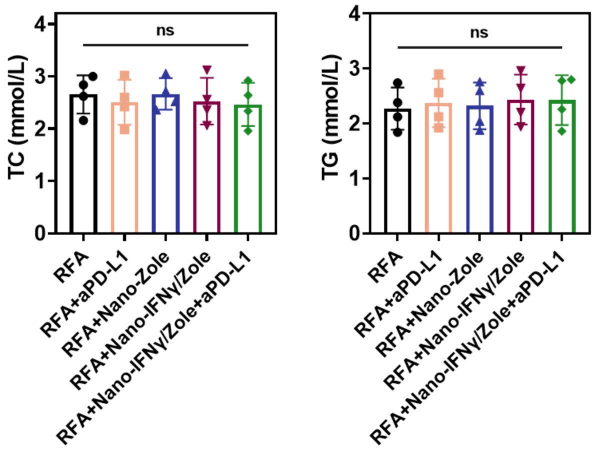

Figure S49 Serum biochemistry analysis detecting serum lipid of mice after different treatment (n = 4). All statistical data are presented as mean ± SD; Data were analyzed with one-way ANOVA with Tukey's multiple comparisons; ns, no significance.

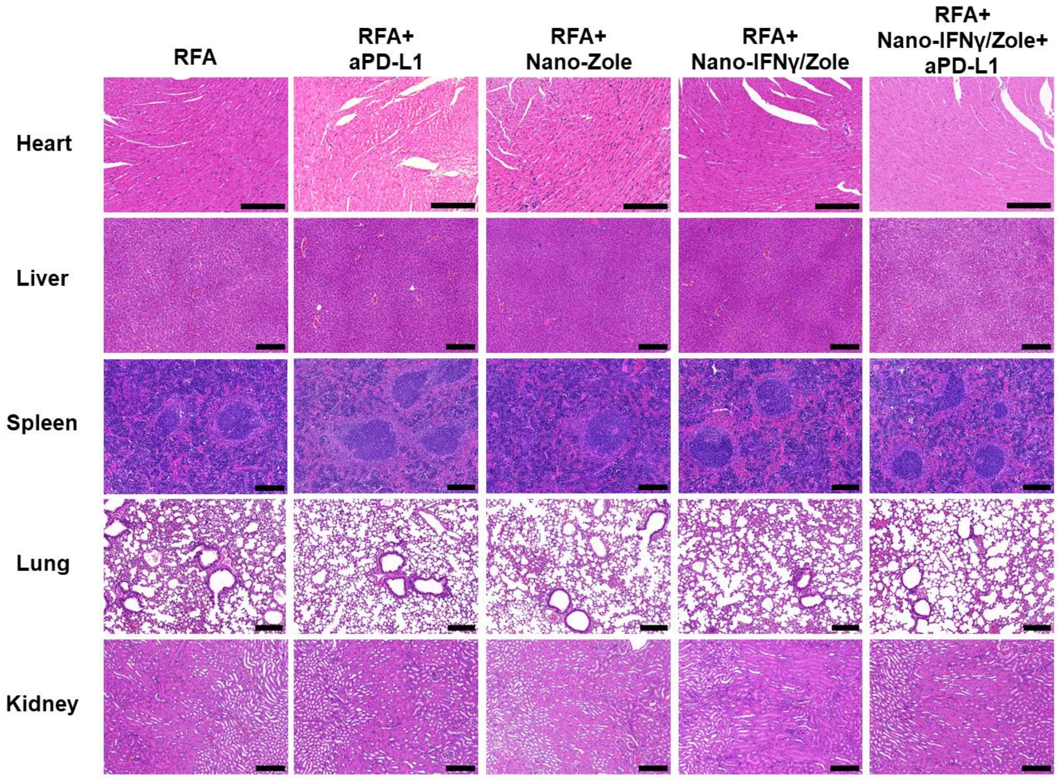

Figure S50 Representative H&E staining of heart, liver, spleen, lung, kidney sections of mice after different treatment; scale bar, 100 μm.

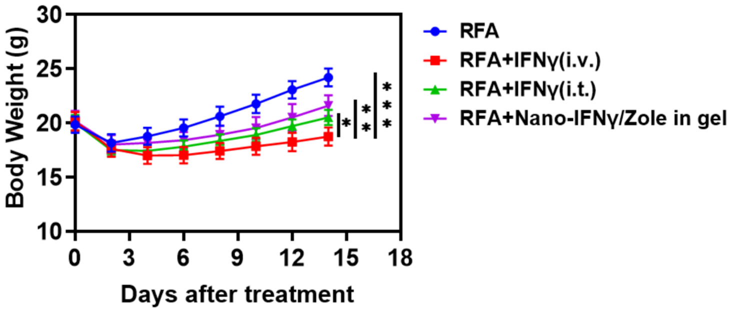

Figure S51 Variation of body weight of tumor-bearing mice after different treatment (n = 5). Data are presented as mean ± SD; The significance of mice body weight on Day 14 were analyzed with two-tailed unpaired t tests; *, $p < 0.05$; **, $p < 0.01$; ***, $p < 0.001$.

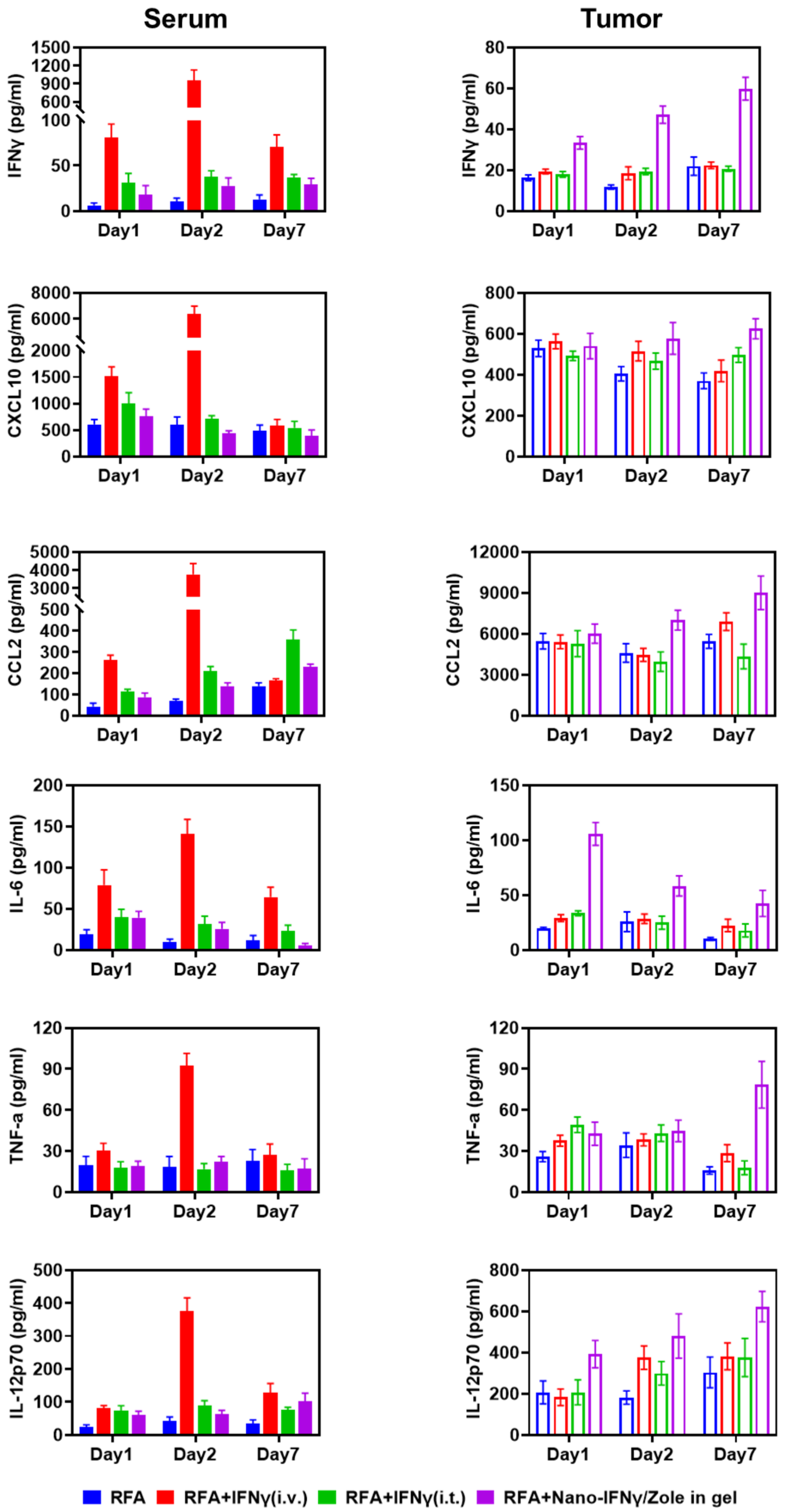

Figure S52 Cytokine levels of the serum and tumor tissue homogenate of iRFA-treated mice at different time points after administration (n = 3). All statistical data are presented as mean ± SD.

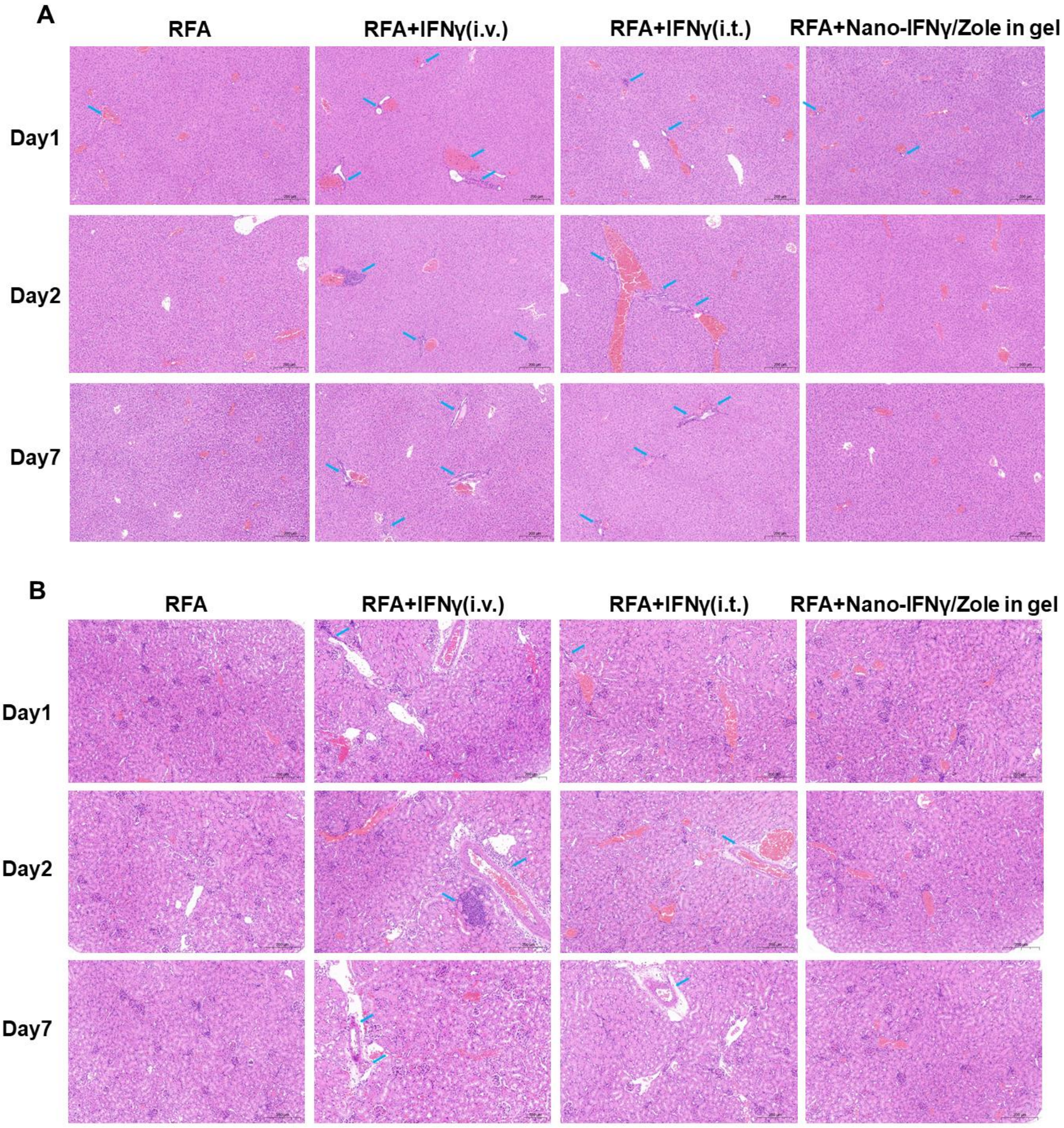

Figure S53 H&E staining of the livers (A) and kidneys (B) of iRFA-treated mice at different time points after administration. Blue arrows indicate infiltrating lesions of inflammatory cells or pathological malignancies of tissues.

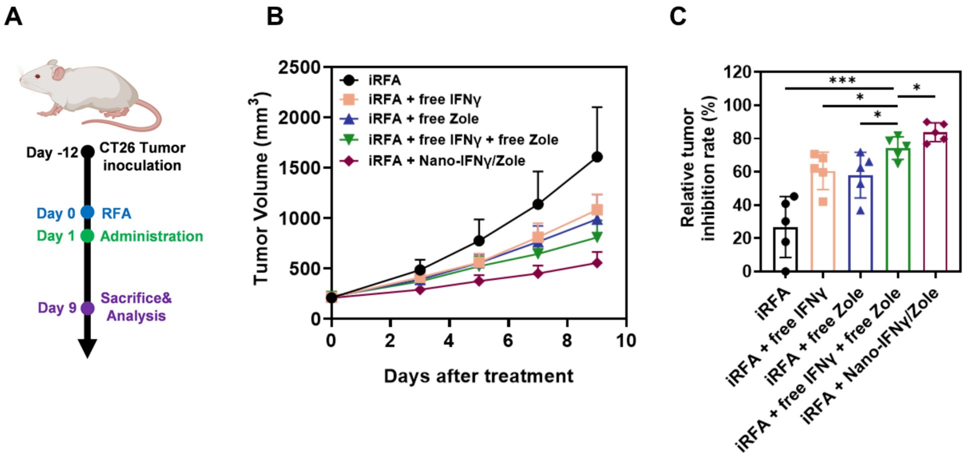

Figure S54 (A) Schematic illustration of the assessment of the *in vivo* anti-tumor efficacy of unmineralized IFNγ or zoledronate after iRFA. (B) Growth curves of tumor volume after different treatment (n = 5). (C) Relative tumor inhibition rate based on tumor weight (n = 5). All statistical data are presented as mean ± SD; data were analyzed with two-tailed unpaired t tests; *, $p < 0.05$; ***, $p < 0.001$.

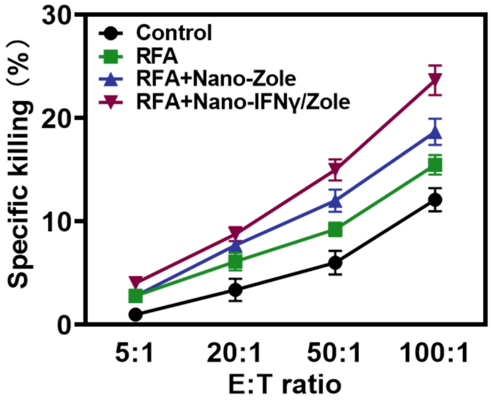

Figure S55 Cytotoxicity of splenocytes to CT26 cells after different treatment (n = 4).

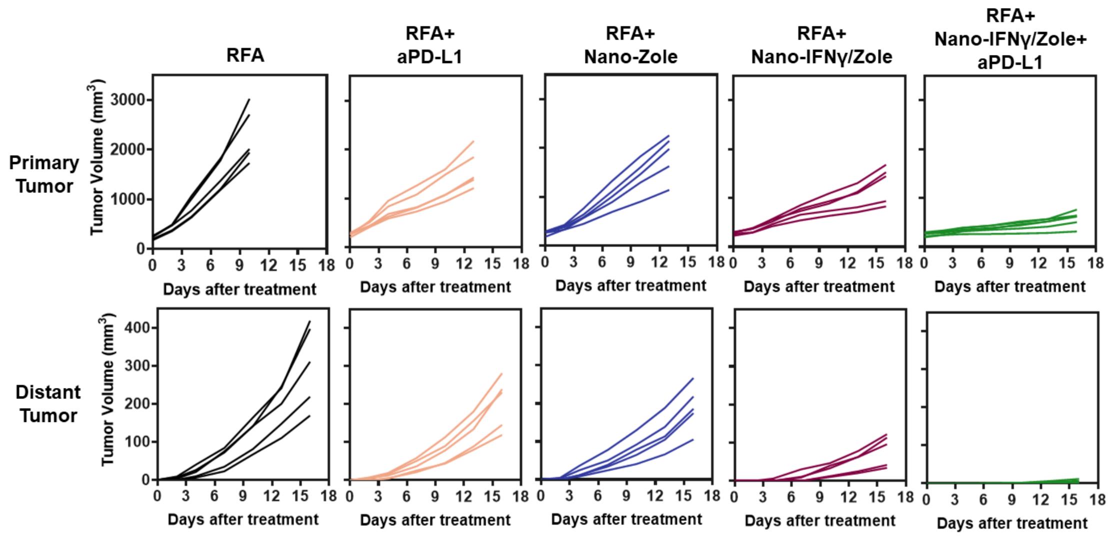

Figure S56 Individual CT26 tumor growth of bilateral tumor-bearing mice after different treatment (n = 5~6).

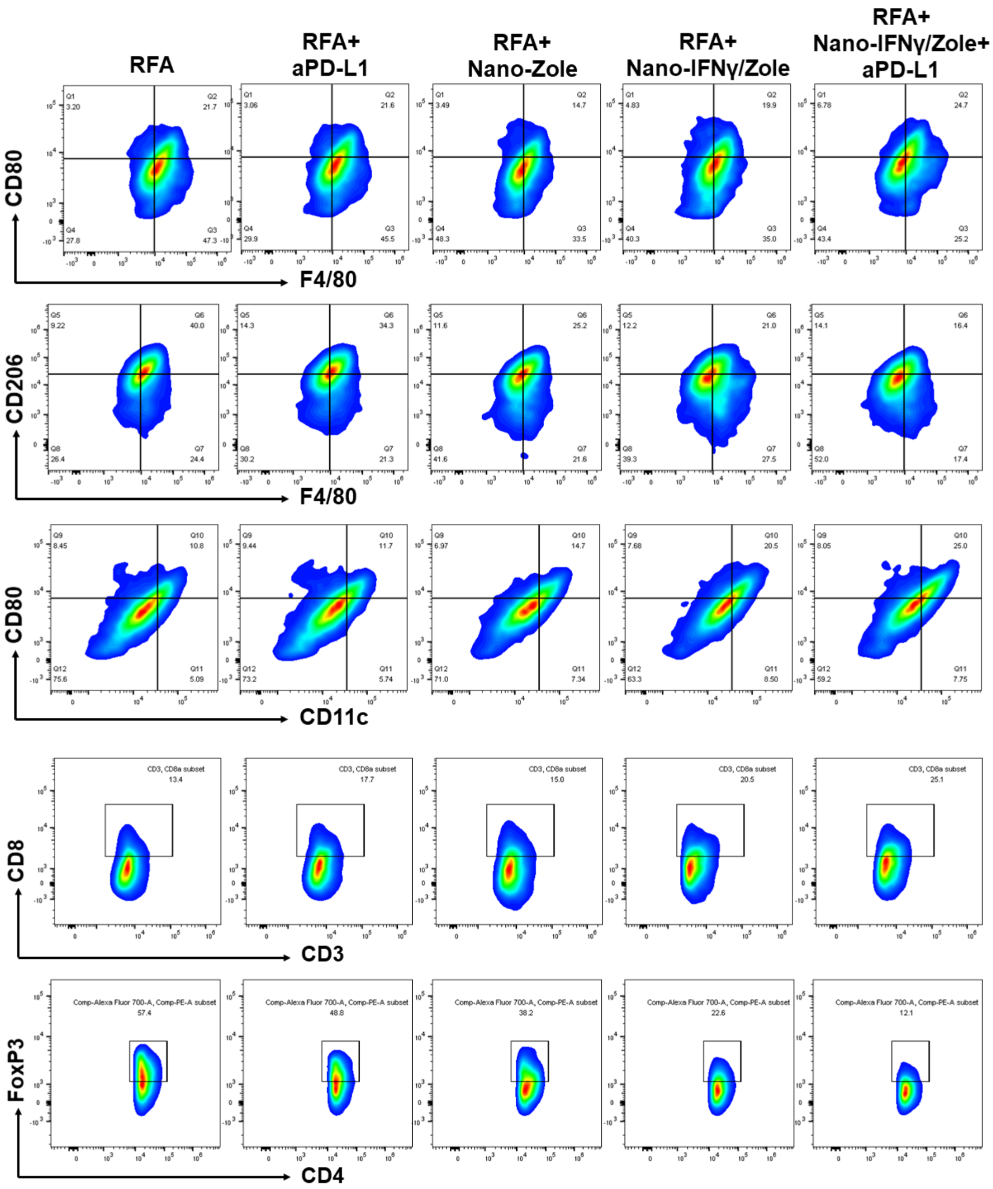

Figure S57 Representative flow cytometry plots of distant tumor-infiltrating M1-TAMs, M2-TAMs, matured DC cells, $CD8^+$ T cells as well as T_{reg} cells after different treatment.

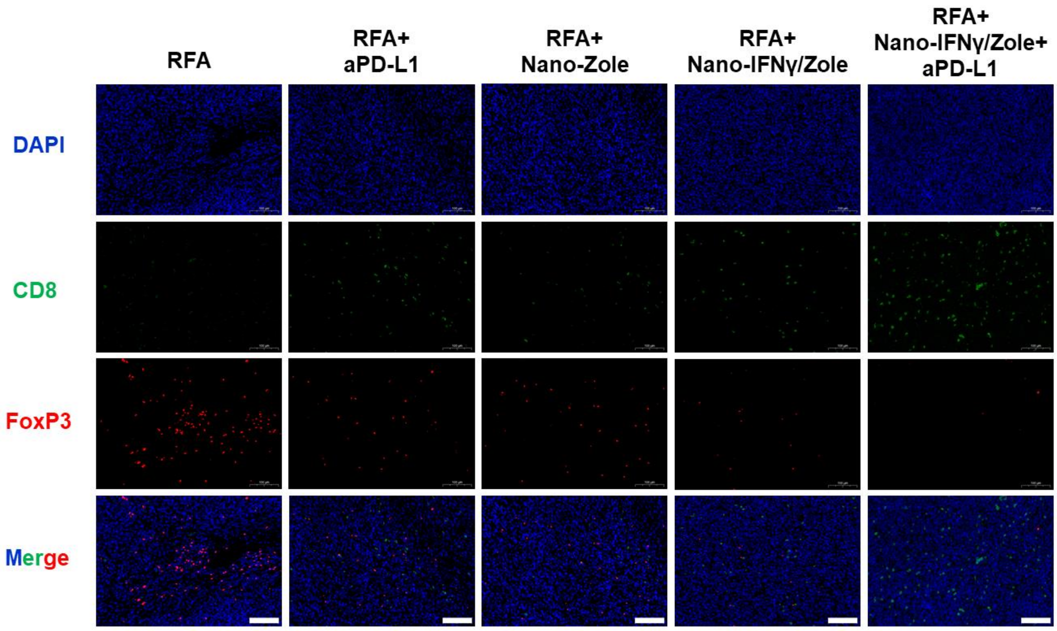

Figure S58 Representative immunofluorescence images of distant tumor-infiltrating T cells after different treatment, scale bar: 100 μm.

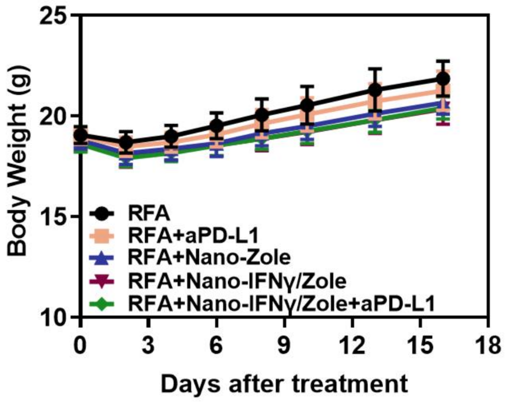

Figure S59 Variation of body weight of bilateral tumor-bearing mice after different treatment (n = 5~6).

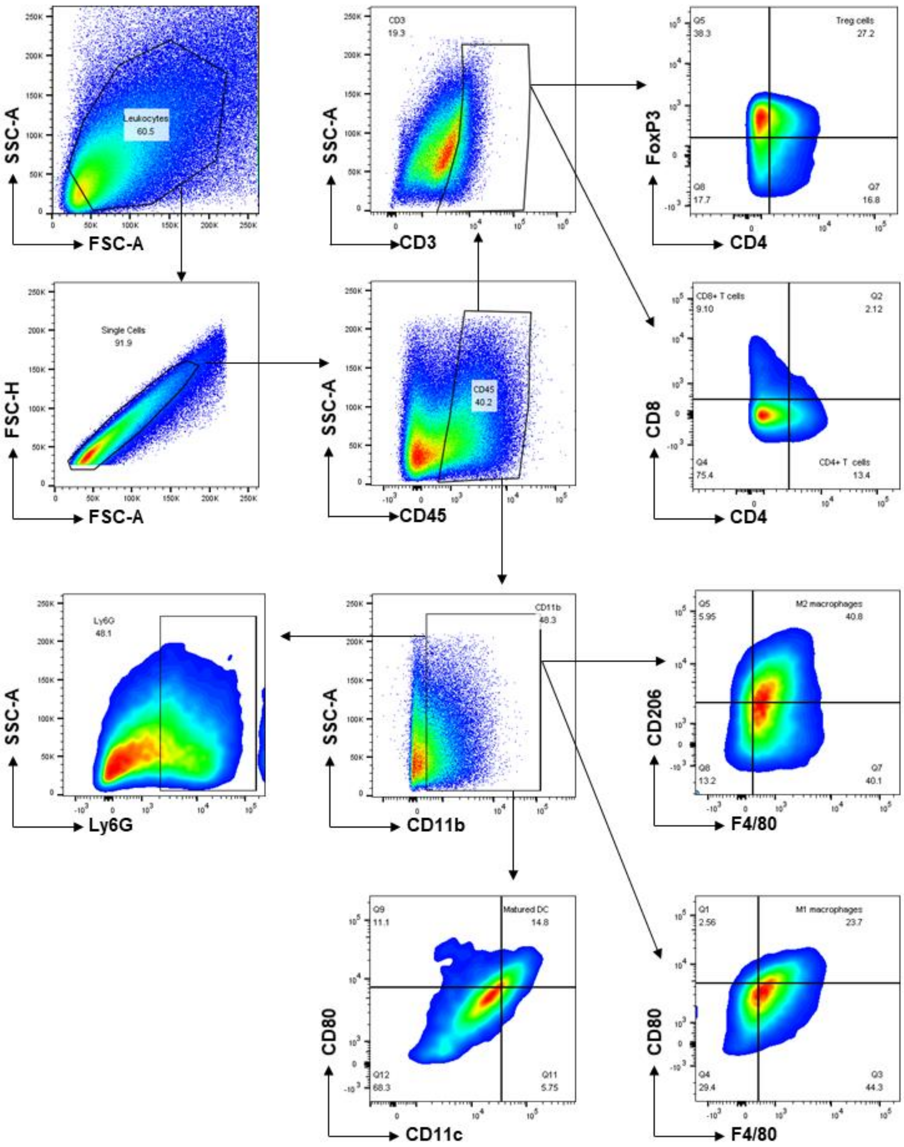

Figure S60 Gating strategy of immune cell subpopulation analysis using FCM.

Table S1 Transcriptomics data of DEGs (original gene counts) in Figure 2I, including genes associated with chemokines & chemokine receptors, cytokines & cytokine receptors, and immune regulation in CT26 tumors 1 day before iRFA (-1 Day) and 2 days after iRFA (2 Day) (n = 3).

Gene Name	-1 Day			2 Day		
	Sample 1	Sample 2	Sample 3	Sample 1	Sample 2	Sample 3
Cxcl1	34	62	71	727	228	87
Cxcl12	424	222	163	1030	697	511
Cxcl14	1661	783	303	2629	2766	1765
Ccrl2	39	48	88	524	148	115
Ccl4	18	9	38	407	119	76
Ccl5	36	18	12	65	110	61
Ccl11	101	101	21	284	209	119
Ccl24	13	4	28	67	55	52
Il1b	221	176	212	2683	721	395
Il1r2	26	11	18	205	69	37
Irf2bp1	1384	730	888	1519	2125	1979
Nos2	203	142	206	1241	382	354
Hspa12b	149	106	79	200	300	241
Atp1b2	35	18	8	77	48	59
Nfkbil1	654	313	496	1013	1158	997
Nfkbia	1388	1700	1528	4660	2559	2877
Tnfrsf8	29	11	21	71	68	49
Isg15	406	394	271	1168	1121	497
Tgfa	472	430	521	396	342	379

Table S2 Transcriptomics data of DEGs (original gene counts) in Figure 2J, including genes associated with chemokines & chemokine receptors, cytokines & cytokine receptors, T cell receptors, and immune regulation in CT26 tumors 2 days after iRFA (2 Day) and 9 days after iRFA (9 Day) (n = 3).

Gene Name	2 Day			9 Day		
	Sample 1	Sample 2	Sample 3	Sample 1	Sample 2	Sample 3
Il2ra	112	192	103	158	193	516
Il2rb	849	1664	726	1320	1241	2008
Ccr2	998	1670	988	2013	2312	1136
Ly6a	858	1238	1062	1568	1085	1391
Cd28	62	67	29	118	159	155
Cd244a	52	57	25	64	134	105
Icos	87	102	50	146	198	245
Cd96	66	103	54	96	111	157
Lag3	20	57	42	75	49	108
Ctla4	56	74	41	127	184	298
Cxcr4	714	633	760	375	230	446
Cxcl14	2629	2766	1765	1267	652	823

Table S3 List of qPCR primer sequences of target genes and reference genes.

Target	Forward primer (5' to 3')	Reverse primer (5' to 3')
Tnfa	GTGCCTATGTCTCAGCCTCTTCTC	GTTTGTGAGTGTGAGGGTCTGG
Nos2	GCCCAACAATACAAGATGACCCTA	ATGATGGACCCCAAGCAAGACT
Nfκb(p65)	CGAGTCTCCATGCAGCTACG	TTTCGGGTAGGCACAGCAATA
Stat1	TGCCTATGATGTCTCGTTTGC	ATCTGTACGGGATCTTCTTGGA
Il10	AATAAGCTCCAAGACCAAGGTGT	CATCATGTATGCTTCTATGCAGTTG
Arg1	CTGGGGATTGGCAAGGTGAT	CAGCCCGTCGACATCAAAG
Stat6	GCCAAAGACCTGTCCATTCG	CCATCTGTTCGGGCTTATAGTG
Irf4	GGAAACTCCGACAGTGGTTGAT	CCTTCTCGGAACTTGCCTTT
Pd1	CCTAGTGGGTATCCCTGTATTGCT	CTTCAGAGTGTCGTCCTTGCTTC
Pdl1	ATTGTAGTGTCCACGGTCCTCC	CAACGCCACATTTCTCCACATC
Gapdh	CCTCGTCCCGTAGACAAAATG	TGAGGTCAATGAAGGGGTCGT
Pcna	GTCGGGTGAATTTGCACGTA	CTCTATGGTTACCGCCTCCTC

Table S4 Peak area of plasma samples of mice at different time points after intravenous injection of free zoledronate (n = 3).

Time	Sample	Peak Area
10 min	Sample 1	0.9870
	Sample 2	1.0889
	Sample 3	1.1024
20 min	Sample 1	1.2531
	Sample 2	1.1705
	Sample 3	1.1805
40 min	Sample 1	1.4278
	Sample 2	1.5782
	Sample 3	1.4393
60 min	Sample 1	1.671
	Sample 2	1.4229
	Sample 3	1.5782

Table S5 Peak area of plasma samples of mice at different time points after intratumoral injection of Nano-IFNγ/Zole in gel (n = 3).

Time	Sample	Peak Area
30 min	Sample 1	N/A
	Sample 2	N/A
	Sample 3	N/A
2 h	Sample 1	N/A
	Sample 2	N/A
	Sample 3	N/A
6 h	Sample 1	2.0063
	Sample 2	2.2889
	Sample 3	2.0007
12 h	Sample 1	2.5877
	Sample 2	2.7957
	Sample 3	2.7144
24 h	Sample 1	2.924
	Sample 2	3.0209
	Sample 3	2.94
48 h	Sample 1	3.0684
	Sample 2	3.0239
	Sample 3	2.9795
168 h	Sample 1	0.4330
	Sample 2	0.5391
	Sample 3	0.4087

Table S6 Transcriptomics data of DEGs (transcript per million) in Figure 4B, including genes associated with cytokines & cytokine receptors, chemokines & chemokine receptors, Toll-like receptors, antigen-presentation receptors, T cell receptors, and immune regulation in CT26 tumors after iRFA and iRFA + Nano-IFNγ/Zole treatment (n = 3).

Gene Name	RFA			RFA + Nano-IFNγ/Zole		
	Sample 1	Sample 2	Sample 3	Sample 1	Sample 2	Sample 3
Ifng	0.15	0.06	0.21	1.13	0.55	0.52
Ifngr2	13.6	16.57	14.47	22.27	36.72	31
Cx3cr1	0.6	1.54	2.52	18.81	21.45	17.13
Cxcl9	5.51	19.67	9.3	32.82	32.22	24.41
Cxcl10	8.04	19.78	20.57	29.5	60.15	58.41
Cxcl11	0.08	0.48	0.31	0.75	1.56	1.55
Ccl12	51.07	123.81	111.12	278.99	172.4	216.77
Ccl24	2.39	2.61	2.31	6.68	11.32	10.48
Ccr1	12.75	15.19	12.99	25.17	36.8	35.54
Ccr2	2.18	7.81	5.66	23.1	19.71	19.2
Csf1	57.47	30.96	25.54	105.22	88.27	75.82
Csf1r	39.18	46.83	40.71	76.26	105.35	84.06
Csf2ra	15.74	18.14	14.55	35.66	39.88	32.12
Csf2rb2	3.23	4.55	3.43	6.22	9.32	12.07
Fasl	0.6	0.42	0.28	1.37	0.93	1.33
Tnfsf8	0.16	0.08	0.15	0.49	1.64	1.49
Tnfsf10	0.33	1.19	1.38	1.72	2.65	2.61
Tnfrsf11a	1.74	2.13	1.1	3.13	2.65	2.69
Tnfrsf21	2.37	1.8	2.15	3.62	4.06	4.8
Traf1	9.82	11.8	9.55	16.48	22.69	24.07
Cd86	3.93	2.75	2.34	7.07	6.6	6.97
H2-Aa	26.12	34.36	29.63	89.03	46.18	53.05
H2-Ab1	30.42	45.57	34.63	98.96	77.68	73.27
H2-DMb1	1.24	2.35	2.22	9.8	7.23	5.29
H2-DMb2	3.07	3.09	2.99	10.55	9.39	9.76
H2-Eb1	17.18	27.57	25.58	79.17	40.12	46.4
Cd8a	0.52	1.25	0.7	5.83	2.67	1.66
Cd8b1	0.35	1.06	0.67	2.95	1.8	1.17
Il27ra	0.36	0.69	0.43	1.41	1.51	1.85
Irf5	7.1	7.9	6	15.86	18.28	16.53
Irf8	11.18	11.28	9.7	25.92	22.71	23.49
Tlr1	1.3	3.02	3.11	4.98	5.58	5.69
Tlr2	5.44	3.77	3.5	7.72	10.28	11.14
Tlr9	0.54	0.97	1.37	2.15	3.55	3.14
Tlr12	0.05	0.06	0.12	0.47	0.58	0.63
Cxcl3	18.16	33.81	19.19	3.19	4.92	5

Cxcl5	1.45	6.91	3.72	0.25	1.06	0.84
Ccl8	241.38	194.28	163.65	100.43	38.65	116.05
Cxcr2	2.17	2.19	1.45	0.49	1.26	1.62
Tgfbr3	36.84	33.39	28.17	25.31	24.51	21.89
Cd163	13.57	8.18	8.24	2.39	1.71	6.48

Table S7. Proteomics data of the differentially expressed proteins (normalized protein abundance) in Figure 6F, including proteins associated with ER function, membrane fusion and vesicle trafficking, ubiquitination and proteasome degradation, antigen presentation and immune activation of BMDMs after Nano-IFNγ/Zole and Nano-IFNγ/Zole + GGPP treatment (n = 3).

Protein Name	Control			Nano-IFNγ/Zole			Nano-IFNγ/Zole + GGPP		
	Sample 1	Sample 2	Sample 3	Sample 1	Sample 2	Sample 3	Sample 1	Sample 2	Sample 3
Hsp90ab1	3418723934	3428647860	3399897657	2411408791	2304476916	2433045705	2731315210	2878962825	2997626152
Hspa4	420266020.6	423706192.6	428219327.8	218359289.5	254953493.1	236878891.9	286053950.5	304779501	313169764.6
Psmc2	94046830.84	87319118.8	98887114.48	52230881.38	54501424.3	61379191.69	70466071.61	68497349.4	64792054.97
Psmd2	283461588.9	274675992.2	283652028.4	174409137.2	186572197.9	161955554.7	191078123.5	231532365.9	203446675.7
Psmd6	114771321.1	112369796.9	108895129.1	70571615.41	69224415.65	69259971.04	91863768.97	95716654.41	83527002.64
Psmd12	94438041.88	91247976.53	101139066.7	63127855.41	67520388.31	65090112.88	73454280.6	81828712.63	74185990.41
Arhgap12	7935003.077	7759484.46	8439724.375	2217305.172	2959511.905	1820362.297	3366179.732	3387962.608	4380990.721
Arhgap17	77147791.23	79141622.2	81921693.58	48745477.84	46941902.37	40703910.65	55571108.25	61331957.77	55722182.96
Arhgap25	56811228.05	54193667.12	58561171.75	32927092.14	34489341.95	32549951.79	44736801.38	46494285.19	41620362.96
Smap1	15195471.47	16326868.38	16568815.5	9814270.423	9180170.773	9952403.908	12245276.73	14220924.01	13182114.79
Arfgap2	24549850.03	24429956.47	26540978.66	11783642.82	12017919.1	13139471.69	16099250.96	17604870.12	17457036.86
Arap1	102777882	97924559.8	101067593.7	52078416.24	54220080.85	44654431.93	67519925.6	71909453.7	77499194.6
Ap2b1	319907604.3	312233465.7	355828328.7	217401517.2	238296593.6	229930331.9	262117223.4	261875194.4	248922251.4
Ap2m1	159190066.1	144707549.7	140108804.9	109074196	98674546.73	118948126.4	120057000.1	127073464.7	140351934.8
Ap5z1	1332377.358	1181591.83	1420688.094	754529.393	724228.3995	846991.57	1068947.894	1263565.69	952326.6929
Ehd4	107751290	112533257.3	120869222.8	64637552.57	60182854.09	67947456.21	79317782.59	84596933.9	74930349.38
Rab12	6281692.127	5222071.037	5549419.672	2590785.184	2424037.459	2355734.245	4139477.562	3866733.782	5688755.319
Eea1	268417492.4	257795894.3	255910275.7	148052097.8	151141984.6	129447436.6	168130552.8	183941001.1	205708901.8
Cltc	3111552062	2748033854	2733458061	1757768993	1825078266	1819855482	2031783215	2188167709	2277135331
Rbsn	1161800.179	877788.0436	1183966.563	274978.5043	318080.6846	322265.8123	561698.1861	739001.0301	837953.3985
Snx1	112850516.6	118143014.7	114834656.5	67767519.02	67286743.18	65737495.51	91318718.07	85476026.45	86054214.16
Snx2	245477908.7	256901438.8	252324011.5	124179223.5	123637108.3	125699071.8	168344533.1	165002068.3	169498806.3
Snx6	62789344.49	59961868.44	62445518.94	34490713.63	29759480.13	36957090.54	47233590.22	36749995.27	42065973.93
Snx17	7344411.677	7413322.503	6600691.891	2802937.48	2295163.579	3338293.53	4058671.369	4867924.256	4385988.04
Vps4b	55930736.67	54843668.16	63195183.24	39802261.45	41957409.21	38591774.08	47667849.47	46181407.34	46474275.69
Vps8	1862594.376	1481926.524	1528725.781	641824.5223	626005.3516	685950.7717	1000474.997	935585.8153	1570082.087
Vps16	37987927.68	35987880.94	34248186.34	20180187.24	22770402.01	20772300.7	24486551.15	26865255.02	27024689.37
Vps39	23957373.1	21279715.11	20829025.38	10915175.35	12461665.61	12406927.65	15500629.09	13974381.83	18237969.36
Vps52	23456192.24	23062512.29	21997829.06	11309936.41	11915793.13	10492299.32	15462775.58	17333327.65	16124223.75
Cbl	65924809.98	59100008.37	70745605.57	31404519.28	38320633.75	35297181.28	49410358.95	48872763.22	50875807.34
Ube2n	340595.1833	364316.5085	415758.5938	183582.5913	161304.0698	193813.3313	246128.9608	261309.5561	289589.6702
Ube4b	10446253.46	10743232.93	10952477.53	3804395.625	4226184.505	4985526.115	6234693.453	8015346.12	6242660.97
Uhrf1	6573209.83	8312854.824	6764928.969	3445126.62	3551861.721	3931204.345	4911190.018	5397658.785	5259771.249
Uba1	735004864.7	741698436.6	760896063.5	425268952.7	468260958.7	500656893	573025325.9	615134723.6	582057660.5
Uba6	32477281.18	28404843.93	22946492.81	12468428.23	8769684.832	8289979.718	22621645.46	20412077.56	15550489.79
Rnf14	5303547.833	4320421.312	3817759.063	2468556.098	2371859.599	2409482.736	3487817.02	3441411.85	3858848.88

Rnf40	6335938.365	5676827.06	5006305.977	2075532.554	1511118.674	1522943.897	4483517.419	4912534.498	4020212.625
Trim25	28252189.87	24367547.26	23392028.96	13306242.75	13089530.23	13928410.67	18638077.59	19109242.56	21025654.7
H2-D1	729593391.8	799619328.6	780600087.4	1617736922	1689112609	1457551198	1323139210	1248840750	1338332580
H2-Q10	2442830.831	1633920.584	2160469.25	7052022.967	6724090.305	7180674.112	4903679.32	5158131.35	6057189.173
Cd40	6582623.414	7796943.252	8892983.813	45842126.75	42410350.41	53725345	50248247.69	51301904.79	45189613.56
Cd86	208454.1186	188913.1654	175507.2188	12016864.52	16302206.25	12633073.8	3457271.219	4753833.746	4879975.636
Alcam	27866911.19	26989495.96	28300939.61	80323902.07	83001236.21	76677983.56	69289642.76	70498020.62	64720010.17
Tlr9	12698142.96	9929394.997	12315932.04	36480601.44	39546699.86	32768588.95	29552811.33	30340327.13	27073436.54
Cd274	11532197.9	9525703.089	11130778.71	53664124.5	54485925.65	60315843.56	37782699.39	40286418.81	40114052.53

References

1. Qin M, Li M, Song G, Yang C, Wu P, Dai W, et al., Boosting innate and adaptive antitumor immunity via a biocompatible and carrier-free nanovaccine engineered by the bisphosphonates-metal coordination. Nano Today. 2021; 37: 101097.

2. Tang M, Chen B, Xia H, Pan M, Zhao R, Zhou L, et al., pH-gated nanoparticles selectively regulate lysosomal function of tumour-associated macrophages for cancer immunotherapy. Nat Commun. 2023; 14: 5888.
